# Inflammation promotes synucleinopathy propagation

**DOI:** 10.1038/s12276-022-00895-w

**Published:** 2022-12-06

**Authors:** Tae-Kyung Kim, Eun-Jin Bae, Byung Chul Jung, Minsun Choi, Soo Jean Shin, Sung Jun Park, Jeong Tae Kim, Min Kyo Jung, Ayse Ulusoy, Mi-Young Song, Jun Sung Lee, He-Jin Lee, Donato A. Di Monte, Seung-Jae Lee

**Affiliations:** 1 Department of Biomedical Sciences, Seoul National University College of Medicine, Seoul, 03080 Korea; 2 Department of Exercise Physiology and Sport Science Institute, Korea National Sport University, Seoul, 05541 Korea; 3 Neuroscience Research Institute, Seoul National University College of Medicine, Seoul, South Korea; 4 Neural Circuits Research Group, Korea Brain Research Institute, Daegu, 41068 Korea; 5 German Center for Neurodegenerative Diseases (DZNE), Bonn, Germany; 6 Department of Biomedical Science and Technology, Konkuk University, Seoul, 143-701 Korea; 7 Department of Anatomy, Konkuk University, Seoul, 05029 Korea; 8 IBST, Konkuk University, Seoul, 05029 Korea; 9 SNU Dementia Research Center, Seoul National University College of Medicine, Seoul, South Korea; 10 Neuramedy Co. Ltd., Seoul, South Korea; 11 Present Address: Nutritional Sciences and Toxicology Department, University of California Berkeley, Berkeley, CA 94720 USA; 12 Present Address: IPS Intellectual Property Law Firm, Seoul, Korea; 13 Present Address: Neuramedy Co. Ltd., Seoul, South Korea

**Keywords:** Parkinson's disease, Parkinson's disease

## Abstract

The clinical progression of neurodegenerative diseases correlates with the spread of proteinopathy in the brain. The current understanding of the mechanism of proteinopathy spread is far from complete. Here, we propose that inflammation is fundamental to proteinopathy spread. A sequence variant of α-synuclein (V40G) was much less capable of fibril formation than wild-type α-synuclein (WT-syn) and, when mixed with WT-syn, interfered with its fibrillation. However, when V40G was injected intracerebrally into mice, it induced aggregate spreading even more effectively than WT-syn. Aggregate spreading was preceded by sustained microgliosis and inflammatory responses, which were more robust with V40G than with WT-syn. Oral administration of an anti-inflammatory agent suppressed aggregate spreading, inflammation, and behavioral deficits in mice. Furthermore, exposure of cells to inflammatory cytokines increased the cell-to-cell propagation of α-synuclein. These results suggest that the inflammatory microenvironment is the major driver of the spread of synucleinopathy in the brain.

## Neurodegenerative diseases: inflammation drives spread of protein aggregates

The growth of protein aggregates that cause neurodegenerative diseases is greatly accelerated by inflammatory immune responses, researchers in Korea and Germany have shown. Diseases such as Alzheimer’s and Parkinson’s show unique patterns of aggregates, which spread via the cell-to-cell transfer of proteins such as α-synuclein. To investigate other factors that may contribute to aggregation, Seung-Jae Lee at Seoul National University, South Korea, and co-workers developed a variant of α-synuclein called V40G, which was largely incapable of forming aggregates in vitro. However, when the team injected V40G into mice, it induced aggregate spreading even more effectively than the wild-type α-synuclein, along with a sustained inflammatory response. Adding an anti-inflammatory agent suppressed the spreading of aggregates and reduced behavioural symptoms in the mice, suggesting that inflammation is a major driver of pathogenic protein aggregation in the brain.

## Introduction

Protein aggregation is the major pathological hallmark of several neurodegenerative diseases, with different types of aggregates and distribution patterns characterizing each disease. Large-scale pathological postmortem examinations have indicated that protein aggregates in Alzheimer’s and Parkinson’s diseases spread from a few initial sites and progressively involve an increasing number of brain regions in a highly specific topographic sequence^1,2^. This spreading of pathological aggregates most likely occurs *via* direct cell-to-cell transfer of aggregation-prone pathogenic proteins, such as tau and α-synuclein^3–6^.

Although the mechanism remains unclear, the aggregate spreading phenomenon has been verified in several model systems. The most commonly used animal model for studying aggregate spreading is mice injected intracerebrally with preformed fibrils (PFFs). A single injection of PFF causes aggregation of the corresponding protein (e.g., tau or α-synuclein) in different brain regions^7^. Features underlying the transmission of prion proteins have led researchers to presume that aggregate spread in Alzheimer’s disease and Parkinson’s disease also occurs by a mechanism known as templated conformational seeding^8^. However, this theory is apparently inconsistent with some experimental observations. For example, PFFs are rapidly cleared after injection, and there is always an incubation time before aggregates reappear^7^. Furthermore, injection of an α-synuclein variant lacking the critical region for fibrillation still resulted in α-synuclein aggregate spreading^9–11^. Thus, mechanisms other than templated seeding may contribute to the spread of protein aggregates. Here, we directly tested whether templated seeding is sufficient to explain protein aggregate spreading by using a specific sequence variant of α-synuclein (V40G) that cannot seed aggregation.

## Materials and methods

### Mutagenesis

A mutation (V40G) was introduced into human wild-type α-synuclein (α-synuclein/pDual GC; Agilent Technologies, Santa Clara, CA, USA, #214503) using a QuikChange II XL Site-Directed Mutagenesis Kit (Agilent Technologies, #200521). The primers used in this study are listed in Supplementary Table 5.

### Protein purification and fibril preparation

WT-syn and V40G were expressed and purified as previously described^12^. Fibrillation was performed as previously described^12^. Human or mouse α-synuclein (200 μM in PBS) was incubated at 37 °C for 9 days with constant shaking at 1,050 rpm in the presence or absence of seeds.

### Circular dichroism (CD) spectroscopy

CD analysis was performed as previously described^12^. All spectra were obtained by averaging 10 separate measurements.

### Transmission electron microscopy (TEM)

TEM was performed as previously described^12^. The aged WT-syn and V40G were observed using a JEM1010 transmission electron microscope (JEOL, Akishima, Tokyo, Japan).

### Fluorescent dye binding assay

Recombinant human or mouse α-synuclein samples were mixed with either 10 μM thioflavin T (Sigma, #T3516) solution in glycine (pH 8.5), 0.001% Sybr Green commercial stock solution (#S7563, Invitrogen, Carlsbad, CA, USA), 50 μM X-34 (Sigma, #SML1954), or 50 μM curcumin (Sigma, #C1386). After incubation at room temperature, fluorescence measurements were performed on a Synergy NEO plate reader (Biotek, Winooski, VT, USA). Excitation and emission wavelengths were set at 440 nm and 490 nm, respectively, for thioflavin T, at 485 nm and 520 nm for Sybr Green, at 380 nm and 520 nm for X-34, and at 440 nm and 519 nm for curcumin.

### Sedimentation assay

Twenty microliters of sample (200 μM in PBS) was mixed with 280 μl of DPBS (Gibco, Carlsbad, CA, USA, #A1285601) and placed on top of 30% sucrose, with the bottom of the tube containing 5% sucrose. Samples were centrifuged at 38,000 rpm in a Beckman XL-90K ultracentrifuge using an SW-41Ti rotor (Beckman). Fractions were collected and mixed with Laemmli sample buffer.

### Proteinase K (PK) digestion

α-Synuclein samples (5 µM) were used for PK digestion. PK digestion was performed as previously described^12^. The remaining band intensity after PK digestion was quantified using Multi Gauge (v.3.0) software (Fujifilm, Akishima, Tokyo, Japan). Bars represent the mean ± SEM (*n* = 3 per type of aggregate).

### Protein misfolding cyclic amplification (PMCA)

PMCA was performed as previously described^12^. Fifteen nanograms of aged human WT-syn or V40G was added as a source of exogenous seeds. The equipment for PMCA comprises a microplate horn (Qsonica, Newtown, CT, USA, #431MPX), sound enclosure (Qsonica, #432MP), and thermoelectric chiller (Qsonica, #4900). PMCA was carried out using recombinant human α-synuclein monomers as substrates. α-Synuclein monomers were prepared at a final concentration of 5 µM in conversion buffer (1% Triton X-100, 150 mM NaCl), and a 50 µl aliquot was transferred into a PCR tube containing three Teflon beads. Samples were subjected to 48 cycles of 20 s sonication (amplitude 1%) and 29 min 40 s incubation at 37 °C for 24 h.

### Western blotting

Western blotting was performed as previously described with a few modifications^12,13^. The antibodies used in this study are listed in Supplementary Table 6. Image detection was performed using an Amersham Imager 600 (GE Healthcare) and Multi Gauge (v.3.0) software (Fujifilm, Akishima, Tokyo, Japan).

### Animals

Ten-week-old male wild-type C57BL/6 mice were purchased from The Jackson Laboratory (Bar Harbor, ME, USA). Three-month-old C57BL/6 mice overexpressing human α-synuclein under the murine Thy1 promoter (mThy1-α-syn tg, Line 61) were used for the immune cell infiltration assay^14^. Mice were housed and processed according to the standardized conditions at the animal facility of the Seoul National University College of Medicine. All mice were maintained in the animal facility for habituation for at least one week before the start of the experiment. All mouse studies were conducted in compliance with the relevant ethics regulations and approved by the Seoul National University Ethics Committee (IACUC SNU-170428).

### Stereotaxic injection of WT fibrils and V40G multimers

For intrastriatal injection of human WT-syn fibrils and V40G multimers, 10-week-old male C57BL/6 mice were anesthetized with ketamine hydrochloride and xylazine hydrochloride (3.5:1, 2.5 µl/g). PBS, WT-syn fibrils or V40G multimers (6 µg) in a volume of 2 µl were stereotaxically injected into the right striatum (anterior/posterior, 1.0 mm; medial/lateral, 1.5 mm; and dorsal/ventral, 3.0 mm) at a speed of 0.5 μl/min using a 30 G needle.

### Drug administration

The administration of 2 mg/kg or 40 mg/kg acetylsalicylic acid (Aspirin; Sigma, #A5376) in drinking water was initiated 1 week after intrastriatal injection of PBS, WT-syn, or V40G. The consumption of aspirin was monitored by measuring the volume of the remaining solution once every 2 weeks for 18 weeks; the rate of consumption was 4.3–5.2 ml/day, and there was no significant difference in consumption between groups. The concentration of aspirin dissolved in drinking water provided for each animal was 12.5 µg/ml (2 mg/kg) or 250 µg/ml (40 mg/kg).

### Behavioral assessments

We subjected mice to tests of total activity, motor control, motor strength, sensory spatial memory and emotional behavior 1 week prior to sacrifice. Behavioral tests were performed using a computerized video recording and tracking system (EthoVision XT version 14, Noldus, Wageningen, Netherlands).

#### Open field test

To assess activity, locomotion, and anxiety, we subjected mice to the open field test as previously described^15^.

#### Rotarod test

Motor coordination and balance were assessed with an accelerating rotarod system (Rotamex 5, Columbus Ins., Columbus, OH, USA). Mice were placed on the rotarod spindle (3.0 cm × 9.5 cm), which accelerated from 4 to 35 rpm over 300 s, and the latency to fall was measured. After one practice trial using this protocol, each mouse was tested twice, and the average latency was taken.

#### Four-limb hanging test

The four-limb hanging test was used to measure the muscle strength of the four limbs. Mice were placed on a grid apparatus (5 × 5 cm) made from wire mesh of such a gauge that the mice could grip the wires. The mice were placed on top of the grid, which was then inverted 180 degrees. The latency for the mice to fall from the grid was measured, with a maximum of 600 s.

#### Y maze test

To measure the prefrontal cortex- and hippocampus-dependent spatial memory deficits^16^, we tested the spontaneous alternation behavior (SAB) and continuous alternation (CA) of mice using a standard protocol^17^.

### Sample collection

At 2, 4, 10 or 19 weeks after intrastriatal injection, mice were anesthetized with ketamine hydrochloride and xylazine hydrochloride (3.5:1, 2.5 µl/g) and then transcardially perfused with saline followed by ice-cold 4% PFA. Brains were dissected out and fixed in phosphate-buffered 4% PFA for at least 48 h at 4 °C for neuropathological analysis. For biochemical analysis, brain samples were dissected and stored at −80 °C at 0, 2, 7, and 14 days and 5 and 19 weeks after intrastriatal injection. Four weeks after intrastriatal injection, the brain samples were dissected into the rhinal cortex before freezing on dry ice and stored at −80 °C for RNA analysis.

### Evans blue assay

Two weeks after intrastriatal injection, mice were anesthetized with a ketamine/xylazine mixture, and cardiac perfusion was performed using 50 ml PBS (pH 7.2) followed by 50 ml of a cocktail containing 1% Evans blue (Sigma–Aldrich) dissolved in 4% PFA. Brains were dissected out and fixed in phosphate-buffered 4% PFA for 4 h, cryoprotected in 30% sucrose overnight at 4 °C, and then frozen in OCT medium on dry ice. Twenty-micrometer-thick brain cryosections were mounted on fluorescent mounting medium containing DAPI (H1200, Vector Laboratories, CA, USA). Then, the cells were visualized using a fluorescence microscope (Olympus IX53) by excitation with 543-nm laser beams and visualized as red fluorescence.

### Immunohistochemistry and neuropathological analysis

The procedures for immunohistochemical experiments have been described in detail elsewhere^18^. The antibodies used in this study are listed in Supplementary Table 6. Image detection was performed using a ZEISS AX10 microscope and an Aperio AT2 microscope. The levels of immunoreactivity were determined by optical density analysis using ImageJ (NIH) and corrected against background signal levels. Phospho-α-synuclein-positive cells in each animal were quantified as the percentage of positive cells in a field of view based on cell body recognition using the ImageJ program (NIH).

### Immunofluorescence and thioflavin S staining

Free-floating brain sections were blocked with 4% BSA in PBST and then reacted with primary antibodies and fluorescent dye Alexa 488- or rhodamine red-X-conjugated secondary antibodies and mounted with fluorescence mounting medium (Vector Laboratories). The antibodies used in this study are listed in Supplementary Table 6. The phospho-α-synuclein immunoreactive sections were treated with graded EtOH for hydration and incubated in filtered 1% aqueous thioflavin S (Sigma, T1892) for 8 min. After washing with 80% and 95% EtOH, the coverslip was placed in aqueous mounting medium, and the slides were dried in the dark overnight. The stained samples were observed under a Carl ZEISS-LSM 700 confocal laser-scanning microscope.

### Immuno-EM

Mouse brain slices were fixed using 2.5% glutaraldehyde and 2% paraformaldehyde in sodium cacodylate buffer (pH 7.2) at 4 °C. Samples were fixed again by using 1% osmium tetroxide for 30 min at 4 °C. The fixed samples were dehydrated using an ethanol series (50%, 60%, 70%, 80%, 90%, and 100% ethanol) for 20 min and were transferred to LR White (Electron Microscopy Science, Hatfield, PA, USA). The samples were impregnated with and embedded in the same resin mixture, sectioned (60-nm-thick sections) with an ultramicrotome (Leica Ultracut UCT; Leica Microsystems, Vienna, Austria), and placed on nickel grids. α-synuclein fibrils in the samples were labeled with immunogold by using phospho-α-synuclein antibody and 9- to 11-nm colloidal gold-conjugated goat anti-mouse IgG secondary antibodies (Sigma, St. Louis, MO, USA). The sections were double-stained with 2% uranyl acetate for 10 min and lead citrate for 5 min and viewed under a transmission electron microscope at 120 kV (Tecnai G2, Thermo Fisher, Waltham, MA, USA).

### Nigral tissue preparation and neuronal cell counting

Forty-micrometer-thick free-floating brain sections were processed for immunohistochemistry as previously described^19^. Rabbit anti-TH antibody (Millipore, Burlington, MA, USA) was used as the primary antibody prior to incubation in biotinylated goat anti-rabbit secondary antibody solution (Vector laboratories, #BA1000; 1:200). The sections were treated with avidin-biotin-peroxidase (ABC Elite kit, Vector Laboratories) complex. The color reaction was developed by DAB. Sections were counterstained with cresyl violet (FD Neurotechnologies) and coverslipped using DPX mounting media (Sigma, #06522). Unbiased stereological counts were performed by an investigator blinded to the experimental groups using the optical fractionator and the Stereo Investigator 2019 software (MBF, version 1.3). The ipsilateral substantia nigra pars compacta was delineated on every 4^th^ midbrain section in the rostrocaudal axis between Bregma −2.7 mm and −3.6 mm. Large and densely packed tyrosine hydroxylase-immunoreactive neurons characterized this midbrain region, allowing its delineation using a low-power objective lens (4X UPlanFL N) on an Olympus BX53 microscope equipped with an automated stage (MBF, mac6000) and a Heidenhein Z-axis decoder. Counts were performed at higher magnification (100X UPlanS Apo) using a 1-µm guard zone on the top and bottom of each section. The coefficient of error was calculated according to Gundersen and Jensen^20^; all values were <0.10.

### Brain tissue and cell extraction

Samples were homogenized with lysis buffer (1% Triton X-100, 1% (v/v) protease inhibitor cocktail (Sigma‒Aldrich, P8340) in PBS. Ten milligrams of tissue was lysed in 1 ml of lysis buffer. Lysates were incubated on ice for 10 min and centrifuged at 16,000 × g for 10 min. Triton X-100-insoluble fractions were resuspended with 1X Laemmli sample buffer in a half-volume of lysis buffer and sonicated briefly.

### RNA processing

RNA was extracted using TRIzol (Invitrogen, #15596018) and quantified using an ND-2000 Spectrophotometer (Thermo Fisher Scientific). Libraries were prepared using the QuantSeq 3’ mRNA-Seq Library Prep Kit (Lexogen, Inc., Greenland, NH, USA) and sequenced with 75-bp single-end reads on a NextSeq 500 (Illumina, Inc., San Diego, CA, USA).

### RNA sequencing (RNA-seq) data analysis

Analysis of the RNA-seq data was performed using TopHat2 (version 2.1.1) and the Cufflinks suite (version 2.1.1). The data were processed and quantified using HTSeq. Differential gene expression analyses were performed using DESeq2 (version 1.24.0)^21^. *p* < 0.05 was used as the threshold for differentially expressed genes (DEGs). Enrichment analyses of GO terms were analyzed using the Cytoscape plug-in ClueGO based on related terms and statistical significance^22^. The enriched Gene Ontology (GO) and Kyoto Encyclopedia of Genes and Genomes (KEGG) pathway analyses of the DEGs were conducted on DAVID datasets (version 6.8)^23^.

### Primary microglial culture

Primary microglial cells were obtained from the cerebral cortices of 1-day-old neonatal C57BL/6 mice as previously described^24^. Approval for the experiments was granted by the Institutional Animal Care and Use Committee of Seoul National University (SNU-171207-2-5). To induce inflammatory cytokines in microglial cells, microglial cells were treated with 200 nM human-aged WT-synuclein or V40G-synuclein for 8 h for qRT‒PCR and for 18 h for ELISA.

### Measurement of cytokine secretion

Microglial TNF-α and IL-1β secretion was measured in cell supernatants using the Mouse TNF-alpha Quantikine ELISA Kit (SMTA00B, R&D Systems, Minneapolis, MN, USA) and Mouse IL-1 beta/IL-1F2 Quantikine ELISA Kit (SMLB00C, R&D Systems) according to the manufacturer’s protocols.

### Immunofluorescence staining

The procedure for immunofluorescence staining was performed as previously described^25^. Anti-ASC rabbit monoclonal antibody (Cell Signaling Tech, MA, USA) was used as a primary antibody. Images were obtained using a Zeiss LSM 700 confocal laser scanning microscope.

### Reverse transcription-quantitative PCR

Total RNA was extracted using the RNeasy Mini Kit (Qiagen, Hilden, NRW, Germany, #74106) and reverse-transcribed using the iScript cDNA synthesis kit (Bio-Rad, #1708891). Target genes were amplified using iTaq Universal SYBR Green Supermix (Bio-Rad, #172-5121) with specific primers. The primers used in this study are listed in Supplementary Table 5. Relative mRNA levels were calculated according to the 2^−ΔΔCT^ method. All ΔCτ values were normalized to glyceraldehyde-3-phosphate dehydrogenase.

### Degradation kinetics of aged WT-synuclein or V40G-synuclein in microglia

Primary mouse microglial cells were treated with either 200 nM human-aged WT-synuclein or V40G-synuclein for 30 min at 37 °C. After washing with ice-cold PBS, the cells were incubated with fresh growth media at 37 °C and harvested at the indicated times.

### Cell-to-cell propagation assay

For coculture with microglia, stable V1S and SV2 cells^26^ were cocultured for 1 day. The next day, microglial cells were added to the V1S/SV2 mixture. Cells were cultured for 2 additional days. For treatment with recombinant inflammatory cytokines, a mixture of V1S and SV2 cells was cultured for 3 days. Recombinant human TNF-α (Prospec, Ness-Ziona, Israel, #CYT223; 50 ng/ml) or IL-1β (Prospec, #CYT208, 50 ng/ml) was added to V1S and SV2 coculture at a final concentration of 50 ng/ml for the last 24 h. Cells were fixed in 4% paraformaldehyde in PBS prior to nuclear staining with TOPRO-3 iodide (Invitrogen). Images were acquired by confocal microscopy (Zeiss LSM700, 63X).

### Statistical analysis

All experiments were performed in a blinded manner and at least in duplicate. Differences were considered significant at *p* < 0.05 and were calculated using paired, two-tailed Student’s t tests, one-way ANOVA with Tukey’s post hoc test, and two-way ANOVA with Bonferroni’s post hoc test using GraphPad Prism **7**.04 and 9.0.2 (GraphPad Software Inc., La Jolla, CA, USA). Pearson’s correlation analysis was performed to analyze the linear correlation between two variables. The values in the figures are expressed as the mean ± standard error of the mean (s.e.m.).

## Results

### The α-synuclein V40G variant forms amyloid fibrils much less efficiently than WT and blocks fibril formation in vitro

**Fig. 1:**
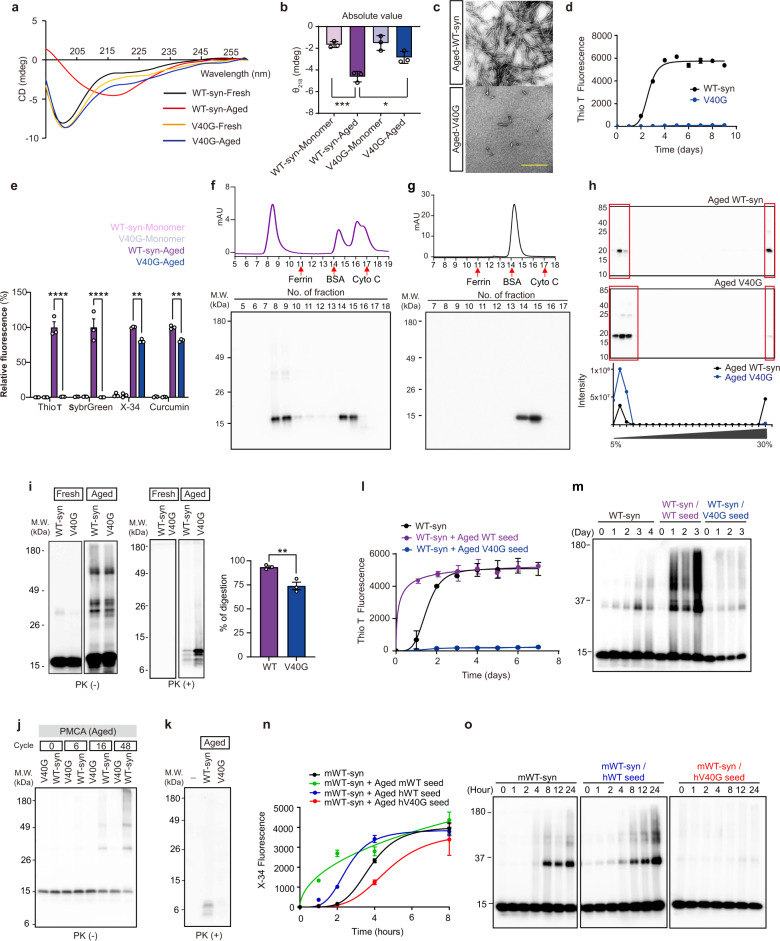
Characterization of the V40G variant of α-synuclein. **a** CD spectroscopy of human WT-syn monomer, V40G-syn monomer, aged WT-syn, and V40G. **b** Comparison of CD data at 218 nm. **c** TEM image of human aged WT-syn (top) and V40G (bottom). Scale bars, 0.4 μm. **d** Thio T binding kinetics of WT-syn or V40G recombinant α-synuclein over 9 days. **e** Dye binding assays of fresh/aged WT-syn and V40G. **f** Size exclusion chromatography (top) and western blotting (bottom) of aged V40G. Cyto C: Cytochrome C. **g** Size exclusion chromatography (top) and western blotting (bottom) of the V40G monomer. Cyto C: Cytochrome C. **h** Ultracentrifugation assay. Western blotting (top) and quantification (bottom). **i** Western blots and quantification of WT-syn and V40G without (left) or with PK digestion (right). Note that the size ranges of the left and right blots are different. **j** Western blotting of PMCA end-products without PK digestion. Human-aged WT-syn or V40G was used as a seed, and human fresh WT-syn was used as a substrate for the PMCA reaction. **k** Western blotting of PMCA end-products with PK digestion. **l, m** Thio T binding kinetics of recombinant human α-synuclein with human aged WT-syn or aged V40G as seeds. In all panels, “fresh” indicates pure monomers, and “aged” indicates the protein samples incubated for 9 days at 37 °C with constant agitation. **m** The results of the Thio T binding assay were confirmed by western blotting. **n, o** X-34 binding kinetics of recombinant mouse α-synuclein with aged human WT-syn or aged human V40G as seeds. In all panels, “fresh” indicates pure monomers, and “aged” indicates the protein samples incubated for 3–4 days at 37 °C with constant agitation. **o** The result from the X-34 binding assay was confirmed by western blotting. Significance was assessed by one-way ANOVA with Tukey’s post hoc comparison between groups (**b**) or by two-tailed unpaired Student’s t test (**e, i**), *P < 0.05, **P < 0.01, ***P < 0.0001. All data are presented as the mean ± SEM.

To find a sequence variant without seeding ability, we screened a pool of α-synuclein mutants in the hinge region (amino acids 38–44) for variants unable to form amyloid fibrils after 9 days of incubation and identified a variant with a substitution of glycine for valine 40 (V40G). Circular dichroism (CD) spectra indicated that “fresh” human wild-type α-synuclein (WT-syn) and V40G monomeric proteins showed random coil configurations (Fig. 1a). To determine the behavior of the proteins after a period of aging, we incubated them for 9 days. After incubation, human “aged” WT-syn acquired a β-sheet-rich conformation (Fig. 1a) with filamentous morphology (Fig. 1c, Supplementary Fig. 1a) and exhibited thioflavin T (Thio T) fluorescence (Fig. 1d), reflecting the structural transition to amyloid fibrils. In contrast, aged V40G contained much less β-sheet content than WT (Fig. 1a, b) and consistently exhibited minimal Thio T fluorescence (Fig. 1d). To further characterize the conformational differences between WT-syn and V40G, we performed additional dye binding assays with SybrGreen, X-34, and curcumin. SybrGreen is capable of detecting amyloid structures that cannot be visualized with Thio T. X-34 is a Congo red derivative that binds to amyloid fibrils in a polymorphism-independent manner^27^. Curcumin can sense oligomers as well as fibrils^28^. Similar to Thio T, SybrGreen bound WT-syn fibrils but did not bind V40G multimers. WT-syn fibrils and V40G multimers also showed significantly different binding to X-34 and curcumin. None of these dyes showed significant binding to monomeric forms of these proteins. These results show that V40G multimers had different dye binding properties from WT-syn fibrils (Fig. 1e). Size exclusion chromatography showed that a large portion of V40G was multimeric after the 9-day incubation (Fig. 1f), while “fresh” V40G was exclusively monomeric (Fig. 1g). Electron microscopy (EM) confirmed that V40G formed a variety of structures, including short fibril-like structures and small multimers with heterogeneous morphologies (Fig. 1c, Supplementary Fig. 1b). In addition, V40G samples show some typical fibrils, which would explain the presence of a small amount of β-sheet signal in the CD spectrum (Fig. 1b). Velocity ultracentrifugation confirmed that V40G formed fewer large sedimenting aggregates than WT, while the monomer showed a similar pattern (Fig. 1h, Supplementary Fig. 1c). To further verify conformational differences between WT-syn fibrils and V40G multimers, we performed a proteinase K (PK) digestion assay. When exposed to PK, higher-order assemblies of proteins have different degrees of accessibility depending on their conformations. Therefore, differences in PK digestion patterns reflect differences in conformations. When aged WT-syn and V40G were partially digested with PK, these proteins produced different fragmentation patterns (Fig. 1i), which indicates that these aggregates have different conformations. We also compared the percentage of digestion and found that V40G multimers were more resistant to PK digestion than WT-syn fibrils (Fig. 1i). This might indicate that V40G multimers are more compact than WT-syn fibrils. However, this needs further validation. Collectively, these results suggest that V40G generates PK-resistant multimers with conformational properties different from those of WT-syn fibrils.

To examine the seeding activity of human WT-syn fibrils and V40G multimers, we performed the following experiments. First, protein misfolding cyclic amplification (PMCA), a method that amplifies fibrils through the seeding mechanism, resulted in much better amplification of WT-syn fibrils than of V40G multimers, suggesting that V40G multimers are much less amplifiable through templated seeding than WT fibrils (Fig. 1j, k). Next, we mixed human WT-syn monomers with 5% (w/w) WT-syn fibrils or V40G multimers, and fibrillation was assayed using Thio T fluorescence. The addition of WT-syn fibrils eliminated the lag time to fibrillation, clearly demonstrating its seeding effect (Fig. 1l). In contrast, the addition of V40G multimers completely inhibited fibrillation of the WT monomer (Fig. 1l). These seeding samples were also analyzed by western blotting, and the results showed that WT fibril seeds accelerated, while V40G multimers inhibited, the aggregation of monomers (Fig. 1m). To verify cross-seeding between α-synuclein proteins from different species, we then performed seeding assays with mouse WT-syn monomers. Both human WT-syn and mouse WT-syn fibrils successfully seeded the fibrillation of mouse WT-syn monomers (Fig. 1n, o). In contrast, human V40G multimers delayed the fibrillation of the mouse WT-syn monomer (Fig. 1n, o). Therefore, human WT-syn can seed the fibrillation of mouse WT-syn, while human V40G multimers inhibit the aggregation of mouse WT-syn. These results suggest that the V40G variant produces PK-resistant forms of multimers that can block fibril formation in the WT-syn protein in vitro.

### V40G injection causes robust synucleinopathy in mice

**Fig. 2:**
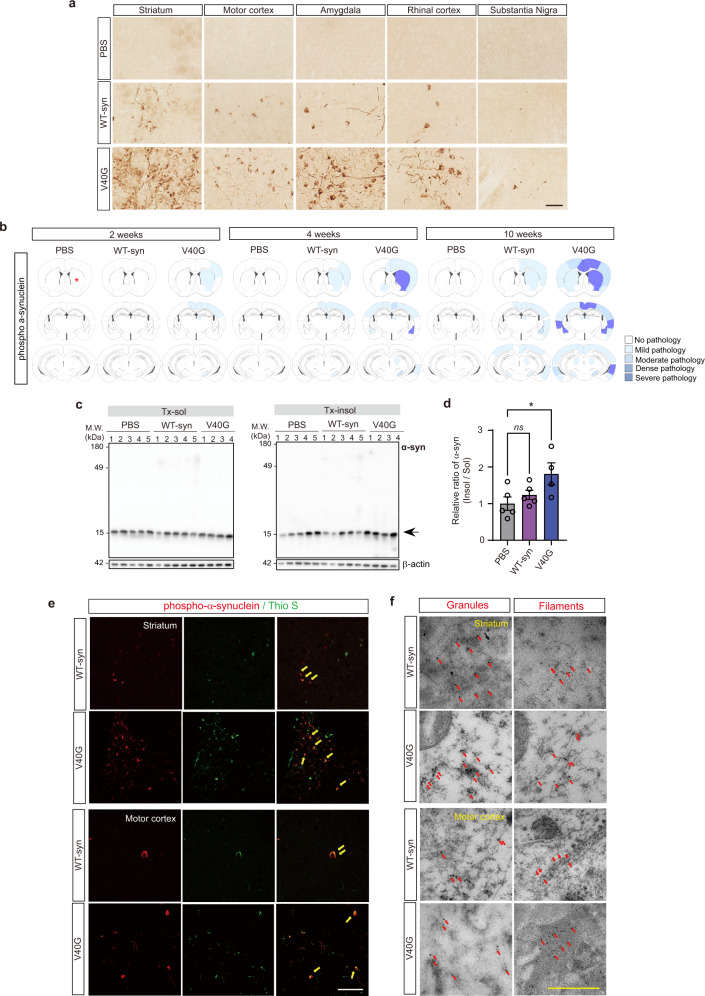
Spreading of synucleinopathy after intracerebral injections of the WT-syn fibril and V40G multimer α-synuclein. **a** Representative images of mouse brain Section 10 weeks after injection of PBS, WT-syn fibrils and V40G multimers stained with phospho-α-synuclein (pS129). Scale bar, 50 μm. **b** Heatmap of regions affected by α-synuclein pathology at 2, 4, and 10 weeks after seed injection (asterisks indicate the injection site; *n* = 6 per group). **c, d** Western blotting of brain tissue extracts obtained 10 weeks after injection. The ratio of insoluble to soluble Tx-100 is quantified in (**d**). Data are expressed as the mean ± SEM, one-way ANOVA with Tukey’s post hoc test, two-sided, *P < 0.05. **e** Co-immunofluorescence images. Thioflavin-positive pS129 aggregates are indicated with arrows. t = 10 weeks. Scale bar, 100 μm. **f** Immunoelectron microscopy with a pS129 antibody. pS129-positive aggregates with granular and filamentous structures are indicated with arrows. t = 10 weeks. Scale bar, 0.5 μm.

To examine the abilities of WT-syn fibrils and V40G multimers to induce aggregate spreading in the brains of animals, we sonicated (Supplementary Fig. 1d) and injected these proteins or vehicle (phosphate buffered saline; PBS) into the striatum of naïve C57BL/6 mice and examined the neuropathology 2, 4 and 10 weeks after injection (Fig. 2). Surgery itself did not cause blood cell infiltration or serum extravasation (Supplementary Fig. 2a–c). The injected proteins were rapidly cleared from the brain. WT-α-syn fibrils were more stable than V40G multimers, with half-lives of 0.67 days vs. 0.45 days for detergent-soluble proteins and 4.49 days vs. 0.43 days for detergent-insoluble proteins (Supplementary Fig. 3a–f). Spreading of phospho-α-synuclein (pS129), a marker of fibrillar aggregates in synucleinopathy, was apparent in several brain regions 10 weeks after injection of WT-syn fibrils (Fig. 2a, b). Unexpectedly, injection of V40G multimers, which blocked seeding in the in vitro assays, resulted in more robust pS129 signals throughout the brain than did WT-syn fibril injection (Fig. 2a, b, Supplementary Fig. 4a–e, Supplementary Table 1). Western analysis also showed that detergent-insoluble α-synuclein was increased after the injection of V40G multimers (Fig. 2c, d). The pS129-positive aggregates were colabeled with thioflavin S, suggesting that these aggregates were β-sheet-rich (Fig. 2e). Filamentous structures of these aggregates were validated with immuno-EM with an α-synuclein antibody (Fig. 2f). Some immune-positive aggregates in the brain also showed granular structures (Fig. 2f). These results suggest that templated seeding is not necessary for driving aggregate spreading and, thus, is not the only mechanism.

### The early phases of aggregate spreading are characterized by a strong inflammatory response that correlates with the levels of aggregation

**Fig. 3:**
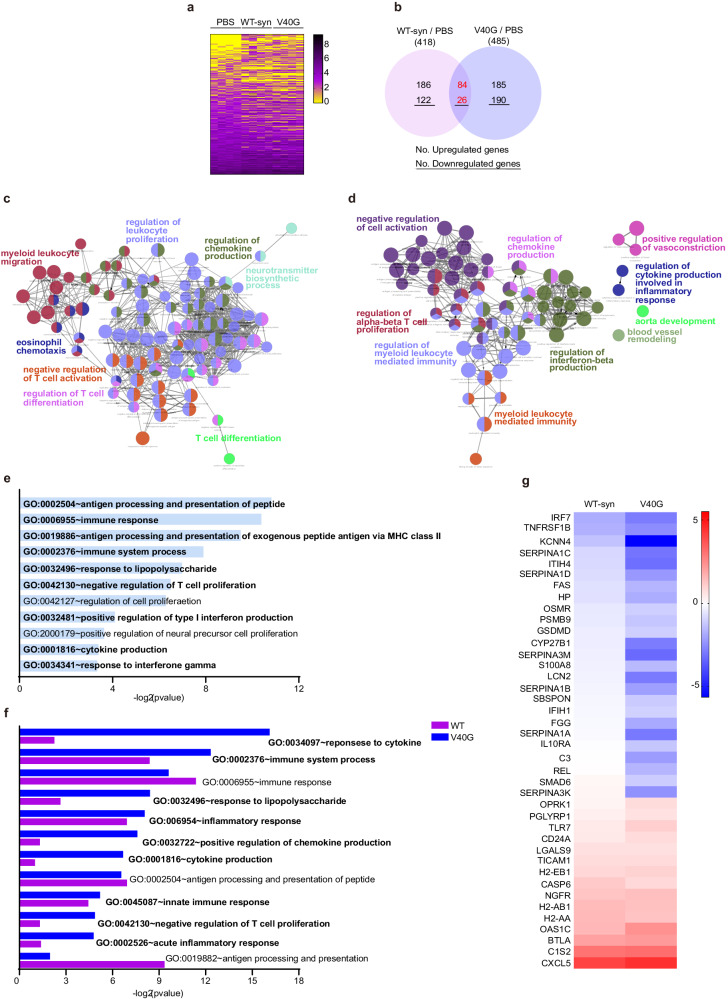
Differential gene expression related to the inflammatory response by injection of α-synuclein. **a** Heatmap representing the expression levels (log2 read count number) of DEGs upregulated (fold change >1.5) or downregulated (fold change <0.5) after injection of WT-syn fibrils or V40G multimers vs. PBS (*n* = 3 per group). **b** Venn diagram of DEGs. **c, d** Simplified networks of significantly enriched GO terms. The network was made from DEGs in WT-Syn-injected mice (**c**) and V40G-injected mi**c**e (**d**). Each term is statistically significant (Benjamini‒Hochberg correction <0.05). The nodes (colored circles) represent significantly enriched parent GO terms. The edges (lines between the nodes) show that there are overlapping genes within terms. The different sizes of the nodes represent the number of enriched genes. **e** The top 11 enriched GO terms for the 110 common DEGs in both WT-syn- and V40G-injected mice. **f** GO enrichment analysis of immune-related common DEGs in both WT-syn- and V40G-injected mice. **g** Heatmap of the log2 fold changes of 40 common DEGs related to immune and inflammatory responses.

To determine the molecular processes occurring during the early phases of aggregate spreading, we analyzed the transcriptome changes in the rhinal cortex, one of the major initial spreading sites, 4 weeks after an intrastriatal injection of WT-syn fibrils, V40G multimers, or vehicle (PBS). The heatmap (Supplementary Fig. 5a, b), which shows the Euclidean distances between the samples, demonstrates that the gene expression patterns in V40G-injected mice were more similar to those in WT-syn-injected mice than to those in vehicle-injected mice. We filtered the genes by the magnitude of their difference in expression (fold change >0.5) and p values (<0.05) (Supplementary Fig. 5c, d, Supplementary Table 2) to identify a total of 418 differentially expressed genes (DEGs) in WT-syn-injected mice and 485 in V40G-injected mice compared to expression in vehicle-injected mice (Fig. 3a, b, Supplementary Table 2). V40G-injected mice showed more robust induction of these DEGs than WT-syn-injected mice (Supplementary Fig. 5e). An enrichment map based on Gene Ontology (GO) terms showed that the immune system process was the key enriched term in both groups (Fig. 3c, d). The DEGs in WT-syn-injected mice were mostly related to immune responses, such as leukocyte proliferation and migration, chemokine production, T-cell activation, antigen processing and presentation, and inflammatory responses (Fig. 3c, Supplementary Fig. 5f). Consistent with the enriched GO analysis, enriched Kyoto Encyclopedia of Genes and Genomes (KEGG) pathway analysis of DEGs in WT-syn-injected mice identified mostly immune-related pathways, e.g., antigen processing and presentation, Toll-like receptor signaling, cytokine–cytokine receptor interaction, and complement and coagulation cascades (Supplementary Fig. 5g). GO analysis of DEGs from V40G-injected mice also showed changes in immune responses, including leukocyte-mediated immunity, T-cell activation, interferon beta production, chemokine production, cytokine production, cytokine responses, immune system processes, and inflammatory responses (Fig. 3d, Supplementary Fig. 5h). Likewise, three immune response-related KEGG pathways—the tumor necrosis factor (TNF) signaling pathway, complement and coagulation cascades, and cytokine–cytokine receptor interactions—were enriched in the same DEGs (Supplementary Fig. 5i). Among these DEGs, 110 were common between WT-syn- and V40G-injected mice (Fig. 3b, Supplementary Table 3). We analyzed the enriched GO pathways to which the 110 common DEGs belonged, among which 84 genes were upregulated and 26 were downregulated (Fig. 3b, Supplementary Table 3). GO analysis of these common DEGs revealed immune-related pathways almost exclusively (Fig. 3e). Next, we compared the extent of immune-related GO enrichment between the WT-syn- and V40G-injected mice, with lower p values indicating more meaningful enrichment in the GO pathway. The majority of immune-related GO pathways were more significantly enriched in V40G-injected mice than in WT-syn-injected mice (Fig. 3f), suggesting that immune/inflammatory responses occur early in α-synuclein aggregate-injected mouse brains and that V40G multimers cause more robust immune-related changes than WT-syn fibrils. Consistent with this suggestion, when 40 immune-related DEGs were selected and their fold changes were compared, the majority of genes showed a greater fold change in V40G-injected mice than in WT-syn-injected mice (Fig. 3g, Supplementary Fig. 5e).

**Fig. 4:**
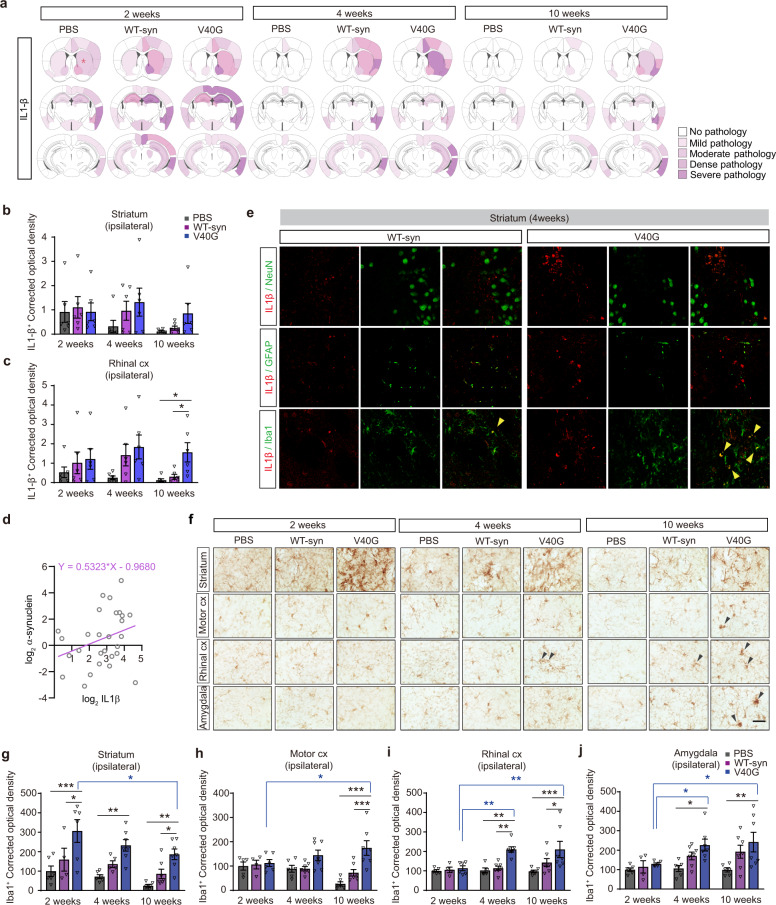
The inflammatory response strongly modulates α-synuclein spreading. **a** Heatmap of regions affected by IL-1β pathology at 2, 4, and 10 weeks after seed injection (asterisks indicate the injection site) (*n* = 6 per group). **b, c** Optical density of areas covered by IL-1β in the striatum (**b**) and rhinal cortex (**c**). **d** Correlations between IL-1β and phospho-α-synuclein (pS129) immunoreactivities in the brain region. Pearson’s correlation coefficient = 0.307822. **e** Co-immunofluorescence images of the striatum. IL-1β was produced in Iba-1-positive microglia but not in neurons or astrocytes. t = 4 weeks. Scale bar, 10 μm. **f** Representative images of regions in which α-synuclein propagation had been observed, showing reactive microglia; tissue stained with anti-Iba-1 antibody (microgliosis marker). Scale bar, 20 μm. **g-j** Optical density of areas covered by Iba-1 immunoreactivity. **g** Striatum (PBS: *n* = 6, 6, 6 at 2, 4, and 10 weeks, respectively; WT-syn: *n* = 4, 5, 7; V40G: *n* = 6, 7, 7). **h** Motor cortex (PBS: *n* = 5, 6, 6; WT-syn: *n* = 4, 7, 7; V40G: *n* = 6, 7, 7). **i** Rhinal cortex (PBS: *n* = 5, 5, 6; WT-syn: *n* = 4, 7, 7; V40G: *n* = 6, 7, 7). **j** Amygdala (PBS: *n* = 6, 6, 6; WT-syn: *n* = 4, 7, 7; V40G: *n* = 6, 7, 7). Data are expressed as the mean ± SEM, one-way ANOVA with Tukey’s post hoc test, two-sided, *P < 0.05, **P < 0.01, ***P < 0.0001.

To validate the inflammatory responses experimentally, we performed immunohistochemical analysis of interleukin (IL)-1β as an inflammatory marker in the same group of animals described in Fig. 4. IL-1β immunoreactivity was found in many regions throughout the brain 2 weeks after injection in all groups, including PBS-injected animals (Fig. 4a). However, this inflammatory marker disappeared rapidly in PBS-injected brains, whereas it remained present in brains injected with either WT-syn or V40G until at least 10 weeks after injection. V40G injection resulted in more sustained and robust IL-1β immunoreactivity than WT-syn injection (Fig. 4a–c, Supplementary Table 4), and the immunoreactivity correlated well with the extent of α-synuclein accumulation (Fig. 4d). Coimmunostaining of IL-1β with cellular markers, such as NeuN, GFAP, and Iba-1, showed that IL-1β expression occurred exclusively in Iba-1-positive microglia (Fig. 4e). We then analyzed the extent of microgliosis with Iba-1 immunohistochemistry and found that microgliosis was present in animals injected with either WT-syn fibrils or V40G multimers but was more extensive and sustained after V40G injection (Fig. 4f–j). In contrast to microgliosis, the extent of astrogliosis did not correlate well with pS129 spreading (Supplementary Fig. 6a–e).

**Fig. 5:**
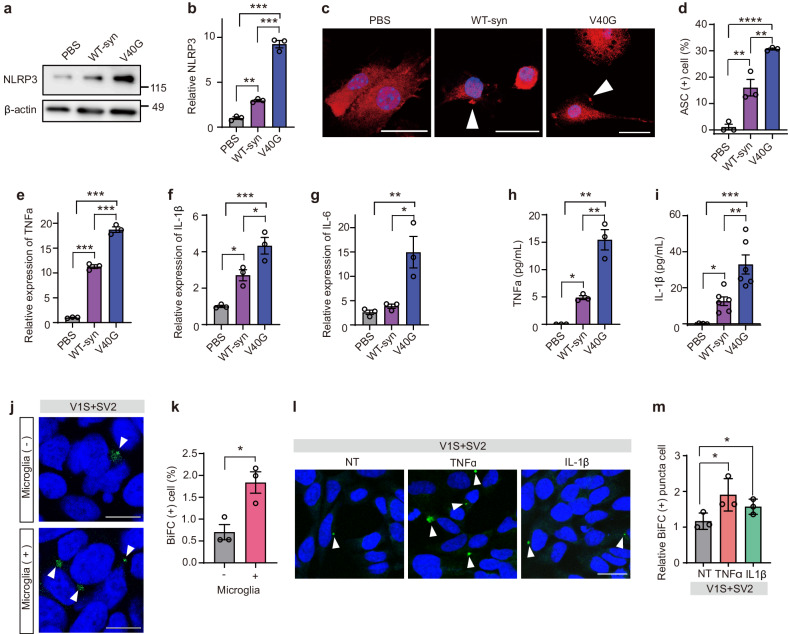
Microglial activation promotes α-synuclein propagation. **a, b** Induction of NLRP3 in microglia. The NLRP3 intensity was normalized to the value of β-actin (*n* = 3). **c** ASC speck formation in microglia. Arrowheads indicate ASC specks. Scale bar: 20 μm. **d** Quantification of ASC speck-positive cells (*n* = 3, 100 cells per experiment). **e-g** Relative expression of inflammatory cytokines. TNF-α (**e**), IL-1β (**f**), and IL-6 (**g**) in microglia. Quantitative PCR data were normalized to the average value of those in the PBS-treated group (*n* = 3). **h, i** Secretion of inflammatory cytokines in microglia. The amounts of secreted TNF-α (**h**) and IL-1β (**i**) were quantified by ELISA. *n* = 3 in **h**, *n* = 6 in **i**. **j, k** Effects of microglia on cell-to-cell propagation of α-synuclein. **j** BiFC-positive cells are indicated with arrowheads. Scale bar: 20 µm. **k** Quantification of BiFC-positive cells (*n* = 3, 200 cells per experiment). **l, m** Effects of TNF-α and IL-1β treatment on cell-to-cell propagation of α-synuclein. V1S and SV2 cells were cocultured and then treated with either TNF-α or IL-1β (50 ng/ml) for 24 h. **l** BiFC-positive cells are indicated with arrowheads. Scale bar, 20 µm. **m** Quantification of BiFC-positive cells (*n* = 3, 300 cells per experiment). Statistical significance was determined by one-way ANOVA with Tukey’s post hoc comparison between groups (**b, d-i**) or by a two-tailed unpaired Student’s *t* test (**k**, **m**), *P < 0.05, **P < 0.01, ***P < 0.0001. All data are presented as the mean ± SEM.

To verify the inflammatory responses of microglia after exposure to WT-α-syn fibrils and V40G multimers, we analyzed inflammasome activation and cytokine production in cultured primary microglia. Both WT-α-syn fibrils and V40G multimers activated inflammasomes, showing induction of NLRP3 (Fig. 5a, b) and ASC speck formation (Fig. 5c, d). NLRP3 induction was significantly higher with V40G-α-syn treatment than with WT-α-syn treatment (Fig. 5a–d). The production of cytokines, such as TNF-α, IL-1β, and IL-6, was stronger after V40G multimer treatment than after WT-α-syn fibril treatment (Fig. 5e–i). The stronger responses for V40G multimers than for WT-α-syn fibrils were not attributed to the stability of the proteins; WT-α-syn fibrils were more stable than V40G multimers in microglia (Supplementary Fig. 3g–j). These results confirmed the transcriptome analysis and indicate that inflammatory responses precede aggregate spreading and that the extent of inflammation correlates with that of α-synuclein aggregation.

To directly test the effects of microglia and inflammatory cytokines on the propagation of α-synuclein, we used the dual-cell BiFC system, which is composed of two cell lines, V1S and SV2^26^. In this system, when α-synuclein is transferred from cell to cell, yellow fluorescent puncta appear in the cellular cytoplasm, allowing quantitative assessment of α-synuclein propagation. By measuring the percentage of cells with fluorescent puncta, we found that both coculturing with microglia and exposing V1S and SV2 cell lines to TNF-α and IL-1β significantly increased the cell-to-cell propagation of α-synuclein (Fig. 5j–m).

### Aspirin reversed V40G-induced behavioral deficits and pathology

**Fig. 6:**
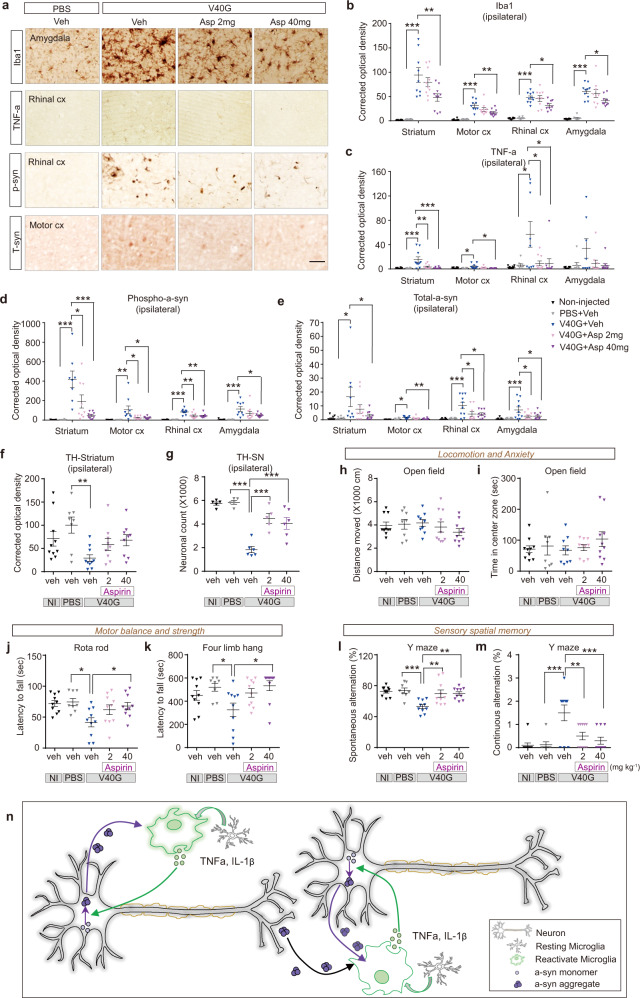
Suppression of synucleinopathy, dopaminergic terminal loss and motor functions by an anti-inflammatory drug. **a** Representative images of mouse brain sections after injection of V40G multimers followed by 17 weeks of oral aspirin (ASP) administration and staining for Iba-1, TNF-α, pS129 and total α-synuclein. Scale bar, 50 μm. **b-e** Optical density of several brain areas covered by Iba-1, TNF-α, pS129 and total α-synuclein immunoreactivity (n = 8–10). **f** Optical density of the ipsilateral striatal region covered by TH immunoreactivity. (Noninjected (NI), *n* = 10; PBS, *n* = 8; V40G, *n* = 10; V40G + ASP 2, *n* = 9; V40G + ASP 40, *n* = 9). **g** Stereological cell counts of TH-immunoreactive dopaminergic neurons in the ipsilateral substantia nigra pars compacta of mice (NI, *n* = 5; PBS, *n* = 5; V40G, *n* = 6; V40G + ASP 2, *n* = 5; V40G + ASP 40, *n* = 6). **h, i** Open field test: distance moved (cm) and time in the center (s) were measures of locomotion and anxiety, respectively (NI, *n* = 10; PBS, *n* = 8; V40G, *n* = 9; V40G + ASP 2, *n* = 10; V40G + ASP 40, *n* = 10). **j, k** Rotarod and four-limb hanging tests: the latency to fall (s) in these tests served as measures of motor balance and strength, respectively (NI, *n* = 10; PBS, *n* = 8; V40G, *n* = 10; V40G + ASP 2, *n* = 10; V40G + ASP 40, *n* = 10). **l, m** Y maze: spontaneous alternation (%) and continuous alternation (n) were indicators of sensory spatial memory (NI, *n* = 10; PBS, *n* = 8; V40G, *n* = 10; V40G + ASP 2, *n* = 10; V40G + ASP 40, *n* = 10). Data are expressed as the mean ± SEM, one-way ANOVA with Tukey’s post hoc test, two-sided, *P < 0.05, **P < 0.01, ***P < 0.0001. **n** Graphic depicting the role of microglial inflammation in synucleinopathy propagation. See details in the Discussion.

To confirm the role of inflammation in the pathology of α-synuclein spreading, we administered aspirin, a common anti-inflammatory drug, to C57BL/6 mice for 17 weeks, starting 1 week after intrastriatal injection of WT fibrils, V40G multimers, or PBS, and examined pathological changes and behavioral outcomes (Fig. 6, Supplementary Fig. 7a). Aspirin was delivered daily in the drinking water at approximate doses of 2 mg per kg and 40 mg per kg. When we examined the effects of aspirin administration on inflammation and aggregation at 18 weeks after injection, we found that the V40G-induced increases in the levels of TNF-α and microgliosis were prevented, confirming the anti-inflammatory efficacy of this drug in the brain (Fig. 6a–c). Aspirin also significantly decreased abnormal pS129 deposition and total α-synuclein in the injected animals (Fig. 6a, d and e). Likewise, the loss of dopaminergic terminals in the striatum and dopaminergic cell bodies in the substantia nigra pars compacta were reversed by aspirin treatment (Fig. 6f, g, Supplementary Fig. 7b–d). Injection of WT-α-syn fibrils also caused dopaminergic cell loss, although to a lesser extent than V40G multimers (Supplementary Fig. 7e vs. Fig. 6g). The cell loss caused by WT-α-syn fibrils was also reversed by aspirin administration (Supplementary Fig. 7e). All pathological changes described here, except for the dopaminergic cell count, were observed not only in the injected hemisphere but also in the contralateral hemisphere. These results emphasize the roles of proinflammatory factors in aggregate propagation and ensuing pathology.

To examine the effects of aspirin on behavioral deficits, the mice were subjected to a battery of behavioral assessments at 12 and 18 weeks after injection. The total activity and anxiety-like behavior (measured in the open field test) of V40G-injected mice did not differ from those of either naïve (noninjected) controls or PBS-injected controls. However, V40G-injected mice showed deficits in motor control (rotarod test), motor strength (four-limb hanging test), and sensory spatial memory (Y maze test) (Fig. 6h–m shows data at 18 weeks). The behavioral deficits developed in a time-dependent manner and were less severe at 12 weeks than at 18 weeks after injection (Supplementary Fig. 7a, 8a–g). Aspirin 40 mg per kg completely reversed all the behavioral deficits, and some deficits were even reversed by 2 mg per kg. The normal behavior of noninjected or PBS-injected mice was not affected by aspirin treatment (Supplementary Fig. 8h–k).

## Discussion

Herein, we have identified a sequence variant of α-synuclein (V40G) that forms stable multimers that not only lack seeding ability but also block the fibrillation of WT-α-syn in vitro. In contrast to these in vitro properties, V40G multimers effectively propagated α-synuclein aggregates after intrastriatal injections in mice. These findings suggest that templated seeding is not solely responsible for aggregate spreading. The synucleinopathy induced by injection of either WT-syn or V40G was preceded by inflammatory responses, and the degree of synucleinopathy correlated with the extent of inflammation. Furthermore, V40G-induced synucleinopathy was suppressed by the administration of an anti-inflammatory drug, consistent with an important role of inflammation in synucleinopathy spreading.

Previous studies have shown a relationship between neuroinflammation and protein aggregation. Lipopolysaccharide (LPS)-induced brain inflammation induces α-synuclein aggregation in mice^29^. The present work also showed that the proinflammatory cytokines TNF-α and IL-1β stimulated cell-to-cell propagation of α-synuclein in vitro. Neuron-released oligomeric α-synuclein has been shown to induce inflammatory responses from microglia through the activation of Toll-like receptor 2 (TLR2)^30^. Therefore, as an alternative to the templated seeding model, we propose a model that emphasizes the role of the inflammatory microenvironment in aggregate propagation. In this model, protein aggregates would initially induce chronic inflammation, which in turn would create a microenvironment that would favor protein aggregation in neurons, establishing a vicious cycle between protein aggregation and inflammation. The protein aggregates would then be transferred through anatomical neural connections and would establish another inflammatory microenvironment, expanding the vicious cycle between protein aggregation and inflammation to the new location (Fig. 6n). Consistent with this model, Olanow *et al*. recently reported that brain immune cell activation preceded α-synuclein aggregation in fetal mesencephalic neuronal transplants in patients with Parkinson’s disease^31^. Our work is also in line with recent work in which inflammasomes were shown to play important roles in tau propagation^32^. The incubation time that occurs prior to aggregate spreading in experimental animals may be the period that is required for the cycle between the inflammatory microenvironment and protein aggregation to be established. The coexistence of different pathological structures, e.g., Lewy bodies and neurofibrillary tangles, in many human specimens might also be explained by the inflammatory microenvironment model, in which aggregation of one protein generates a microenvironment that favors not only the aggregation of homotypic aggregates but also that of other aggregation-prone proteins.

Our current experimental system mimics the inflammatory microenvironment that triggers protein aggregation and other pathological changes, and we provide evidence that these phenotypes are led by microglia. These findings are in line with recent observations showing detrimental effects of immune cells and inflammation in synucleinopathy models^31,33–35^. However, the opposite effects of immune cells have also been reported^36–38^. In the latter cases, immune cells adapted to the protective states, in which the scavenging activity was strong while detrimental inflammation was suppressed. It has become evident that the cells involved in brain innate immunity, including microglia and astrocytes, exist in many different states^39–42^. These cells may transform from one state to another, sensing the microenvironment. The mechanism by which these different states are switched is still unknown and remains one of the most important and challenging questions in neurobiology.

The results of this study clearly underscore the key role played by inflammatory processes in α-synuclein spreading. They also raise important new questions and suggest new pathogenic mechanisms underlying the development of α-synuclein pathology. For example, during the development of proteinopathies, different aggregates with distinct conformations might generate different types of inflammatory microenvironments that would favor the formation of aggregates with corresponding structures. Another important question raised by the current study is how inflammation modulates aggregate propagation. One possibility is that soluble inflammatory factors released by glial cells promote the secretion of α-synuclein from neurons. Our recent study showed that TNF-α promoted neuronal senescence and secretion of α-synuclein through a senescence-associated secretory phenotype^43^. Another possibility is that the inflammatory microenvironment modulates neural activity, which in turn regulates the propagation of α-synuclein. Recently, Ueda et al. showed that suppression of neural activity using an antiepileptic drug caused a decreased cell-to-cell propagation of α-synuclein^44^. This is consistent with the findings that secretion and uptake of α-synuclein is influenced by neural activity^45–47^.

How V40G multimers inhibit the fibrillation of WT-syn in vitro is not clearly understood. Multiple biochemical analyses, including CD, dye binding assays, and PK digestion, suggested that V40G multimers possess different conformations from WT-syn fibrils. EM shows that the V40G multimers do not grow into long filaments. Based on these results, we speculate that V40G-α-syn assembles to form alternative conformations that run into the “dead-end” of the assembly process. Interaction of these “dead-end” multimers with WT-syn would interfere with fibril elongation of the latter, acting as “crystal poisons”^48^. Further characterization of the structural properties of V40G-α-syn would provide more information on the precise mechanism of its effects on fibrillation inhibition.

It is difficult to make a one-to-one correlation between the regions of α-synuclein pathology and specific behavioral deficits, since defects in neural functions by α-synuclein deposition cannot be assessed precisely in our experimental settings. Nevertheless, it is worth noting that α-synuclein pathologies were detected in the retrosplenial cortex and the somatosensory cortex at 2 weeks after injection, prior to the development of pathologies in the motor cortex (Supplementary Table 1), implying a more rapid pathological progression in the retrosplenial cortex and the somatosensory cortex than in the motor cortex. Given that V40G-injected mice exhibited sensory spatial memory deficits in the Y maze test at 12 weeks after injection, while motor behavioral deficits were more delayed, the development of behavioral abnormalities appeared to have some correlation with α-synuclein pathology.

In conclusion, our results provide new insights into how protein aggregates propagate and are likely to lead to new research unveiling core principles of disease progression in Parkinson’s disease. Furthermore, as a result of this study, new lines of research will open up to investigate the role of inflammation in the propagation of other proteins whose aggregation is associated with neurodegenerative diseases.

### Supplementary information

Supplementary information

## Data Availability

Raw sequencing reads for the rhinal cortex have been deposited at the National Center for Biotechnology Information under BioProjects PRJNA605306. Other data are available from the corresponding author upon reasonable request.
