# Correction: Inflammation promotes synucleinopathy propagation

**DOI:** 10.1038/s12276-024-01217-y

**Published:** 2024-04-01

**Authors:** Tae-Kyung Kim, Eun-Jin Bae, Byung Chul Jung, Minsun Choi, Soo Jean Shin, Sung Jun Park, Jeong Tae Kim, Min Kyo Jung, Ayse Ulusoy, Mi-Young Song, Jun Sung Lee, He-Jin Lee, Donato A. Di Monte, Seung-Jae Lee

**Affiliations:** 1 Department of Biomedical Sciences, Seoul National University College of Medicine, Seoul, 03080 Korea; 2 Department of Exercise Physiology and Sport Science Institute, Korea National Sport University, Seoul, 05541 Korea; 3 Neuroscience Research Institute, Seoul National University College of Medicine, Seoul, South Korea; 4 Neural Circuits Research Group, Korea Brain Research Institute, Daegu, 41068 Korea; 5 German Center for Neurodegenerative Diseases (DZNE), Bonn, Germany; 6 Department of Biomedical Science and Technology, Konkuk University, Seoul, 143-701 Korea; 7 Department of Anatomy, Konkuk University, Seoul, 05029 Korea; 8 IBST, Konkuk University, Seoul, 05029 Korea; 9 SNU Dementia Research Center, Seoul National University College of Medicine, Seoul, South Korea; 10 Neuramedy Co. Ltd., Seoul, South Korea; 11 Present Address: Nutritional Sciences and Toxicology Department, University of California Berkeley, Berkeley, CA 94720 USA; 12 Present Address: IPS Intellectual Property Law Firm, Seoul, Korea; 13 Present Address: Neuramedy Co. Ltd., Seoul, South Korea

**Keywords:** Parkinson's disease, Parkinson's disease

Correction to: *Experimental & Molecular Medicine* 10.1038/s12276-022-00895-w, published online 6 December 2022

After online publication of this article, the authors noticed an error in the Results section. In the original article “Inflammation promotes synucleinopathy propagation”, there was an error in Fig. 5l. The authors regret to notice that representative images of Fig.5l were inadvertently duplicated during figure assembly from Fig. 1d of Exp Mol Med 10.1038/s12276-022-00789-x. The authors have provided new versions of Fig. 5. The correction will not affect the results and scientific conclusions of the article.
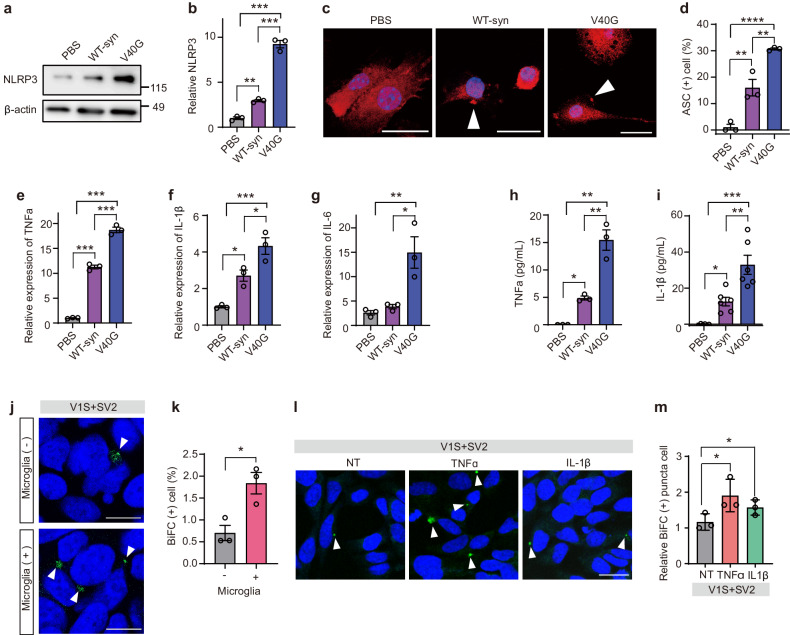


The authors apologize for any inconvenience caused.

The original article has been corrected.

