# Supplementary material for: Inflammation promotes synucleinopathy propagation
Source: Exp Mol Med. 2022 Dec 6;54(12):2148–61. doi: 10.1038/s12276-022-00895-w (PMC9794777; doi:10.1038/s12276-022-00895-w)
Supplement: Supplementary file 1 — Supplementary information [file 12276_2022_895_MOESM1_ESM.pdf]

# Inflammation promotes synucleinopathy propagation

Tae-Kyung Kim [1,10 #], Eun-Jin Bae [1, 2, #], Byung Chul Jung [1, #], Minsun Choi [1], Soo Jean Shin [1], Sung Jun Park [1], Jeong Tae Kim [1], Min Kyo Jung [3], Ayse Ulusoy [4], Mi-Young Song [5], Jun Sung Lee [1], He-Jin Lee [6, 7], Donato A. Di Monte [4] and Seung-Jae Lee [1,2, 8, 9*]

[1] Department of Biomedical Sciences, Seoul National University College of Medicine, Seoul 03080, Korea

[2] Neuroscience Research Institute, Seoul National University College of Medicine, Seoul, South Korea

[3] Neural Circuits Research Group, Korea Brain Research Institute, Daegu 41068, Korea

[4] German Center for Neurodegenerative Diseases (DZNE), Bonn, Germany

[5] Department of Biomedical Science and Technology, Konkuk University, Seoul 143-701, Korea

[6] Department of Anatomy, Konkuk University, Seoul 05029, Korea

[7] IBST, Konkuk University, Seoul 05029, Korea

[8] SNU Dementia Research Center, Seoul National University College of Medicine, Seoul, South Korea

[9] Neuramedy, Seoul, South Korea

[10] Department of Exercise Physiology and Sport Science Institute, Korea National Sport University, Seoul 05541, Korea

*Corresponding author: Department of Biomedical Sciences, Seoul National University College of Medicine, 103 Daehak-ro, Jongro-gu, Seoul 03080, Korea; Tel: +82-2-3668-7037; Fax: +82-2-447-5683; Email: sjlee66@snu.ac.kr

[#]These authors contributed equally to this work.

Present Addresses: Byung Chul Jung, Nutritional Sciences and Toxicology Department, University of California Berkeley, Berkeley, CA 94720; Mi-Young Song, IPS Intellectual Property Law Firm, Seoul, Korea; Jun Sung Lee, Neuramedy Co. Ltd, Seoul, Korea

**Running title: Inflammation and aggregate propagation**

**Supplementary information. Supplementary Figures, legends, and Supplementary tables**

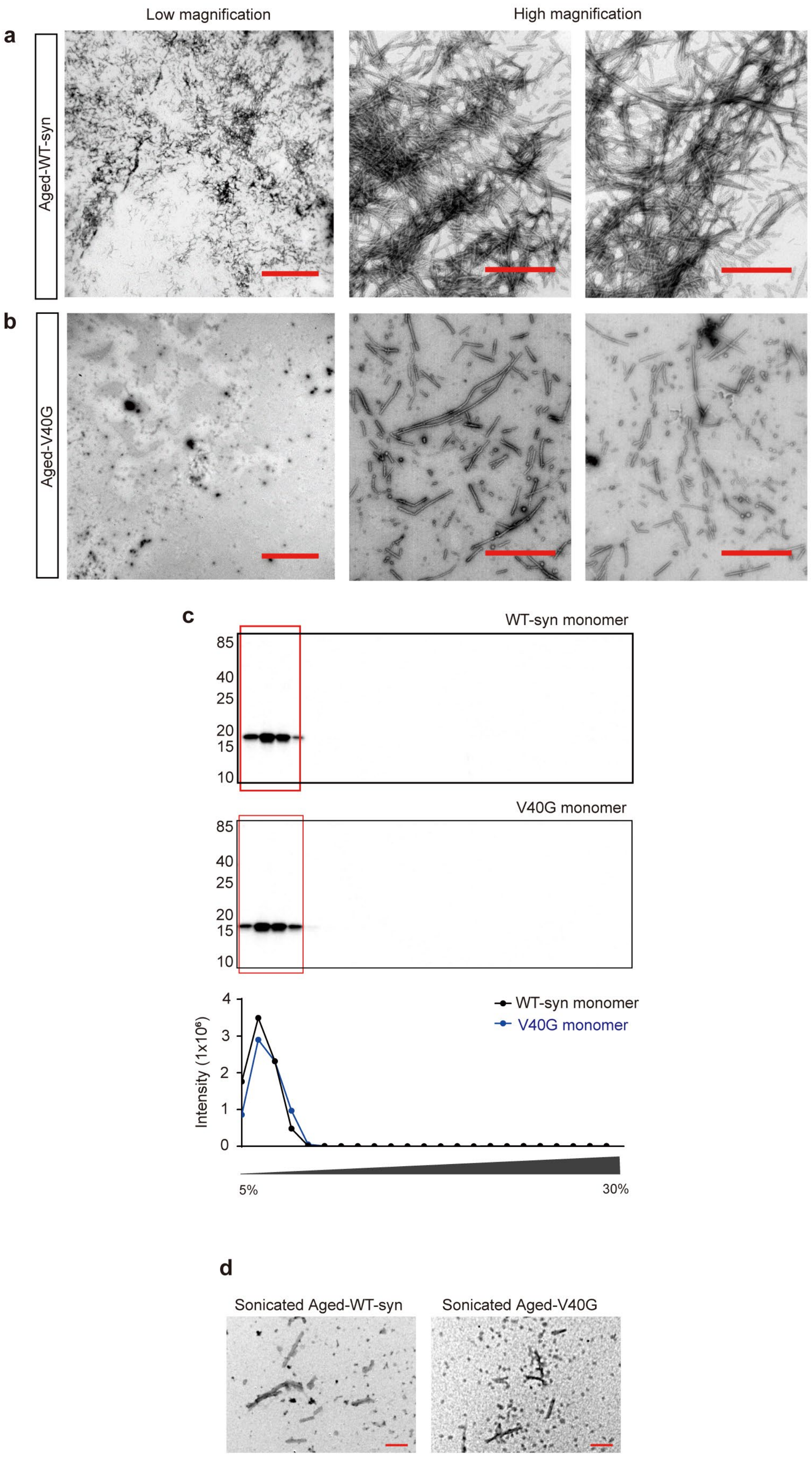
Low magnification
High magnification
a
Aged-WT-syn
b
Aged-V40G
c
WT-syn monomer
85
40
25
20
15
10
V40G monomer
85
40
25
20
15
10
Intensity (1x10⁶)
4
3
2
1
0
WT-syn monomer
V40G monomer
5%
30%
d
Sonicated Aged-WT-syn
Sonicated Aged-V40G

**Supplementary Fig. 1. Characterization of WT-syn and V40G. a, b** TEM image of aged WT-syn (**a**) and aged V40G (**b**). Scale bar, low magnification, 5 μm; high magnification, 0.8 μm. **c** Velocity ultracentrifugation of WT-syn and V40G monomers. Western blotting of fractions (top). Quantification of western results (bottom). **d** TEM image of aged WT-syn and aged V40G after sonication. Scale bar: 200 nm.

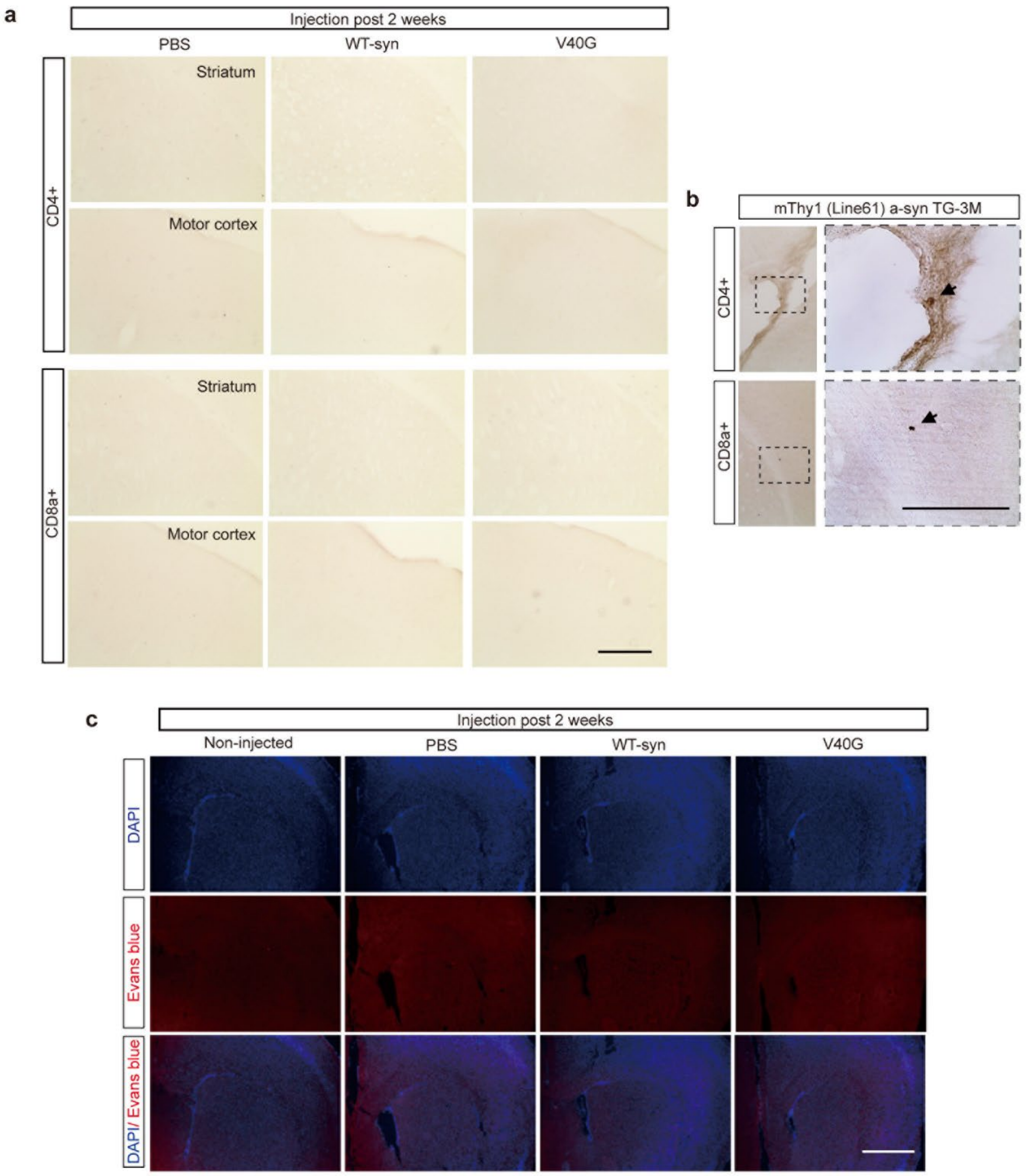


**Supplementary Fig. 2. Assessment of BBB impairment after intrastriatal injections. a** Representative images of the striatal regions 2 weeks after injection. Sections were stained with CD4 and CD8a antibodies. Scale bar, 200 μm. **b** Representative images of 3-month-old mThy1 α-synuclein transgenic mice stained with CD4 and CD8a antibodies. Scale bar, 200 μm. **c** Representative images of the striatal regions 2 weeks after injection stained with Evans blue (marker for extravasated albumin) and DAPI. Scale bar, 1 mm.

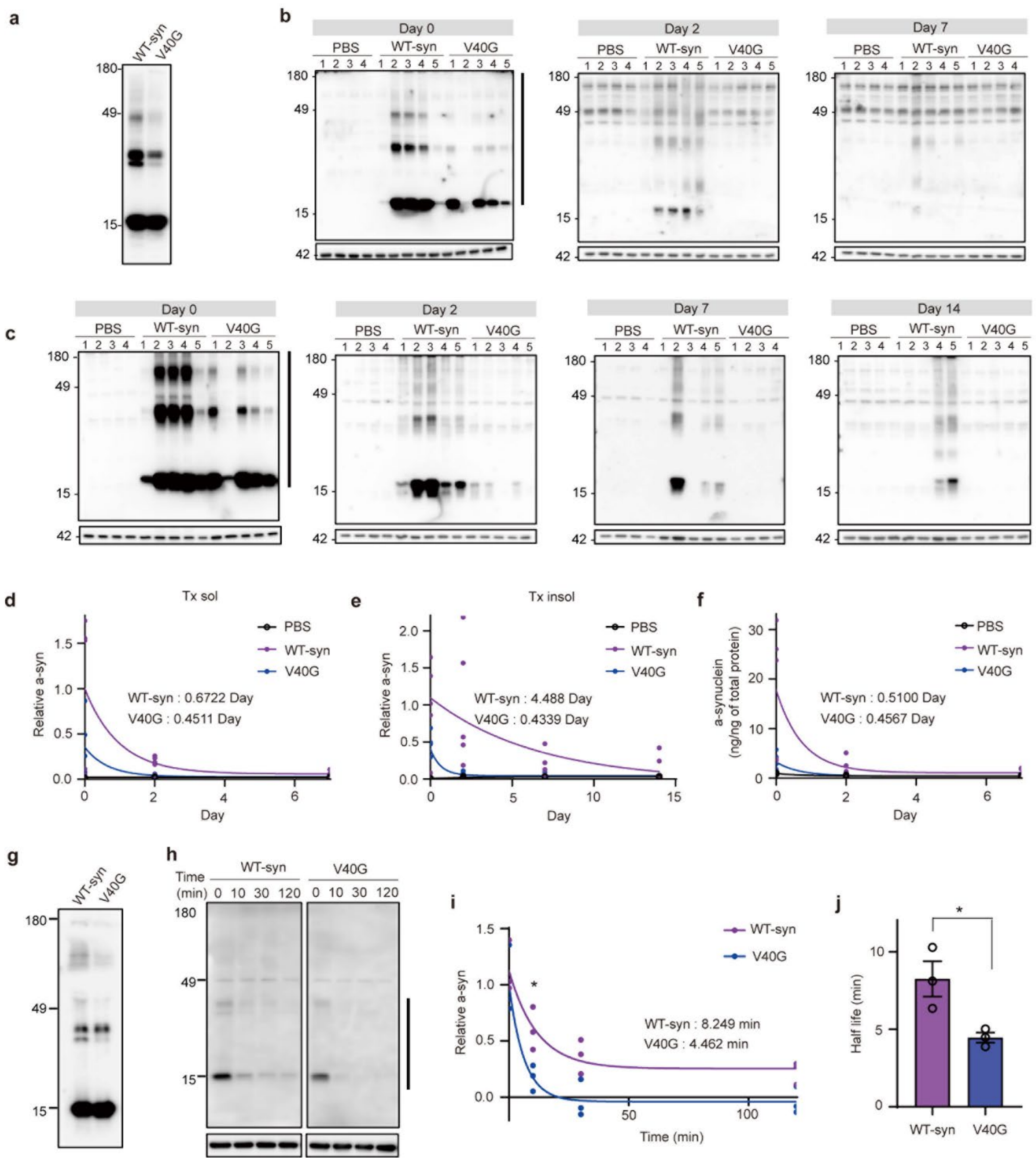


**Supplementary Fig. 3. Stability of aged WT-syn and V40G. a** Western blotting of the injectants. **b, d** Western blotting and clearance kinetics of aged WT-syn and V40G in the Tx-sol fractions. **c, e** Western blotting and clearance kinetics of aged WT-syn and V40G in the Tx- insol fractions. **f** Degradation kinetics of aged WT-syn and V40G syn in Tx-sol fraction by ELISA. **g-j** Mouse primary microglia cells were treated with 200 nM of aged WT-syn or V40G. After a 30-min incubation, the cells were washed and chased for degradation kinetics of the imported proteins. **g** Western blotting of aged WT-syn and V40G used in the microglia

experiments. **h, i** Degradation kinetics of internalized aged WT-syn and V40G in the Tx-sol fractions. Quantified region was indicated on the right as a bar in (H). Relative levels of α-synuclein in (**h**) were quantified in (**i**). *n*=3. Significance was assessed by two-way ANOVA with Bonferroni's post hoc test, two-sided. **j** Half-life of internalized α-synuclein. *n*=3. Significance was assessed by a two-tailed paired t-test, *$P$<0.05. Data are expressed as the mean ± SEM.

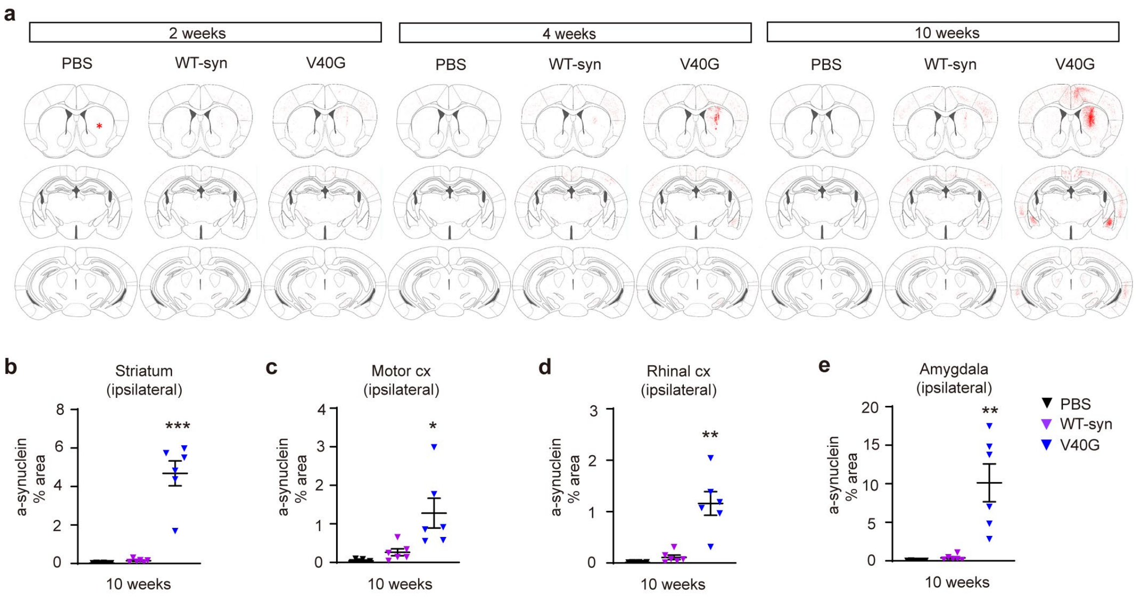


**Supplementary Fig. 4. Maps of α-synuclein pathology. a** Brain distribution of phospho-α-synuclein accumulations at 2, 4 and 10 weeks after PBS, WT-syn fibril and V40G multimer injection (red dots and stippling, respectively; asterisks indicat e the injection site; *n*=6 per group). **b-e** Percentage of brain areas (ipsilateral to the injection side) covered by phospho-α-synuclein (pS129) immunoreactivity 10 weeks after injection with PBS, WT-syn fibrils and V40G multimers in the Striatum (**b**), the motor cortex (**c**), the rhinal cortex (**d**) , and the amygdala (**e**). Data are expressed as the mean ± SEM, two-tailed paired t-test, **P*<0.05, ***P*<0.01, ****P*<0.0001.

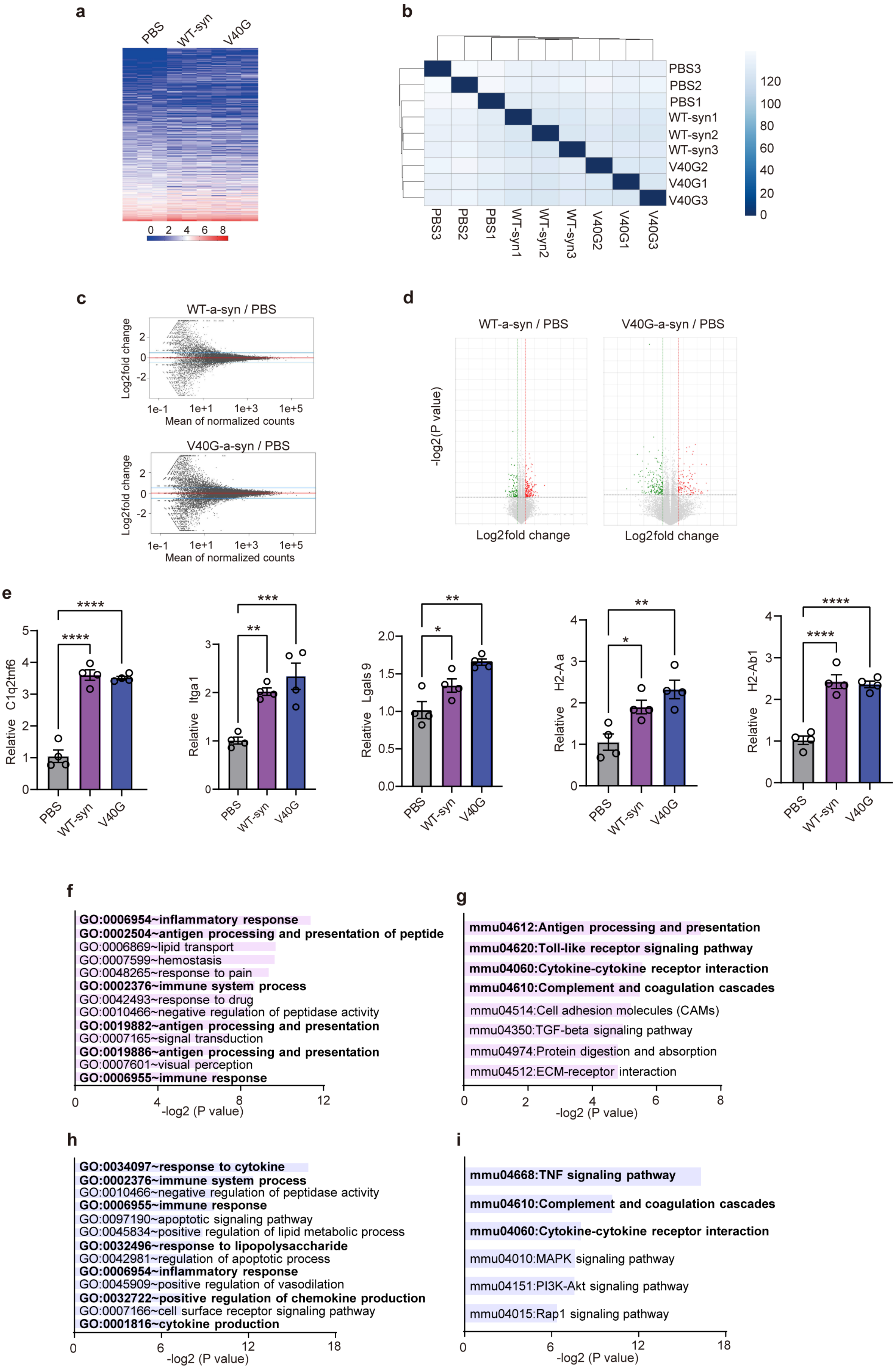

a
PBS
WT-syn
V40G
0 2 4 6 8
b
PBS3
PBS2
PBS1
WT-syn1
WT-syn2
WT-syn3
V40G2
V40G1
V40G3
120
100
80
60
40
20
0
c
WT-a-syn / PBS
V40G-a-syn / PBS
Log2fold change
Mean of normalized counts
1e-1 1e+1 1e+3 1e+5
d
WT-a-syn / PBS
V40G-a-syn / PBS
-log2(P value)
Log2fold change
e
Relative C1q2tnf6
Relative Itga1
Relative Lgals9
Relative H2-Aa
Relative H2-Ab1
PBS
WT-syn
V40G
****
***
**
*
f
GO:0006954~inflammatory response
GO:0002504~antigen processing and presentation of peptide
GO:0006869~lipid transport
GO:0007599~hemostasis
GO:0048265~response to pain
GO:0002376~immune system process
GO:0042493~response to drug
GO:0010466~negative regulation of peptidase activity
GO:0019882~antigen processing and presentation
GO:0007165~signal transduction
GO:0019886~antigen processing and presentation
GO:0007601~visual perception
GO:0006955~immune response
-log2 (P value)
g
mmu04612:Antigen processing and presentation
mmu04620:Toll-like receptor signaling pathway
mmu04060:Cytokine-cytokine receptor interaction
mmu04610:Complement and coagulation cascades
mmu04514:Cell adhesion molecules (CAMs)
mmu04350:TGF-beta signaling pathway
mmu04974:Protein digestion and absorption
mmu04512:ECM-receptor interaction
-log2 (P value)
h
GO:0034097~response to cytokine
GO:0002376~immune system process
GO:0010466~negative regulation of peptidase activity
GO:0006955~immune response
GO:0097190~apoptotic signaling pathway
GO:0045834~positive regulation of lipid metabolic process
GO:0032496~response to lipopolysaccharide
GO:0042981~regulation of apoptotic process
GO:0006954~inflammatory response
GO:0045909~positive regulation of vasodilation
GO:0032722~positive regulation of chemokine production
GO:0007166~cell surface receptor signaling pathway
GO:0001816~cytokine production
-log2 (P value)
i
mmu04668:TNF signaling pathway
mmu04610:Complement and coagulation cascades
mmu04060:Cytokine-cytokine receptor interaction
mmu04010:MAPK signaling pathway
mmu04151:PI3K-Akt signaling pathway
mmu04015:Rap1 signaling pathway
-log2 (P value)

**Supplementary Fig. 5. Overview of DEGs after injection of WT-syn fibrils or V40G multimers.** **a** Heat map representing the expression levels (log2 read count) of all mapped genes in the rhinal cortex from PBS-, WT-syn, and V40G-injected mice. **b** Heat map representing the Euclidean distances between the samples. Distances were analyzed by logarithmic transformation in DESeq2. **c** Mean Average (MA) plots of WT-syn *vs.* PBS and V40G *vs.* PBS. The log 2 foldchange for each comparison is shown on the y-axis and the average counts normalized by size factor is plotted on the x-axis. **d** Volcano plots of WT-syn *vs.* PBS and V40G *vs.* PBS. DEGs were selected by their fold changes (>0.5) and p values (<0.05). Green dots represent downregulated DEGs, and red dots indicate upregulated DEGs. **e** Relative expression level of representative DEGs by RT-qpcr. $n$=3. Data are expressed as the mean ± SEM. Significance was assessed one-way ANOVA with Dunnett's post hoc test, *$P$<0.05, **$P$<0.01, ****$P$<0.00001. **f, g** Enriched GO (**f**) and KEGG (**g**) analyses for 418 DEGs in WT-syn-injected mice compared with PBS-injected mice. **h, i** Enriched GO (**h**) and KEGG (**i**) analyses for 485 DEGs in V40G-injected mice compared with PBS-injected mice.

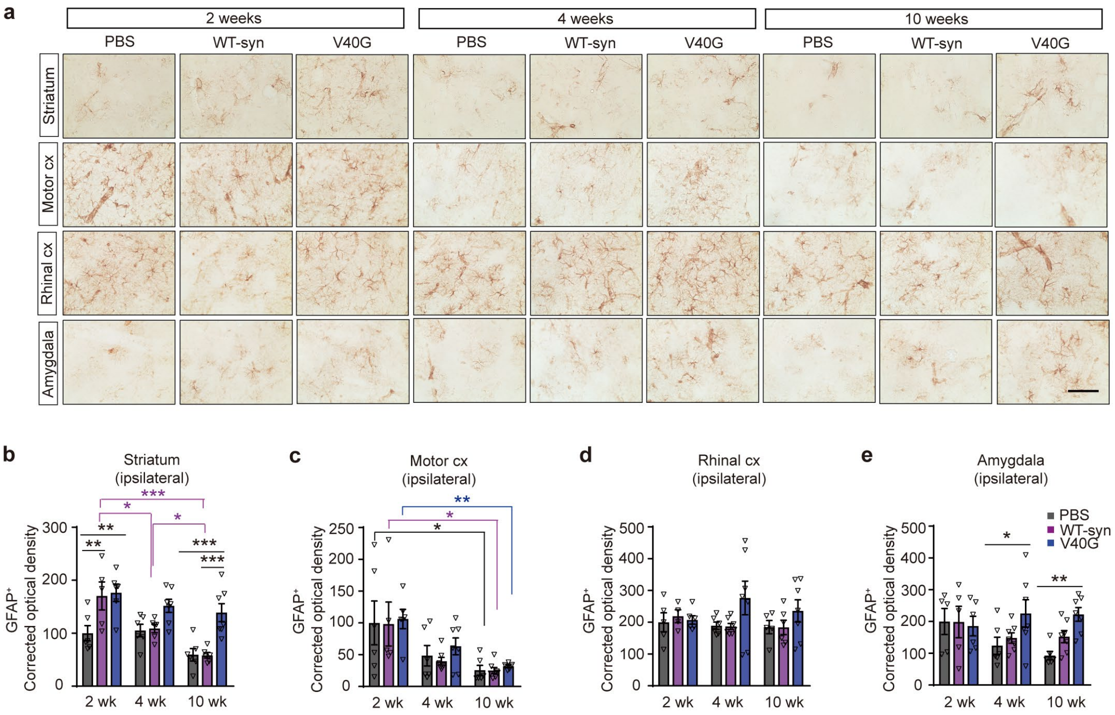


**Supplementary Fig. 6. Representative images of astroglial activation. a** Representative images of α-synuclein propagation regions 2, 4, and 10 weeks after injection of WT fibrils and V40G multimers stained with GFAP (marker of astrogliosis). Scale bar, 50 μm. **b-e** Optical density of areas covered by GFAP immunoreactivity at 2, 4, and 10 weeks after injection in the striatum (at 2, 4, and 10 weeks, respectively: PBS, *n*=6, 6, 6; WT-syn, *n*=5, 6, 6; V40G, *n*=6, 7, 7) (**b**), the motor cortex (PBS, *n*=6, 6, 6; WT-syn, *n*=5, 7, 7; V40G, *n*=6, 7, 7) (**c**), the rhinal cortex (PBS, *n*=5, 6, 5; WT-syn, *n*=4, 7, 6; V40G, *n*=6, 7, 7) (**d**), and the amygdala (PBS, *n*=5, 6, 6; WT-syn, *n*=5, 7, 7; V40G, *n*=6, 7, 7) (**e**). Data are expressed as the mean ± SEM, one-way ANOVA with Tukey's post hoc test, two-sided, **P*<0.05, ***P*<0.01, ****P*<0.0001.

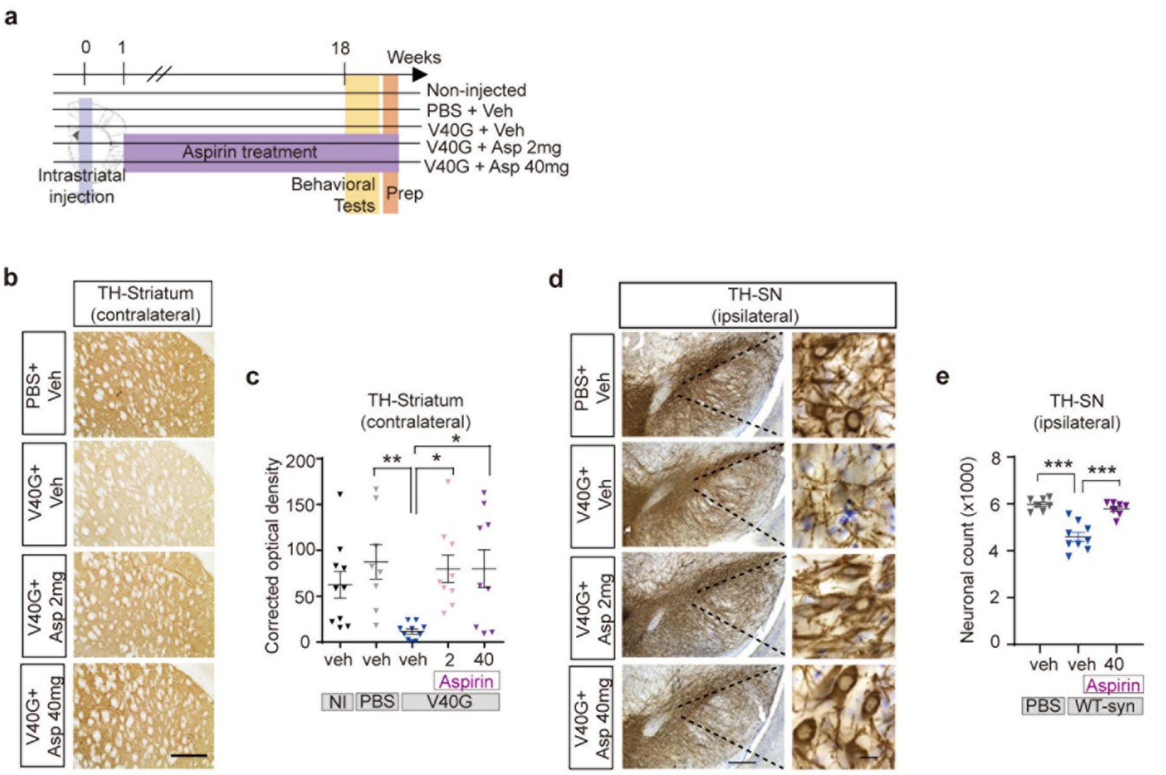


**Supplementary Fig. 7. Representative images of dopaminergic terminal and cell bodies by aspirin treatment. a** Oral administration of aspirin at approximately 2 mg/kg and 40 mg/kg via drinking water starting 1 week after intrastriatal injection in C57BL/6 mice. **b** Representative images of the striatal region stained with TH. Scale bar, 200 µm. **c** Optical density of the contralateral striatal region covered by TH immunoreactivity. (Noninjected, *n*=10; PBS+vehicle, *n*=8; V40G+vehicle, *n*=10; V40G+aspirin 2 mg, *n*=9; V40G+aspirin 40 mg, *n*=9). Data are expressed as the mean ± SEM, one-way ANOVA with Tukey's post hoc test, two-sided. **d** Representative images of the substantia nigra pars compacta stained with TH. Scale bar, low magnification, 200 µm; high magnification, 10 µm. **e** Stereological cell counts of TH-immunoreactive dopaminergic neurons in the substantia nigra pars compacta of mice that were injected with either PBS or WT-syn fibrils. Following these injections, animals were treated with vehicle or aspirin (PBS+vehicle, *n*=7; WT-syn+vehicle, *n*=9; WT-syn+aspirin 40 mg, *n*=7). Data are expressed as the mean ± SEM, one-way ANOVA with Tukey's post hoc test, two-sided, *$P<0.05$, **$P<0.01$, ***$P<0.0001$.

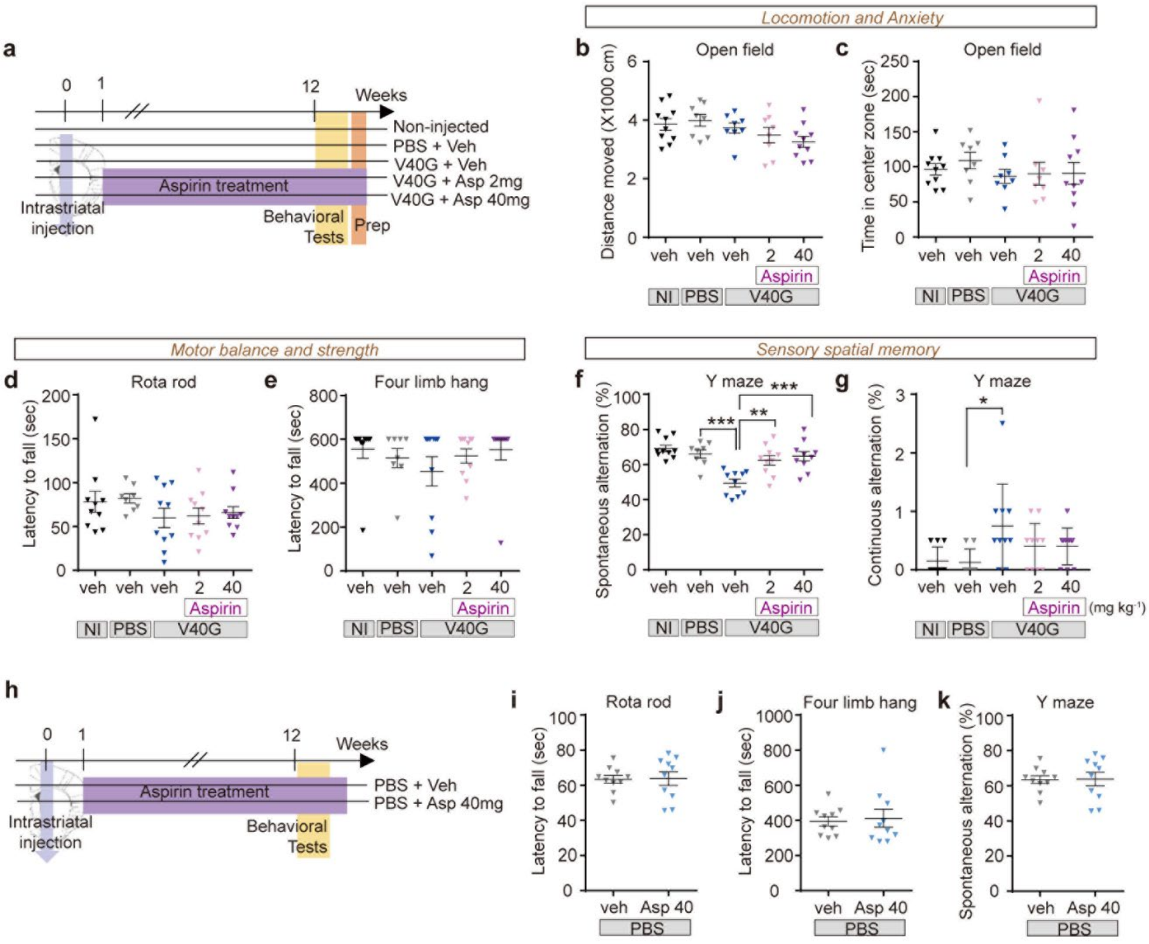


**Supplementary Fig. 8. Behavioral assessment of V40G-injected and PBS-injected mice after 12 weeks and 18 weeks of oral aspirin administration. a** Oral administration of aspirin at approximately 2 mg/kg and 40 mg/kg via drinking water starting 1 week after intrastriatal injection in C57BL/6 mice. **b-g** Twelve weeks after the intrastriatal injection, we performed open field, rotarod, four-limb hanging and Y maze behavioral tests. **b, c** Distance moved (cm) and time in center (s) for analysis of locomotion and anxiety (noninjected (NI), *n*=10; PBS+vehicle, *n*=8; V40G+vehicle, *n*=8; V40G+aspirin 2 mg, *n*=8; V40G+aspirin 40 mg, *n*=10). **d, e** Latency to fall (s) in rotarod and four-limb hanging tests for analysis of motor balance and strength (NI, *n*=10; PBS+vehicle, *n*=8; V40G+vehicle, *n*=10; V40G+aspirin 2 mg, *n*=10; V40G+aspirin 40 mg, *n*=10). **f, g** Spontaneous alteration (%) and continuous alteration (%) in the Y maze for analysis of sensory spatial memory (noninjected, *n*=10;

PBS+vehicle, *n*=8; V40G+vehicle, *n*=10; V40G+aspirin 2 mg, *n*=10; V40G+aspirin 40 mg, n=10). **h** Oral administration of aspirin at 40 mg/kg via the drinking water for 17 weeks, starting 1 week after injection of PBS in C57BL/6 mice. **i-k** Eighteen weeks after the initial intervention, we subjected the mice to the rotarod, four-limb hanging, and Y maze behavioral tests. (PBS+vehicle, *n*=10; PBS+aspirin 40 mg, *n*=10). Data are expressed as the mean ± SEM, one-way ANOVA with Tukey's post hoc test, two-sided, $*P<0.05$, $**P<0.01$, $***P<0.0001$.

| level | % area | |
|---|---|---|
| - | ~ 0.5 | No pathology |
| + | 0.5 ~ 1.5 | Mild pathology |
| ++ | 1.5 ~ 2.5 | Moderate pathology |
| +++ | 2.5 ~ 3.5 | Dense pathology |
| ++++ | 3.5 ~ | Severe pathology |

| | | 2 weeks | | | | | | 4 weeks | | | | | | 10 weeks | | | | | |
|---|---|---|---|---|---|---|---|---|---|---|---|---|---|---|---|---|---|---|---|
| Bregma | | PBS | | WT-syn | | V40G | | PBS | | WT-syn | | V40G | | PBS | | WT-syn | | V40G | |
| (mm) | | cont | ipsi | cont | ipsi | cont | ipsi | cont | ipsi | cont | ipsi | cont | ipsi | cont | ipsi | cont | ipsi | cont | ipsi |
| 0.86 | cingulate cortex | - | - | - | - | - | - | - | - | - | - | + | + | - | - | - | + | ++++ | ++++ |
| | motor cortex - | - | - | - | - | - | - | - | - | - | - | - | + | - | - | - | + | ++ | ++++ |
| | somatosensory cortex | - | - | - | - | - | + | - | - | - | + | - | + | - | - | - | + | + | ++ |
| | insular cortex - | - | - | - | - | - | - | - | - | - | + | - | + | - | - | - | + | + | ++ |
| | piriform cortex | - | - | - | - | - | - | - | - | - | - | - | + | - | - | - | - | - | + |
| | claustrum - | - | - | - | - | - | - | - | - | - | - | - | + | - | - | - | - | + | ++ |
| | striatum - | - | - | - | - | - | + | - | - | - | + | - | ++++ | - | - | - | + | + | ++++ |
| | nucleus accumbens | - | - | - | - | - | - | - | - | - | - | + | - | - | - | - | - | - | - |
| -1.82 | retrosplenial cortex | - | - | - | - | + | + | + | - | + | + | + | ++ | - | - | + | + | ++++ | ++++ |
| | parietal cortex | - | - | - | - | + | + | - | - | + | + | + | + | - | - | + | + | + | ++++ |
| | somatosensory cortex | - | - | - | - | - | + | - | - | - | + | + | + | - | - | - | + | + | ++ |
| | auditory cortex | - | - | - | - | - | - | - | - | - | - | - | - | - | - | - | - | + | ++ |
| | rhinal cortex - | - | - | - | - | - | - | - | - | - | - | - | - | - | - | - | + | ++++ | ++++ |
| | piriform cortex | - | - | - | - | - | - | - | - | - | - | - | - | - | - | - | + | - | + |
| | endopiriform nucleus | - | - | - | - | - | - | - | - | - | - | - | - | - | - | - | - | - | - |
| | hippocampus - | - | - | - | - | - | - | - | - | - | - | - | - | - | - | - | - | - | - |
| | habenular nucleus | - | - | - | - | - | + | - | - | - | + | - | + | - | - | - | + | + | + |
| | hypothalamus | - | - | - | - | - | - | - | - | - | - | - | - | - | - | - | - | - | - |
| | basolateral amygdala | - | - | - | - | - | - | - | - | - | - | + | ++++ | - | - | - | ++ | ++++ | ++++ |
| | central amygdala | - | - | - | - | - | - | - | - | - | - | - | - | - | - | - | - | - | ++++ |
| -2.8 | retrosplenial cortex | - | - | - | - | - | - | - | - | - | - | - | - | - | - | - | + | + | + |
| | visual cortex - | - | - | - | - | - | - | - | - | - | - | - | - | - | - | + | + | + | + |
| | auditory cortex | - | - | - | - | - | - | - | - | - | - | - | - | - | - | - | - | + | + |
| | rhinal cortex - | - | - | - | - | - | - | - | - | - | - | - | + | - | - | - | + | ++ | ++++ |
| | piriform cortex | - | - | - | - | - | - | - | - | - | - | - | - | - | - | - | + | + | + |
| | hippocampus - | - | - | - | - | - | - | - | - | - | - | - | - | - | - | - | - | - | - |
| | antpretectal nucleus | - | - | - | - | - | - | - | - | - | - | - | + | - | - | - | + | + | + |
| | substantia nigra | - | - | - | - | - | + | - | - | - | + | + | ++ | - | - | + | ++ | + | ++ |
| | retomammillary nucleus | - | - | - | - | - | - | - | - | - | - | - | - | - | - | - | - | - | - |

**Supplementary Table 1. Representative profiles of α-synuclein pathology in mouse brain regions.** Semiquantitative grading of α-synuclein pathology in mice injected with WT-syn fibrils and V40G multimers. Phospho-α-synuclein (pS129) expression levels in each region were evaluated in brain sections from six animals on a scale of 1 to 5, where 1 is the lowest in the area with less than 0.5% and 5 is the highest in the area with at least 3.5%, and the average values for each region were rounded up or down.

**Supplementary Table 2. The list of differentially expressed genes (DEGs) in the mice treated with WT-syn and V40G-syn relative to those of PBS-treated mice.**

log2 [fold change] ≥ 0.5, $p < 0.05$.

**RNAsequencing data for WT-syn vs PBS-enriched genes**

| Chip ID | Entrez ID | Gene syml | baseMean | log2FoldCl | lfcSE | stat | Pvalue |
|---|---|---|---|---|---|---|---|
| 3795 | . | Gm1564 | 3.995067 | 5.888904 | 1.385167 | 4.251404 | 1.06E-05 |
| 16161 | 214292 | Syna | 3.580625 | 4.802226 | 1.355549 | 3.542643 | 0.000198 |
| 8729 | 14960 | H2-Aa | 37.23049 | 1.496736 | 0.440028 | 3.401456 | 0.000335 |
| 2163 | 71086 | 4933412E1 | 30.76756 | -1.05505 | 0.321216 | -3.28455 | 0.000511 |
| 22885 | 668415 | Gm9159 | 20.44109 | 1.25998 | 0.387835 | 3.248756 | 0.00058 |
| 3063 | 70419 | 2810408A | 3.238297 | -4.53651 | 1.432397 | -3.16708 | 0.00077 |
| 4052 | 12416 | Cbx2 | 13.73004 | 1.705278 | 0.538657 | 3.165798 | 0.000773 |
| 4008 | 1E+08 | Gm11744 | 25.81312 | -1.28646 | 0.40663 | -3.1637 | 0.000779 |
| 944 | 226541 | Klhl20 | 118.8819 | 0.591248 | 0.18931 | 3.123173 | 0.000895 |
| 22172 | 20264 | Scn10a | 6.147105 | -3.24807 | 1.048554 | -3.09766 | 0.000975 |
| 13000 | 404710 | Iqgap3 | 6.637513 | -2.91253 | 0.947341 | -3.07443 | 0.001055 |
| 9339 | 1E+08 | Gm20939 | 11.33557 | -1.83791 | 0.598873 | -3.06894 | 0.001074 |
| 2506 | 72735 | Eldr | 3.016847 | -4.77108 | 1.579655 | -3.02033 | 0.001262 |
| 7303 | 105833 | Ccdc65 | 64.27417 | 0.69901 | 0.2341 | 2.985946 | 0.001414 |
| 6058 | 12159 | Bmp4 | 65.94479 | 0.952254 | 0.324271 | 2.936603 | 0.001659 |
| 8284 | 21646 | Tcte2 | 26.93481 | -1.15264 | 0.397283 | -2.90132 | 0.001858 |
| 12065 | 12075 | Bfsp1 | 13.88427 | 2.306848 | 0.798024 | 2.890701 | 0.001922 |
| 17356 | 232345 | A2m | 2.826634 | 4.404468 | 1.523794 | 2.890463 | 0.001923 |
| 17761 | 545902 | Ptprh | 12.23173 | -2.15786 | 0.765981 | -2.81712 | 0.002423 |
| 1664 | 67307 | Pbld2 | 6.393714 | 2.458607 | 0.87407 | 2.812827 | 0.002455 |
| 20267 | 110877 | Slc18a1 | 2.392728 | -4.77994 | 1.700856 | -2.81032 | 0.002475 |
| 15825 | 18587 | Pde6b | 2.10568 | 5.009355 | 1.79948 | 2.783779 | 0.002686 |
| 5449 | 71226 | 4933433G | 15.96835 | 1.515587 | 0.551337 | 2.748932 | 0.002989 |
| 6192 | 16475 | Ajuba | 16.96666 | -1.31034 | 0.477529 | -2.74399 | 0.003035 |
| 9099 | 104349 | Zfp119a | 10.52443 | -1.67079 | 0.61395 | -2.72138 | 0.003251 |
| 20212 | 15446 | Hpgd | 16.55331 | 1.492977 | 0.550705 | 2.711029 | 0.003354 |
| 3359 | 1E+08 | AI662270 | 4.470851 | -3.78592 | 1.398579 | -2.70698 | 0.003395 |
| 3488 | 278507 | Wfikkn2 | 13.39449 | 1.65331 | 0.612201 | 2.700599 | 0.003461 |
| 9383 | 338363 | Tmem241 | 23.77401 | 1.121665 | 0.416222 | 2.694868 | 0.003521 |
| 5544 | 75507 | Pou5f2 | 28.03906 | 1.034547 | 0.384061 | 2.693707 | 0.003533 |
| 8728 | 14961 | H2-Ab1 | 21.45639 | 1.469209 | 0.546033 | 2.690696 | 0.003565 |
| 15674 | 20311 | Cxcl5 | 2.310948 | 4.039698 | 1.514861 | 2.666711 | 0.00383 |
| 4732 | 1E+08 | Scarna13 | 5.096171 | 3.431561 | 1.295371 | 2.649095 | 0.004035 |
| 6892 | 68311 | Lypd2 | 5.042407 | 2.840885 | 1.073336 | 2.646781 | 0.004063 |
| 1377 | 237313 | Il20ra | 3.27531 | 4.043138 | 1.528283 | 2.645542 | 0.004078 |
| 13535 | 12368 | Casp6 | 23.80567 | 1.15277 | 0.436504 | 2.640917 | 0.004134 |
| 12994 | 18008 | Nes | 52.56635 | 0.744422 | 0.282226 | 2.637681 | 0.004174 |
| 16923 | 68169 | Ndnf | 62.74927 | 0.706638 | 0.268148 | 2.635254 | 0.004204 |
| 4078 | 238021 | Fscn2 | 2.049871 | 4.540918 | 1.727209 | 2.629049 | 0.004281 |
| 20202 | 11593 | Aga | 62.10019 | 0.665611 | 0.253534 | 2.625328 | 0.004328 |
| 7011 | 72709 | C1qtnf6 | 7.528454 | 2.029511 | 0.774596 | 2.62009 | 0.004395 |
| 21530 | . | AI118078 | 9.960622 | 1.649029 | 0.629709 | 2.618718 | 0.004413 |
| 15347 | 22612 | Yes1 | 33.72024 | 0.861024 | 0.329609 | 2.612261 | 0.004497 |
| 7404 | 16678 | Krt1 | 29.11408 | 0.928538 | 0.355822 | 2.609561 | 0.004533 |
| 14413 | 1.01E+08 | AV051173 | 5.98931 | 2.496718 | 0.957885 | 2.606491 | 0.004574 |
| 2014 | 237436 | Gas2l3 | 36.28377 | 0.879387 | 0.337935 | 2.60224 | 0.004631 |
| 1332 | 382450 | Gm5177 | 5.728868 | 2.137629 | 0.827647 | 2.582778 | 0.0049 |
| 5066 | 15216 | Hfe | 11.9044 | 1.424126 | 0.553247 | 2.574122 | 0.005025 |
| 22555 | 1.03E+08 | Gm35612 | 32.6696 | 0.847417 | 0.329223 | 2.573995 | 0.005027 |
| 7663 | 68162 | A930003A | 2.228014 | 3.916489 | 1.52965 | 2.560383 | 0.005228 |
| 18390 | 71909 | Haus5 | 13.39649 | -1.33618 | 0.522795 | -2.55583 | 0.005297 |
| 2213 | 668272 | Gm9079 | 6.764211 | -2.40633 | 0.951153 | -2.52991 | 0.005705 |
| 4496 | 27260 | Plek2 | 2.076614 | 4.110334 | 1.62561 | 2.528487 | 0.005728 |
| 340 | 12964 | Cryga | 11.70465 | 2.336484 | 0.932328 | 2.506076 | 0.006104 |
| 20826 | 436062 | Fam92b | 3.807102 | -2.54948 | 1.020703 | -2.49777 | 0.006249 |
| 17744 | 77323 | 9430041J1 | 6.819381 | -1.88012 | 0.757635 | -2.48156 | 0.00654 |
| 8138 | 246133 | Kcne2 | 3.000986 | -4.16447 | 1.681568 | -2.47654 | 0.006633 |
| 7522 | 14811 | Grin2a | 44.37844 | 0.765081 | 0.308988 | 2.476087 | 0.006642 |
| 2386 | 67596 | Tespa1 | 2.684161 | 3.873082 | 1.5692 | 2.468189 | 0.00679 |
| 17982 | 26436 | Psg16 | 15.95703 | -1.18958 | 0.48246 | -2.46566 | 0.006838 |
| 20238 | 234311 | Ddx60 | 8.286028 | -1.63895 | 0.664897 | -2.46497 | 0.006851 |
| 15507 | 21897 | Tlr1 | 1.585038 | 4.552814 | 1.849032 | 2.462268 | 0.006903 |
| 1524 | 75973 | Ccdc162 | 4.103663 | 3.293575 | 1.338045 | 2.461482 | 0.006918 |
| 6410 | 1.01E+08 | Gm16677 | 13.17898 | -1.41565 | 0.57644 | -2.45584 | 0.007028 |
| 9110 | 27221 | Chaf1a | 25.37082 | -1.02121 | 0.421365 | -2.42359 | 0.007684 |
| 5582 | 218441 | Zfyve16 | 43.94218 | 0.763192 | 0.315406 | 2.419712 | 0.007766 |
| 13437 | 229731 | Slc25a24 | 38.47035 | 0.701685 | 0.290691 | 2.413854 | 0.007892 |
| 710 | 22789 | Zp3r | 2.835733 | -3.58503 | 1.490712 | -2.40491 | 0.008088 |
| 6335 | 71000 | 4931440J1 | 2.202776 | -4.11366 | 1.719235 | -2.39272 | 0.008362 |
| 14474 | 100470 | Lao1 | 1.593678 | 4.263472 | 1.783172 | 2.390948 | 0.008402 |
| 14553 | 192199 | Rspo1 | 20.38475 | -1.27428 | 0.533602 | -2.38807 | 0.008468 |
| 6373 | 71988 | Esco2 | 1.536082 | 4.582115 | 1.929828 | 2.374364 | 0.00879 |
| 19093 | 259097 | Olfr558 | 2.306247 | -3.99894 | 1.686055 | -2.37177 | 0.008851 |
| 10397 | 667373 | Ifit1bl1 | 33.7076 | 0.800465 | 0.337699 | 2.370355 | 0.008886 |
| 4899 | 320581 | Idi2 | 2.11088 | 3.627141 | 1.531439 | 2.368453 | 0.008931 |
| 6139 | 19752 | Rnase1 | 5.649247 | -2.35776 | 0.998872 | -2.36042 | 0.009127 |
| 5903 | 71106 | 4933413J0 | 1.995445 | 4.305057 | 1.823976 | 2.360259 | 0.009131 |
| 7226 | 54526 | Syt10 | 31.31858 | 0.910368 | 0.387022 | 2.352238 | 0.00933 |
| 23176 | 237178 | Ppef1 | 8.196345 | 1.611522 | 0.686096 | 2.348829 | 0.009416 |
| 7906 | 208154 | Btla | 7.047644 | 2.007875 | 0.85513 | 2.348036 | 0.009436 |
| 6072 | 16854 | Lgals3 | 7.880105 | 1.79542 | 0.768204 | 2.337167 | 0.009715 |
| 6210 | 17888 | Myh6 | 20.92557 | 1.019344 | 0.43675 | 2.333931 | 0.0098 |
| 18962 | 70054 | Ccdc89 | 1.999543 | 4.312229 | 1.848002 | 2.333454 | 0.009812 |
| 1898 | 432478 | Tmprss9 | 5.218968 | 2.046636 | 0.878224 | 2.330426 | 0.009892 |
| 8146 | 209195 | Clic6 | 4.783747 | 2.371193 | 1.020296 | 2.324026 | 0.010062 |
| 17837 | 76373 | Zfp773 | 11.81816 | 1.519039 | 0.656081 | 2.315323 | 0.010298 |

**RNAsequencing data for V40G vs PBS-enriched genes**

| Chip ID | Entrez ID | Gene syml | baseMean | log2FoldCl | lfcSE | stat | Pvalue |
|---|---|---|---|---|---|---|---|
| 17611 | 654818 | Smco3 | 26.9654 | -2.17294 | 0.496658 | -4.37514 | 6.07E-06 |
| 18965 | 68472 | Tmem126l | 28.68688 | -1.29674 | 0.332942 | -3.8948 | 4.91E-05 |
| 14070 | 17841 | Mup2 | 8.63462 | -3.63738 | 0.942405 | -3.85968 | 5.68E-05 |
| 12994 | 18008 | Nes | 52.56635 | 1.045817 | 0.278487 | 3.755348 | 8.66E-05 |
| 16161 | 214292 | Syna | 3.580625 | 4.964528 | 1.350056 | 3.677276 | 0.000118 |
| 16982 | 21415 | Tcf7l1 | 87.0756 | 0.726914 | 0.19771 | 3.67667 | 0.000118 |
| 18174 | 16534 | Kcnn4 | 4.094941 | -5.63404 | 1.539625 | -3.65936 | 0.000126 |
| 4215 | 69513 | 1700030C | 26.21988 | 1.364947 | 0.380938 | 3.583119 | 0.00017 |
| 1679 | 13654 | Egr2 | 50.07564 | -1.06518 | 0.300343 | -3.54653 | 0.000195 |
| 174 | 1E+08 | Snord89 | 189.9123 | 1.33717 | 0.386977 | 3.455428 | 0.000275 |
| 23067 | 66240 | Kcne1l | 29.21725 | -1.28651 | 0.376539 | -3.41666 | 0.000317 |
| 9516 | 1E+08 | Nrg2 | 57.74203 | -1.03989 | 0.305999 | -3.39834 | 0.000339 |
| 5170 | 69666 | Psmg4 | 121.4291 | 0.63987 | 0.188898 | 3.387387 | 0.000353 |
| 4732 | 1E+08 | Scarna13 | 5.096171 | 4.264089 | 1.277547 | 3.337717 | 0.000422 |
| 4724 | 20714 | Serpina3k | 13.18313 | -2.42479 | 0.734291 | -3.30222 | 0.00048 |
| 23131 | 245670 | Rragb | 56.39324 | 0.860325 | 0.2654 | 3.241619 | 0.000594 |
| 2163 | 71086 | 4933412E1 | 30.76756 | -1.02466 | 0.318989 | -3.21222 | 0.000659 |
| 9 | 18387 | Oprk1 | 95.99017 | 0.751557 | 0.234516 | 3.204718 | 0.000676 |
| 2034 | 71761 | Amdhd1 | 5.836456 | -3.00643 | 0.938753 | -3.20258 | 0.000681 |
| 2386 | 67596 | Tespa1 | 2.684161 | 4.890528 | 1.535524 | 3.184925 | 0.000724 |
| 19740 | 54123 | Irf7 | 5.827411 | -2.90191 | 0.922769 | -3.14478 | 0.000831 |
| 12447 | 241794 | Kcng1 | 14.08549 | -1.73372 | 0.551418 | -3.14412 | 0.000833 |
| 6928 | 105732 | Fam83h | 11.6727 | 1.800143 | 0.574708 | 3.132274 | 0.000867 |
| 3253 | 67477 | Abhd15 | 8.892718 | 2.176758 | 0.695039 | 3.131852 | 0.000869 |
| 2938 | 11541 | Adora2b | 20.13623 | -1.56503 | 0.506612 | -3.08921 | 0.001003 |
| 16762 | 232785 | Zfp783 | 34.93732 | 1.174619 | 0.386533 | 3.038863 | 0.001187 |
| 17457 | 19362 | Rad51ap1 | 14.67149 | 1.53283 | 0.506278 | 3.027643 | 0.001232 |
| 4899 | 320581 | Idi2 | 2.11088 | 4.502545 | 1.49429 | 3.013166 | 0.001293 |
| 21446 | 434402 | Gm5617 | 37.11712 | 0.908107 | 0.302314 | 3.003852 | 0.001333 |
| 14450 | 230676 | Szt2 | 77.61744 | 0.660862 | 0.220396 | 2.998514 | 0.001356 |
| 16936 | 13197 | Gadd45a | 39.52497 | -1.06706 | 0.356522 | -2.99298 | 0.001381 |
| 21687 | 76459 | Car12 | 86.43106 | 0.631501 | 0.211289 | 2.98881 | 0.0014 |
| 15674 | 20311 | Cxcl5 | 2.310948 | 4.462393 | 1.498168 | 2.978566 | 0.001448 |
| 20366 | 72297 | B3gnt3 | 3.16922 | 4.629564 | 1.565042 | 2.958109 | 0.001548 |
| 8043 | 1.01E+08 | E330011O | 5.171381 | 3.298685 | 1.122078 | 2.9398 | 0.001642 |
| 7716 | 1E+08 | Snora81 | 101.8269 | 0.688289 | 0.236486 | 2.910484 | 0.001804 |
| 8729 | 14960 | H2-Aa | 37.23049 | 1.280957 | 0.441395 | 2.902068 | 0.001854 |
| 10123 | 67531 | 5730408K( | 75.14332 | 0.632971 | 0.220074 | 2.876174 | 0.002013 |
| 18717 | 233246 | Ano5 | 4.693977 | -2.95903 | 1.034482 | -2.86039 | 0.002116 |
| 21435 | 215051 | Bud13 | 78.78933 | 0.589251 | 0.206152 | 2.858333 | 0.002129 |
| 7319 | 78733 | Troap | 2.061767 | -4.25372 | 1.488572 | -2.85759 | 0.002134 |
| 18308 | 50760 | Fbxo17 | 9.362556 | 1.989724 | 0.69896 | 2.846693 | 0.002209 |
| 9408 | 13507 | Dsc3 | 2.931014 | -4.51009 | 1.588741 | -2.83878 | 0.002264 |
| 11932 | 11552 | Adra2b | 2.071938 | 5.041317 | 1.77725 | 2.836583 | 0.00228 |
| 20651 | 234684 | Lrrc29 | 6.008315 | 2.484771 | 0.876604 | 2.834543 | 0.002295 |
| 20293 | . | 2310045N | 26.47773 | -1.11136 | 0.394889 | -2.81437 | 0.002444 |
| 6628 | 18414 | Osmr | 24.513 | -1.09055 | 0.387637 | -2.81333 | 0.002452 |
| 2505 | 13649 | Egfr | 30.11922 | -1.1966 | 0.426263 | -2.80719 | 0.002499 |
| 20708 | 97484 | Cog8 | 58.75089 | 0.81453 | 0.290644 | 2.802501 | 0.002535 |
| 4379 | 66603 | Gemin2 | 20.7391 | 1.249322 | 0.447886 | 2.789376 | 0.00264 |
| 14078 | 1E+08 | Mup13 | 3.024478 | -3.74743 | 1.345357 | -2.78545 | 0.002673 |
| 16749 | 54324 | Arhgef5 | 22.63825 | 1.242625 | 0.447015 | 2.779828 | 0.002719 |
| 4226 | 194655 | Klf11 | 21.82346 | -1.48107 | 0.535674 | -2.76488 | 0.002847 |
| 8688 | 378460 | Pram1 | 1.942607 | 4.805 | 1.73836 | 2.764099 | 0.002854 |
| 4748 | 78801 | Ak7 | 5.271884 | -2.50665 | 0.90871 | -2.75847 | 0.002904 |
| 6653 | 1E+08 | Gm10389 | 13.63372 | 1.479871 | 0.537048 | 2.755566 | 0.00293 |
| 7522 | 14811 | Grin2a | 44.37844 | 0.845463 | 0.307418 | 2.750204 | 0.002978 |
| 21881 | 319859 | E030011O | 23.09399 | 1.233063 | 0.448428 | 2.749748 | 0.002982 |
| 2120 | 368203 | Gm5136 | 11.54503 | 1.803782 | 0.661187 | 2.728097 | 0.003185 |
| 1 | 497097 | Xkr4 | 88.37402 | 0.599521 | 0.220541 | 2.718415 | 0.00328 |
| 16010 | 114643 | Oas1c | 5.221318 | 2.368023 | 0.874901 | 2.706618 | 0.003399 |
| 2482 | 16006 | Igfbp1 | 2.886717 | -3.82189 | 1.414881 | -2.70121 | 0.003454 |
| 6503 | 16840 | Lect1 | 3.224291 | -3.69426 | 1.369851 | -2.69683 | 0.0035 |
| 6023 | 11720 | Mat1a | 5.301468 | -2.61674 | 0.971417 | -2.69374 | 0.003533 |
| 18315 | 320435 | Rinl | 16.92895 | -1.38367 | 0.51472 | -2.68819 | 0.003592 |
| 674 | 319229 | Sctr | 2.827779 | -3.87598 | 1.44713 | -2.67839 | 0.003699 |
| 4191 | 627110 | Tubb2a-ps | 1.688477 | 4.475286 | 1.681321 | 2.661768 | 0.003887 |
| 706 | 12767 | Cxcr4 | 17.56502 | -1.41379 | 0.531387 | -2.66057 | 0.0039 |
| 13997 | 209186 | Acnat2 | 11.06497 | -1.44423 | 0.543158 | -2.65895 | 0.003919 |
| 20212 | 15446 | Hpgd | 16.55331 | 1.457657 | 0.550336 | 2.648665 | 0.004041 |
| 9592 | 93716 | Pcdhga8 | 1.816605 | -4.44249 | 1.680927 | -2.64288 | 0.00411 |
| 15752 | 69571 | 2310034O | 3.735203 | 3.705055 | 1.402117 | 2.642472 | 0.004115 |
| 1332 | 382450 | Gm5177 | 5.728868 | 2.178536 | 0.824816 | 2.641238 | 0.00413 |
| 1688 | 12534 | Cdk1 | 5.408716 | -2.83578 | 1.076251 | -2.63487 | 0.004208 |
| 14117 | 21923 | Tnc | 29.58405 | -1.03838 | 0.394229 | -2.63395 | 0.00422 |
| 245 | 78016 | Ccdc150 | 2.736348 | 4.20771 | 1.598906 | 2.631618 | 0.004249 |
| 20174 | 53318 | Pdlim3 | 7.673352 | 1.808544 | 0.688083 | 2.628382 | 0.00429 |
| 9383 | 338363 | Tmem241 | 23.77401 | 1.090735 | 0.415798 | 2.623232 | 0.004355 |
| 672 | 66343 | Tmem177 | 77.06508 | 0.624394 | 0.238141 | 2.621945 | 0.004371 |
| 20722 | 56523 | Pmfbp1 | 2.317174 | 3.980965 | 1.519529 | 2.619867 | 0.004398 |
| 1611 | 56709 | Dnajb12 | 62.01035 | 0.66124 | 0.254874 | 2.594377 | 0.004738 |
| 21415 | 16154 | Il10ra | 17.01454 | -1.2618 | 0.487616 | -2.58768 | 0.004831 |
| 14079 | 1E+08 | Mup14 | 2.471531 | -3.62749 | 1.409299 | -2.57397 | 0.005027 |
| 14596 | 74989 | Csmd2os | 28.57466 | 0.894701 | 0.348162 | 2.569781 | 0.005088 |
| 9910 | 17771 | Mtl5 | 2.005541 | -4.85277 | 1.892156 | -2.56468 | 0.005164 |
| 8706 | 54218 | B3galt4 | 10.92411 | 1.532076 | 0.597618 | 2.563639 | 0.005179 |

| | | | | | | | |
|---|---|---|---|---|---|---|---|
| 16864 | 1E+08 | Gm3279 | 6.141671 | 2.246983 | 0.972611 | 2.310258 | 0.010437 |
| 17352 | 24110 | Usp18 | 6.5099 | -1.98391 | 0.859031 | -2.30948 | 0.010459 |
| 6610 | 328489 | A630020A | 3.526066 | -3.23284 | 1.400785 | -2.30788 | 0.010503 |
| 13004 | 76927 | Tsacc | 12.23489 | -1.45685 | 0.631576 | -2.30668 | 0.010536 |
| 2075 | 14426 | Galnt4 | 24.31347 | 0.927721 | 0.40262 | 2.304212 | 0.010605 |
| 9235 | 268973 | Nlrc4 | 4.284664 | 2.550265 | 1.109153 | 2.29929 | 0.010744 |
| 6645 | 320277 | Spef2 | 13.19967 | -1.15053 | 0.502335 | -2.29036 | 0.011 |
| 21461 | 69631 | Plet1os | 1.901008 | 3.967971 | 1.734908 | 2.287137 | 0.011094 |
| 12047 | 74243 | Slx4ip | 14.42255 | 1.391521 | 0.610625 | 2.278848 | 0.011338 |
| 18529 | 23993 | Klk7 | 1.541543 | 4.528528 | 1.987505 | 2.278498 | 0.011348 |
| 21672 | 208084 | Pif1 | 5.657664 | 2.004341 | 0.880254 | 2.277004 | 0.011393 |
| 3332 | 71388 | 5530401A | 19.68521 | -1.08138 | 0.475857 | -2.27249 | 0.011528 |
| 5998 | 105450 | Mmrn2 | 34.44801 | 0.819905 | 0.362064 | 2.26453 | 0.011771 |
| 854 | 18783 | Pla2g4a | 6.392218 | -1.85885 | 0.823821 | -2.25638 | 0.012023 |
| 20708 | 97484 | Cog8 | 58.75089 | 0.659218 | 0.292593 | 2.253024 | 0.012129 |
| 20651 | 234684 | Lrrc29 | 6.008315 | 2.000168 | 0.890354 | 2.246486 | 0.012336 |
| 12766 | 1E+08 | Gm2011 | 7.96855 | 1.741203 | 0.777288 | 2.240101 | 0.012542 |
| 5596 | 105364 | AW495222 | 34.04295 | 0.800468 | 0.358133 | 2.235113 | 0.012705 |
| 16480 | 269823 | Pon3 | 8.263027 | -1.62436 | 0.728118 | -2.2309 | 0.012844 |
| 16491 | 13395 | Dlx5 | 17.53122 | 1.341077 | 0.601235 | 2.230537 | 0.012856 |
| 16749 | 54324 | Arhgef5 | 22.63825 | 1.004842 | 0.451337 | 2.226366 | 0.012995 |
| 12134 | 114889 | Vsx1 | 2.171182 | 3.512558 | 1.582781 | 2.219232 | 0.013235 |
| 8070 | 224419 | Map3k7cl | 1.759445 | 3.590031 | 1.62285 | 2.212177 | 0.013477 |
| 14955 | 11610 | Agtrap | 52.39122 | 0.675165 | 0.30643 | 2.203324 | 0.013786 |
| 964 | 55990 | Fmo2 | 3.660695 | -3.52758 | 1.605756 | -2.19683 | 0.014016 |
| 9168 | 69239 | Pdzph1 | 15.11718 | -1.22797 | 0.559848 | -2.19341 | 0.014139 |
| 15752 | 69571 | 2310034O | 3.735203 | 3.102397 | 1.41831 | 2.18739 | 0.014357 |
| 4298 | 71156 | Lrrc72 | 1.982421 | -4.50523 | 2.060898 | -2.18605 | 0.014406 |
| 19740 | 54123 | Irf7 | 5.827411 | -1.82522 | 0.835039 | -2.1858 | 0.014415 |
| 18276 | 545938 | Zfp607 | 3.81396 | 2.42814 | 1.111608 | 2.184348 | 0.014468 |
| 16521 | 21753 | Tes | 30.58809 | 0.942649 | 0.432255 | 2.180773 | 0.0146 |
| 4648 | 14744 | Gpr65 | 2.104784 | 3.816828 | 1.761499 | 2.166806 | 0.015125 |
| 674 | 319229 | Sctr | 2.827779 | -2.72339 | 1.258156 | -2.16459 | 0.01521 |
| 14941 | 112422 | 2610305D | 4.336371 | 1.995721 | 0.922707 | 2.162897 | 0.015275 |
| 10420 | 15242 | Hhex | 11.80253 | 1.329181 | 0.614735 | 2.162201 | 0.015301 |
| 5497 | 16371 | Irx1 | 7.938644 | 1.634804 | 0.758358 | 2.155715 | 0.015553 |
| 15215 | 320719 | 6030443J0 | 3.170345 | 2.43571 | 1.133139 | 2.149525 | 0.015796 |
| 6416 | 21933 | Tnfrsf10b | 10.95229 | -1.3966 | 0.652374 | -2.14079 | 0.016145 |
| 11844 | 1.01E+08 | AV039307 | 7.609182 | -1.63258 | 0.762997 | -2.1397 | 0.01619 |
| 619 | 227394 | Slco4c1 | 4.881842 | 2.322446 | 1.085545 | 2.139428 | 0.016201 |
| 20543 | 17390 | Mmp2 | 10.88548 | -1.21733 | 0.570311 | -2.1345 | 0.016401 |
| 9790 | 17202 | Mc4r | 40.67727 | 0.68116 | 0.319136 | 2.13439 | 0.016405 |
| 9297 | 20532 | Slc3a1 | 12.85443 | 1.102606 | 0.516753 | 2.133719 | 0.016433 |
| 16780 | 107526 | Gimap4 | 8.431274 | 2.087413 | 0.978467 | 2.13335 | 0.016448 |
| 23061 | 94216 | Col4a6 | 6.100952 | -1.79575 | 0.842192 | -2.13223 | 0.016494 |
| 2994 | 71860 | Cfap52 | 2.700742 | 2.765509 | 1.298177 | 2.130302 | 0.016573 |
| 5335 | 58992 | F12 | 9.959301 | -1.54932 | 0.727922 | -2.12841 | 0.016652 |
| 17772 | 664968 | Tmem238 | 16.36 | 1.138179 | 0.53488 | 2.127913 | 0.016672 |
| 8063 | 23794 | Adamts5 | 19.80599 | 1.072966 | 0.50452 | 2.126706 | 0.016722 |
| 494 | 319336 | C130036L | 20.23846 | 0.958814 | 0.450917 | 2.126364 | 0.016736 |
| 11806 | 20732 | Spint1 | 7.763763 | 1.48828 | 0.700157 | 2.125637 | 0.016767 |
| 8607 | 74116 | Pi16 | 1.268599 | 4.186172 | 1.969625 | 2.125365 | 0.016778 |
| 4748 | 78801 | Ak7 | 5.271884 | -1.81847 | 0.855711 | -2.12509 | 0.016789 |
| 6454 | 214254 | Nudt15 | 2.044679 | -3.44005 | 1.619715 | -2.12386 | 0.016841 |
| 19397 | 233781 | Xylt1 | 13.03802 | 1.226314 | 0.578154 | 2.121087 | 0.016957 |
| 23190 | 14829 | Grpr | 10.19228 | -1.35575 | 0.640307 | -2.11734 | 0.017116 |
| 20648 | 277978 | Exoc3l | 6.225513 | -1.83017 | 0.865209 | -2.11529 | 0.017203 |
| 12767 | 1E+08 | Gm10731 | 3.852079 | 2.001982 | 0.946858 | 2.114344 | 0.017243 |
| 6933 | 271278 | BC024139 | 2.978296 | -2.65459 | 1.261454 | -2.10439 | 0.017672 |
| 21447 | 18113 | Nnmt | 9.335856 | 1.248607 | 0.593894 | 2.102406 | 0.017759 |
| 21282 | 67776 | Vwa5a | 37.0466 | 0.658398 | 0.313995 | 2.096845 | 0.018004 |
| 16386 | 69282 | 1700001J0 | 1.319632 | -3.7121 | 1.776839 | -2.08916 | 0.018347 |
| 15058 | 544678 | Cfap74 | 15.76393 | -1.17243 | 0.561648 | -2.08748 | 0.018422 |
| 12385 | 71856 | Wfdc3 | 8.516573 | -1.67445 | 0.802789 | -2.0858 | 0.018499 |
| 10333 | 226049 | Dmrt2 | 2.856923 | -2.56626 | 1.230877 | -2.0849 | 0.018539 |
| 8802 | 15006 | H2-Q1 | 4.994351 | 2.046222 | 0.981855 | 2.084036 | 0.018578 |
| 11759 | 11464 | Actc1 | 1.634794 | 4.466481 | 2.150102 | 2.077334 | 0.018885 |
| 10897 | 13166 | Dbh | 4.617592 | -2.03245 | 0.978881 | -2.0763 | 0.018933 |
| 8480 | 407831 | Tmem204 | 48.69681 | 0.668177 | 0.322075 | 2.074601 | 0.019012 |
| 20982 | 71544 | Arhgap42 | 35.2892 | 0.709097 | 0.34187 | 2.074173 | 0.019032 |
| 17457 | 19362 | Rad51ap1 | 14.67149 | 1.069016 | 0.516489 | 2.069775 | 0.019237 |
| 7733 | 12737 | Cldn1 | 16.16808 | 1.074886 | 0.520694 | 2.064333 | 0.019493 |
| 9163 | 106628 | Trip10 | 30.78196 | 0.851928 | 0.41275 | 2.064031 | 0.019507 |
| 3102 | 14723 | Gp1ba | 2.890175 | 2.645857 | 1.281926 | 2.06397 | 0.01951 |
| 9210 | 246707 | Emilin2 | 9.062786 | -1.66821 | 0.808462 | -2.06344 | 0.019536 |
| 11623 | 11695 | Alx4 | 8.079335 | 1.950933 | 0.946931 | 2.060268 | 0.019686 |
| 20174 | 53318 | Pdlim3 | 7.673352 | 1.440769 | 0.699333 | 2.060205 | 0.019689 |
| 21473 | 66952 | 2310030G | 16.39533 | 1.073127 | 0.521332 | 2.058433 | 0.019774 |
| 7633 | 66779 | 4933432I0 | 8.944244 | -1.37307 | 0.667646 | -2.05658 | 0.019863 |
| 14293 | . | BC055111 | 1.429991 | -4.36492 | 2.123532 | -2.0555 | 0.019915 |
| 5147 | 66222 | Serpinb1a | 30.30422 | 0.816338 | 0.397248 | 2.054985 | 0.01994 |
| 2482 | 16006 | Igfbp1 | 2.886717 | -2.4395 | 1.19115 | -2.04802 | 0.020279 |
| 3604 | 69338 | 1700003D | 2.278574 | 3.287057 | 1.60651 | 2.046085 | 0.020374 |
| 4335 | 76826 | Nubpl | 10.51577 | 1.201998 | 0.587633 | 2.04549 | 0.020403 |
| 1712 | 11540 | Adora2a | 14.93768 | -1.14962 | 0.562419 | -2.04407 | 0.020473 |
| 979 | 14067 | F5 | 10.65554 | 1.328677 | 0.65172 | 2.038723 | 0.020739 |
| 4251 | 328075 | C630031E | 22.75884 | 0.913248 | 0.448543 | 2.036029 | 0.020874 |
| 3507 | 12842 | Col1a1 | 102.1109 | 0.956141 | 0.469682 | 2.035719 | 0.020889 |
| 4406 | 18974 | Pole2 | 3.306902 | -3.00351 | 1.477169 | -2.03329 | 0.021012 |

| | | | | | | | |
|---|---|---|---|---|---|---|---|
| 4709 | 20702 | Serpina1c | 4.500622 | -3.08862 | 1.207532 | -2.5578 | 0.005267 |
| 18790 | 78444 | Pgpep1l | 1.762087 | -4.64477 | 1.816075 | -2.55759 | 0.00527 |
| 14413 | 1.01E+08 | AV051173 | 5.98931 | 2.447364 | 0.957661 | 2.555564 | 0.005301 |
| 6950 | 20289 | Scx | 21.39309 | 1.269095 | 0.497143 | 2.552775 | 0.005343 |
| 7226 | 54526 | Syt10 | 31.31858 | 0.982611 | 0.385387 | 2.549676 | 0.005391 |
| 4014 | 193217 | BC018473 | 2.24113 | 3.888747 | 1.527898 | 2.545162 | 0.005461 |
| 10992 | 16819 | Lcn2 | 5.830331 | -2.93922 | 1.156766 | -2.5409 | 0.005528 |
| 7906 | 208154 | Btla | 7.047644 | 2.159219 | 0.850433 | 2.538965 | 0.005559 |
| 14611 | 74648 | S100pbp | 5.82929 | -2.47486 | 0.977562 | -2.53167 | 0.005676 |
| 19529 | 101602 | AI467606 | 12.92021 | -1.43858 | 0.56927 | -2.52706 | 0.005751 |
| 9161 | 12266 | C3 | 5.815559 | -2.19925 | 0.872824 | -2.51969 | 0.005873 |
| 13098 | 20197 | S100a3 | 7.09965 | 1.754139 | 0.696206 | 2.519567 | 0.005875 |
| 7011 | 72709 | C1qtnf6 | 7.528454 | 1.95255 | 0.775004 | 2.519407 | 0.005878 |
| 15740 | 14176 | Fgf5 | 20.22114 | -1.14864 | 0.457679 | -2.50971 | 0.006042 |
| 10233 | 12803 | Cntf | 9.529616 | -1.63231 | 0.651287 | -2.50628 | 0.0061 |
| 17840 | 76229 | Vmn2r29 | 26.18539 | -1.07281 | 0.428081 | -2.50609 | 0.006104 |
| 19047 | 18442 | P2ry2 | 1.915059 | -4.57687 | 1.837106 | -2.49135 | 0.006363 |
| 2075 | 14426 | Galnt4 | 24.31347 | 0.997805 | 0.400632 | 2.490575 | 0.006377 |
| 6778 | 239410 | A930017M | 32.84402 | 0.994112 | 0.3999 | 2.485902 | 0.006461 |
| 999 | 12503 | Cd247 | 12.73132 | -1.24966 | 0.503634 | -2.48128 | 0.006545 |
| 5675 | 76624 | 1700099I0 | 26.83691 | 1.00006 | 0.403324 | 2.479545 | 0.006578 |
| 8728 | 14961 | H2-Ab1 | 21.45639 | 1.352051 | 0.54674 | 2.472932 | 0.0067 |
| 10717 | 16169 | Il15ra | 25.89139 | -1.10458 | 0.447189 | -2.47006 | 0.006755 |
| 9656 | 107003 | A330093E | 4.667334 | -2.69453 | 1.099403 | -2.4509 | 0.007125 |
| 7265 | 105827 | Amigo2 | 51.31716 | 0.707177 | 0.288941 | 2.447476 | 0.007193 |
| 9781 | 319888 | Oacyl | 16.19636 | 1.461957 | 0.598251 | 2.44372 | 0.007268 |
| 6691 | 110082 | Dnah5 | 47.33264 | 0.709792 | 0.290791 | 2.440902 | 0.007325 |
| 12203 | 13436 | Dnmt3b | 7.114226 | -1.74552 | 0.717416 | -2.43306 | 0.007486 |
| 12754 | 24063 | Spry1 | 22.82049 | -1.11448 | 0.458206 | -2.43228 | 0.007502 |
| 6310 | 50769 | Atp8a2 | 10.83284 | 1.358955 | 0.558884 | 2.431552 | 0.007517 |
| 4251 | 328075 | C630031E | 22.75884 | 1.07805 | 0.445237 | 2.421294 | 0.007733 |
| 15225 | 67194 | 2700038G | 24.77513 | -1.00177 | 0.413963 | -2.41994 | 0.007762 |
| 11958 | 329513 | A730036I1 | 4.245088 | -2.4156 | 0.998643 | -2.41888 | 0.007784 |
| 13109 | 16939 | Lor | 58.61433 | 0.604918 | 0.251012 | 2.409916 | 0.007978 |
| 15994 | 1.01E+08 | Gm10390 | 4.356349 | -2.43803 | 1.011707 | -2.40981 | 0.00798 |
| 9790 | 17202 | Mc4r | 40.67727 | 0.764182 | 0.317423 | 2.40746 | 0.008032 |
| 11147 | 767815 | BB557941 | 13.28982 | 1.412887 | 0.59018 | 2.393992 | 0.008333 |
| 12394 | 415115 | Neurl2 | 14.52431 | -1.43613 | 0.602338 | -2.38425 | 0.008557 |
| 5738 | 14313 | Fst | 32.84158 | -1.16089 | 0.48889 | -2.37455 | 0.008785 |
| 13424 | 1E+08 | Scarna2 | 3.222527 | 3.629296 | 1.53173 | 2.36941 | 0.008908 |
| 15745 | 319818 | A930011G | 7.932272 | -1.83288 | 0.775504 | -2.36347 | 0.009052 |
| 5497 | 16371 | Irx1 | 7.938644 | 1.779444 | 0.753672 | 2.361033 | 0.009112 |
| 5449 | 71226 | 4933433G | 15.96835 | 1.299303 | 0.553872 | 2.345854 | 0.009492 |
| 15147 | 70358 | Steap1 | 1.93852 | 4.795894 | 2.044433 | 2.345831 | 0.009492 |
| 11901 | 20495 | Slc12a1 | 2.21437 | 4.02319 | 1.724626 | 2.33279 | 0.00983 |
| 21840 | 12044 | Bcl2a1a | 3.805778 | 2.074507 | 0.890203 | 2.330374 | 0.009893 |
| 20725 | 15439 | Hp | 7.711333 | -1.83252 | 0.786402 | -2.33026 | 0.009896 |
| 15063 | 1.01E+08 | Gm16023 | 13.44743 | 1.200589 | 0.515884 | 2.327243 | 0.009976 |
| 9034 | 107477 | Guca1b | 6.907668 | -2.01642 | 0.866894 | -2.32603 | 0.010008 |
| 5742 | 109700 | Itga1 | 14.01651 | 1.369269 | 0.588675 | 2.326018 | 0.010009 |
| 1048 | 11807 | Apoa2 | 10.30471 | -1.77559 | 0.764181 | -2.32353 | 0.010075 |
| 22534 | 74131 | Sash3 | 6.861773 | -1.85897 | 0.800218 | -2.32308 | 0.010087 |
| 16424 | 1.01E+08 | Gm15408 | 7.192071 | 1.733615 | 0.751917 | 2.305595 | 0.010567 |
| 5269 | 407800 | Ecm2 | 23.62399 | 0.914408 | 0.396929 | 2.303706 | 0.01062 |
| 6319 | 24053 | Sgcg | 1.542181 | -4.23915 | 1.840287 | -2.30353 | 0.010625 |
| 17906 | 381845 | Rnf225 | 3.587885 | -3.26177 | 1.422679 | -2.29269 | 0.010933 |
| 21461 | 69631 | Plet1os | 1.901008 | 3.959997 | 1.733042 | 2.284998 | 0.011156 |
| 14076 | . | Gm2083 | 8.676614 | -1.87605 | 0.821793 | -2.28287 | 0.011219 |
| 17981 | 53417 | Hif3a | 22.97844 | 1.044507 | 0.457896 | 2.281104 | 0.011271 |
| 6794 | 18383 | Tnfrsf11b | 3.362544 | -2.53652 | 1.112417 | -2.28019 | 0.011298 |
| 2550 | 19696 | Rel | 7.616753 | -1.64451 | 0.723162 | -2.27405 | 0.011481 |
| 21627 | 319480 | Itga11 | 6.026263 | -2.02719 | 0.891581 | -2.2737 | 0.011492 |
| 9598 | 93723 | Pcdhga11 | 44.20363 | 0.630173 | 0.277264 | 2.272828 | 0.011518 |
| 551 | 12835 | Col6a3 | 17.55708 | -1.07896 | 0.474737 | -2.27276 | 0.01152 |
| 6921 | 105734 | Tigd5 | 51.01308 | 0.736701 | 0.324437 | 2.270704 | 0.011582 |
| 18875 | 18584 | Pde8a | 13.81818 | 1.184955 | 0.522491 | 2.267897 | 0.011668 |
| 14344 | 230603 | Ttc39a | 9.471953 | -1.60642 | 0.70877 | -2.26649 | 0.011711 |
| 11596 | 14061 | F2 | 1.708741 | -3.79275 | 1.676856 | -2.26182 | 0.011854 |
| 365 | 381269 | Mreg | 34.23596 | 0.719041 | 0.318305 | 2.258972 | 0.011943 |
| 11180 | 71586 | Ifih1 | 15.35423 | -1.02303 | 0.454537 | -2.25071 | 0.012202 |
| 4459 | 18755 | Prkch | 17.07053 | -1.16385 | 0.517889 | -2.2473 | 0.012311 |
| 19465 | 73951 | 4930413G | 9.472092 | -1.3366 | 0.594967 | -2.24651 | 0.012336 |
| 21137 | 235050 | Zfp810 | 49.59103 | 0.679147 | 0.302921 | 2.241993 | 0.012481 |
| 9271 | 791338 | Gm10190 | 12.37915 | -1.31377 | 0.587265 | -2.2371 | 0.01264 |
| 8010 | 13840 | Epha6 | 28.97219 | 0.823371 | 0.368184 | 2.236301 | 0.012666 |
| 1569 | 215031 | Vgll2 | 3.988447 | 3.130551 | 1.401967 | 2.232971 | 0.012775 |
| 2757 | 257871 | Olfr1372-p | 2.365283 | -3.93819 | 1.76888 | -2.22638 | 0.012995 |
| 12086 | 627302 | Gm14092 | 1.946345 | 3.844035 | 1.728867 | 2.223442 | 0.013093 |
| 9590 | 93715 | Pcdhga7 | 1.633797 | 4.342641 | 1.9532 | 2.223347 | 0.013096 |
| 16768 | 243369 | Sspo | 3.251225 | -3.74109 | 1.684356 | -2.22108 | 0.013173 |
| 7958 | 56277 | Tmem45a | 4.717899 | -2.40789 | 1.085084 | -2.21908 | 0.013241 |
| 12804 | 242022 | Frem2 | 13.58959 | -1.33247 | 0.601305 | -2.21596 | 0.013347 |
| 23129 | 319781 | 9530051G | 25.54278 | 1.001829 | 0.452187 | 2.215521 | 0.013362 |
| 4818 | 77675 | 5033406O | 1.370917 | 4.414542 | 1.99948 | 2.207845 | 0.013628 |
| 14590 | 14612 | Gja4 | 15.64165 | 1.16448 | 0.527849 | 2.206084 | 0.013689 |
| 8730 | 14969 | H2-Eb1 | 25.88848 | 0.953247 | 0.43225 | 2.205314 | 0.013716 |
| 15052 | 76866 | Morn1 | 20.18006 | 1.125606 | 0.513505 | 2.192008 | 0.014189 |
| 18368 | 23900 | Hcst | 6.222308 | -1.6558 | 0.757452 | -2.18601 | 0.014407 |
| 12944 | 99571 | Fgg | 9.751279 | -1.91082 | 0.874682 | -2.18459 | 0.014459 |

| | | | | | | | |
|---|---|---|---|---|---|---|---|
| 1876 | 67331 | Atp8b3 | 1.285193 | -4.19976 | 2.066769 | -2.03204 | 0.021075 |
| 1020 | 66977 | Nuf2 | 13.71472 | -1.22856 | 0.605013 | -2.03063 | 0.021146 |
| 9466 | 402767 | A830052D | 9.542333 | -1.22682 | 0.605419 | -2.0264 | 0.021362 |
| 8043 | 1.01E+08 | E330011O | 5.171381 | 2.318205 | 1.146603 | 2.021803 | 0.021598 |
| 11317 | 21788 | Tfpi | 27.28021 | 0.804723 | 0.398307 | 2.020358 | 0.021673 |
| 7954 | 224224 | Impg2 | 13.98493 | 1.525313 | 0.755101 | 2.020013 | 0.021691 |
| 2634 | 319767 | Atp10b | 4.189394 | 2.57814 | 1.278946 | 2.015832 | 0.021909 |
| 3472 | 18559 | Pctp | 7.216951 | -1.49051 | 0.739561 | -2.01539 | 0.021932 |
| 20936 | 212728 | Gm17296 | 5.530775 | 1.636332 | 0.812346 | 2.014329 | 0.021988 |
| 1051 | 240913 | Adamts4 | 9.322636 | 1.426717 | 0.70841 | 2.013972 | 0.022006 |
| 22612 | 245424 | Gpr101 | 19.02032 | 0.847672 | 0.421232 | 2.012366 | 0.022091 |
| 21165 | 75469 | Spata19 | 7.261916 | -1.80785 | 0.899161 | -2.01059 | 0.022184 |
| 15261 | 242894 | Actr3b | 34.22517 | 0.650679 | 0.324036 | 2.008046 | 0.022319 |
| 354 | 13869 | Erbb4 | 14.80435 | 0.944249 | 0.470633 | 2.006341 | 0.02241 |
| 13509 | 64580 | Ndst4 | 49.50086 | 0.645573 | 0.322192 | 2.003694 | 0.022551 |
| 20271 | 211135 | D130040H | 10.15281 | 1.350998 | 0.675162 | 2.000999 | 0.022696 |
| 15043 | 242800 | Ttc34 | 9.478165 | 1.334135 | 0.66679 | 2.000831 | 0.022705 |
| 19350 | 1E+08 | Snora23 | 1.724265 | 4.128608 | 2.063749 | 2.000538 | 0.022721 |
| 10005 | 75555 | 1700020D | 7.27933 | -1.92402 | 0.961932 | -2.00016 | 0.022742 |
| 21677 | 68026 | 2810417H | 34.81165 | 0.790273 | 0.395818 | 1.996559 | 0.022937 |
| 19477 | 77035 | Kdm8 | 17.60973 | 1.010294 | 0.507203 | 1.991892 | 0.023191 |
| 17006 | 21336 | Tacr1 | 19.02291 | 1.10067 | 0.552616 | 1.991746 | 0.023199 |
| 12203 | 13436 | Dnmt3b | 7.114226 | -1.39438 | 0.700272 | -1.9912 | 0.02323 |
| 2120 | 368203 | Gm5136 | 11.54503 | 1.334666 | 0.670978 | 1.989136 | 0.023343 |
| 2573 | 216622 | 4931440F1 | 22.33831 | 0.845516 | 0.425342 | 1.987847 | 0.023414 |
| 213 | 70676 | Gulp1 | 14.22797 | -1.18192 | 0.595087 | -1.98614 | 0.023509 |
| 2996 | 18208 | Ntn1 | 22.29933 | 0.994587 | 0.501139 | 1.984653 | 0.023592 |
| 8730 | 14969 | H2-Eb1 | 25.88848 | 0.859952 | 0.434313 | 1.980029 | 0.02385 |
| 3155 | 76281 | Tax1bp3 | 17.59919 | 0.977564 | 0.493897 | 1.979288 | 0.023892 |
| 3373 | 20308 | Ccl9 | 6.838208 | 1.861586 | 0.941433 | 1.977395 | 0.023998 |
| 16651 | 209032 | Zc3hav1l | 11.41935 | 1.290246 | 0.653065 | 1.975678 | 0.024096 |
| 17106 | 58182 | Prokr1 | 1.33793 | 3.659073 | 1.852298 | 1.975424 | 0.02411 |
| 13384 | 242151 | Kcna10 | 1.511566 | 4.158074 | 2.105509 | 1.974855 | 0.024142 |
| 7801 | 1.01E+08 | 1700007L1 | 3.411625 | -2.66707 | 1.354532 | -1.969 | 0.024477 |
| 9763 | 408198 | Spink7 | 2.381285 | -3.36788 | 1.711377 | -1.96794 | 0.024538 |
| 5126 | 72518 | 2610307P1 | 1.138861 | 3.937709 | 2.003657 | 1.965261 | 0.024692 |
| 19458 | 233826 | Palb2 | 2.878399 | 2.295506 | 1.168884 | 1.963845 | 0.024774 |
| 2925 | 67473 | Slc47a1 | 49.65401 | 0.683508 | 0.348191 | 1.963027 | 0.024822 |
| 19498 | 171504 | Apobr | 4.747938 | 2.090985 | 1.067368 | 1.95901 | 0.025056 |
| 6138 | 78416 | Rnase6 | 1.734205 | -3.52158 | 1.797661 | -1.95898 | 0.025058 |
| 19570 | 233905 | Zfp646 | 31.64642 | 0.631412 | 0.322318 | 1.958973 | 0.025058 |
| 9835 | 73606 | 1700120E1 | 2.562655 | 3.58622 | 1.830948 | 1.958668 | 0.025076 |
| 1907 | 208677 | Creb3l3 | 8.760065 | -1.58326 | 0.809191 | -1.9566 | 0.025197 |
| 16470 | 14699 | Gngt1 | 1.835107 | 3.914189 | 2.002231 | 1.954914 | 0.025297 |
| 6840 | 19296 | Pvt1 | 17.96774 | 0.956372 | 0.489648 | 1.953185 | 0.025399 |
| 12949 | 20319 | Sfrp2 | 11.19581 | 1.408051 | 0.721873 | 1.950552 | 0.025555 |
| 3218 | 380713 | Scarf1 | 10.82384 | -1.33322 | 0.683935 | -1.94934 | 0.025627 |
| 2053 | 12905 | Cradd | 38.37056 | 0.740625 | 0.380728 | 1.945284 | 0.02587 |
| 18853 | 269952 | Gdpgp1 | 44.88636 | 0.634073 | 0.32641 | 1.942564 | 0.026034 |
| 20284 | 83984 | Tssk6 | 2.264386 | -3.13698 | 1.615178 | -1.94219 | 0.026057 |
| 9139 | 18188 | Nrtn | 7.248882 | -1.24269 | 0.640039 | -1.94158 | 0.026094 |
| 2442 | 140703 | Emid1 | 33.62197 | 0.743291 | 0.383459 | 1.938385 | 0.026288 |
| 19811 | 78914 | Nadsyn1 | 20.29672 | 0.846874 | 0.437067 | 1.93763 | 0.026334 |
| 3851 | 70207 | Taco1 | 16.61558 | 0.899381 | 0.464696 | 1.935417 | 0.02647 |
| 9220 | 225004 | BC027072 | 2.599299 | 3.656295 | 1.889353 | 1.93521 | 0.026482 |
| 20254 | 74419 | Tktl2 | 4.032814 | 2.460776 | 1.273888 | 1.931705 | 0.026698 |
| 4456 | 380765 | D830013C | 1.514972 | 3.605195 | 1.872176 | 1.925671 | 0.027073 |
| 13756 | 208890 | Slc26a7 | 10.62599 | 1.061795 | 0.551642 | 1.924791 | 0.027128 |
| 19942 | 11601 | Angpt2 | 9.992963 | -1.27655 | 0.666028 | -1.91667 | 0.02764 |
| 15966 | 77846 | B230112J1 | 2.309482 | 2.542822 | 1.328171 | 1.914529 | 0.027776 |
| 21697 | 244911 | C2cd4a | 6.603803 | -1.62022 | 0.847348 | -1.91211 | 0.027931 |
| 4640 | 22095 | Tshr | 1.321328 | 3.84263 | 2.010828 | 1.910969 | 0.028004 |
| 7955 | 320712 | Abi3bp | 32.56958 | 0.811247 | 0.425249 | 1.907697 | 0.028215 |
| 21960 | 68553 | Col6a4 | 9.786382 | -1.3236 | 0.694348 | -1.90625 | 0.028309 |
| 9602 | 93707 | Pcdhgc4 | 1.867641 | -3.47866 | 1.825623 | -1.90546 | 0.02836 |
| 20221 | 78353 | 2500002B1 | 10.63218 | -1.14535 | 0.602519 | -1.90094 | 0.028655 |
| 17169 | 69798 | 1810044D | 5.188259 | -1.71421 | 0.904366 | -1.89549 | 0.029014 |
| 13638 | 1E+08 | Gm10636 | 1.40803 | 3.092381 | 1.632146 | 1.894672 | 0.029068 |
| 19738 | 66985 | Rassf7 | 5.626333 | 1.569478 | 0.829213 | 1.892732 | 0.029197 |
| 6415 | 75016 | 4930480K2 | 18.16282 | 0.946168 | 0.499933 | 1.892589 | 0.029206 |
| 19571 | 330657 | Prss53 | 33.04001 | 0.682623 | 0.361445 | 1.888596 | 0.029473 |
| 7672 | 13544 | Dvl3 | 10.13659 | 1.338405 | 0.709167 | 1.887292 | 0.029561 |
| 8876 | 240095 | H2-M5 | 1.405177 | -3.82411 | 2.027132 | -1.88646 | 0.029616 |
| 3865 | 15896 | Icam2 | 23.18108 | 0.745578 | 0.395249 | 1.886353 | 0.029624 |
| 6447 | 668489 | Gm9199 | 6.113354 | -1.43486 | 0.760729 | -1.88617 | 0.029636 |
| 22222 | 22646 | Zfp105 | 12.72014 | 1.058172 | 0.561585 | 1.88426 | 0.029765 |
| 21499 | 75311 | 4930550C | 8.294217 | 1.409728 | 0.749431 | 1.881066 | 0.029981 |
| 13490 | 29815 | Bcar3 | 42.87647 | 0.612842 | 0.325908 | 1.880416 | 0.030026 |
| 9736 | 16149 | Cd74 | 15.80304 | 1.12532 | 0.598654 | 1.879751 | 0.030071 |
| 17327 | 319734 | Cacna2d4 | 8.647893 | -1.1845 | 0.630891 | -1.8775 | 0.030225 |
| 6521 | 77744 | Bora | 4.964099 | 1.815462 | 0.967023 | 1.877371 | 0.030234 |
| 6764 | 223513 | Abra | 1.886765 | 3.373652 | 1.80017 | 1.874074 | 0.03046 |
| 12023 | 246313 | Prokr2 | 10.6549 | 1.047234 | 0.559838 | 1.870602 | 0.0307 |
| 12187 | 14239 | Foxs1 | 4.053036 | -2.25198 | 1.204698 | -1.86933 | 0.030788 |
| 245 | 78016 | Ccdc150 | 2.736348 | 3.057285 | 1.636155 | 1.868579 | 0.030841 |
| 8775 | 114584 | Clic1 | 38.39608 | 0.608003 | 0.325601 | 1.867327 | 0.030928 |
| 4480 | 210762 | Ppp1r36 | 3.815032 | 2.040935 | 1.093439 | 1.866529 | 0.030984 |
| 1146 | 26909 | Exo1 | 1.454272 | -3.40785 | 1.83171 | -1.86048 | 0.031409 |
| 19386 | 12310 | Calca | 22.06194 | 0.905046 | 0.486464 | 1.860461 | 0.03141 |

| | | | | | | | |
|---|---|---|---|---|---|---|---|
| 22902 | 80903 | Fgf16 | 2.07184 | -3.67988 | 1.687139 | -2.18114 | 0.014587 |
| 6681 | 432939 | Gm5468 | 29.41393 | 0.748513 | 0.343473 | 2.179248 | 0.014657 |
| 21535 | 665005 | Gm7444 | 1.541419 | -3.63704 | 1.67077 | -2.17687 | 0.014745 |
| 4259 | 24057 | Sh3yl1 | 51.83815 | 0.714917 | 0.328919 | 2.173538 | 0.01487 |
| 7064 | 1E+08 | Snord83b | 1.377419 | -4.10732 | 1.890499 | -2.17261 | 0.014905 |
| 10114 | 75291 | Zbtb3 | 16.42586 | 1.013686 | 0.466897 | 2.171114 | 0.014961 |
| 18049 | 11812 | Apoc1 | 9.569875 | -1.52259 | 0.702049 | -2.16878 | 0.01505 |
| 18590 | 75736 | Bcl2l12 | 10.65712 | -1.44203 | 0.66502 | -2.16841 | 0.015064 |
| 3411 | 21385 | Tbx2 | 11.67721 | -1.23072 | 0.567609 | -2.16825 | 0.01507 |
| 1830 | 83554 | Fstl3 | 67.60915 | 0.603001 | 0.278594 | 2.164445 | 0.015215 |
| 7334 | 11830 | Aqp5 | 2.309184 | -3.7527 | 1.733895 | -2.16432 | 0.01522 |
| 5972 | 1.01E+08 | 3425401B1 | 1.830771 | -4.13791 | 1.915186 | -2.16058 | 0.015364 |
| 9397 | 73677 | Psma8 | 1.980356 | 3.634741 | 1.697269 | 2.141523 | 0.016116 |
| 11625 | 329470 | Accs | 9.982718 | -1.30513 | 0.609642 | -2.14081 | 0.016145 |
| 8716 | 12815 | Col11a2 | 15.89075 | -1.16814 | 0.546492 | -2.13752 | 0.016278 |
| 21672 | 208084 | Pif1 | 5.657664 | 1.883648 | 0.881914 | 2.135864 | 0.016345 |
| 14419 | 70603 | Mutyh | 13.45906 | 1.264532 | 0.592334 | 2.13483 | 0.016387 |
| 11798 | 76464 | Casc5 | 2.584448 | 3.329975 | 1.560676 | 2.133675 | 0.016435 |
| 2310 | 211389 | Suox | 41.05969 | 0.669662 | 0.313927 | 2.133177 | 0.016455 |
| 14457 | 230678 | Tmem125 | 7.83556 | -1.45099 | 0.680579 | -2.132 | 0.016504 |
| 18674 | 21990 | Tph1 | 3.317138 | -2.44929 | 1.1507 | -2.12852 | 0.016647 |
| 20282 | 68709 | Cilp2 | 41.71155 | 0.66238 | 0.311679 | 2.125202 | 0.016785 |
| 12821 | 17116 | Mab21l1 | 6.506691 | 1.439092 | 0.678447 | 2.121156 | 0.016954 |
| 1541 | 12484 | Cd24a | 42.84421 | 0.773709 | 0.364911 | 2.120266 | 0.016992 |
| 1831 | 73106 | Prss57 | 81.73248 | 0.591201 | 0.279102 | 2.118226 | 0.017078 |
| 3152 | 14841 | Gsg2 | 1.727448 | -3.8297 | 1.810896 | -2.11481 | 0.017223 |
| 10589 | 1E+08 | AA387883 | 20.93013 | 0.825135 | 0.390287 | 2.114177 | 0.01725 |
| 12318 | 18803 | Plcg1 | 49.84038 | 0.680308 | 0.323533 | 2.102746 | 0.017744 |
| 10897 | 13166 | Dbh | 4.617592 | -2.03677 | 0.97434 | -2.09041 | 0.01829 |
| 773 | 71884 | Chit1 | 6.103391 | 2.339662 | 1.119786 | 2.089382 | 0.018337 |
| 4600 | 68737 | Angel1 | 18.16871 | -1.03391 | 0.494919 | -2.08905 | 0.018351 |
| 21918 | 213208 | Il20rb | 4.136356 | -2.91137 | 1.397796 | -2.08283 | 0.018633 |
| 23218 | 170743 | Tlr7 | 15.75965 | 1.024986 | 0.493802 | 2.075704 | 0.018961 |
| 2987 | 17885 | Myh8 | 9.618942 | 1.353133 | 0.65218 | 2.074784 | 0.019003 |
| 14074 | 1E+08 | Mup10 | 2.730715 | -3.1035 | 1.496398 | -2.07398 | 0.01904 |
| 4158 | 238037 | BC068281 | 41.34278 | 0.655531 | 0.316286 | 2.072591 | 0.019105 |
| 15802 | 330149 | Hfm1 | 25.73763 | 0.924922 | 0.446302 | 2.072413 | 0.019113 |
| 11809 | 1E+08 | Gm14207 | 1.706136 | -3.71434 | 1.794691 | -2.06963 | 0.019244 |
| 21759 | 12160 | Bmp5 | 1.661893 | 4.046776 | 1.956952 | 2.067898 | 0.019325 |
| 12427 | 1E+08 | Snord12 | 1.147311 | 4.244667 | 2.054848 | 2.065684 | 0.019429 |
| 9624 | 383348 | Kctd16 | 3.933013 | -2.24921 | 1.089069 | -2.06526 | 0.019449 |
| 4052 | 12416 | Cbx2 | 13.73004 | 1.13112 | 0.54868 | 2.061528 | 0.019626 |
| 1369 | 237300 | Gm4922 | 2.244319 | 3.243291 | 1.574932 | 2.059322 | 0.019732 |
| 8308 | 213389 | Prdm9 | 14.48382 | -1.08544 | 0.527139 | -2.05911 | 0.019742 |
| 2385 | 11923 | Neurod4 | 4.235841 | -1.86548 | 0.908482 | -2.05341 | 0.020017 |
| 10610 | 77594 | 4930552P1 | 2.250788 | 2.940767 | 1.435027 | 2.049276 | 0.020218 |
| 18404 | 66438 | Hamp2 | 1.54484 | -4.10464 | 2.0033 | -2.04894 | 0.020234 |
| 8723 | 16912 | Psmb9 | 11.28924 | -1.28782 | 0.629222 | -2.04668 | 0.020345 |
| 12134 | 114889 | Vsx1 | 2.171182 | 3.252677 | 1.590063 | 2.045627 | 0.020397 |
| 16521 | 21753 | Tes | 30.58809 | 0.881038 | 0.432285 | 2.038096 | 0.02077 |
| 1340 | 74732 | Stx11 | 1.58833 | 4.29045 | 2.10752 | 2.035782 | 0.020886 |
| 20468 | 15936 | Ier2 | 21.28923 | -1.02054 | 0.501526 | -2.03486 | 0.020932 |
| 1539 | 331623 | Bend3 | 17.67384 | -1.05812 | 0.519998 | -2.03485 | 0.020933 |
| 5147 | 66222 | Serpinb1a | 30.30422 | 0.806005 | 0.396623 | 2.032169 | 0.021068 |
| 5903 | 71106 | 4933413J0 | 1.995445 | 3.724878 | 1.841482 | 2.022761 | 0.021549 |
| 10392 | 14102 | Fas | 6.00344 | -1.64118 | 0.811555 | -2.02226 | 0.021575 |
| 18977 | 670727 | Gm15412 | 2.556995 | 3.070664 | 1.519139 | 2.021319 | 0.021623 |
| 13571 | 67547 | Slc39a8 | 12.91428 | -1.02506 | 0.508077 | -2.01754 | 0.02182 |
| 17087 | 381792 | 2310040G | 16.37966 | -1.09456 | 0.543209 | -2.01499 | 0.021953 |
| 10813 | 97031 | Tprn | 43.61629 | 0.629403 | 0.313312 | 2.008872 | 0.022275 |
| 1634 | 237362 | Npffr1 | 2.936167 | 2.548736 | 1.269763 | 2.007253 | 0.022361 |
| 1488 | 319415 | Hs3st5 | 34.09036 | 0.717912 | 0.357984 | 2.005427 | 0.022459 |
| 6382 | 69303 | 1700001G | 1.746697 | -3.57385 | 1.788754 | -1.99795 | 0.022861 |
| 13730 | 320152 | 4930412C1 | 9.224505 | -1.33021 | 0.665969 | -1.99741 | 0.022891 |
| 18962 | 70054 | Ccdc89 | 1.999543 | 3.721622 | 1.865608 | 1.994857 | 0.023029 |
| 21639 | 17130 | Smad6 | 11.44975 | -1.1288 | 0.566301 | -1.99328 | 0.023115 |
| 2634 | 319767 | Atp10b | 4.189394 | 2.545617 | 1.278306 | 1.991399 | 0.023219 |
| 17268 | 58991 | Ghrl | 1.50031 | 4.168036 | 2.096026 | 1.988543 | 0.023376 |
| 2065 | 17022 | Lum | 5.450698 | -2.36047 | 1.187818 | -1.98723 | 0.023448 |
| 9142 | 19725 | Rfx2 | 11.06237 | -1.17791 | 0.59291 | -1.98665 | 0.02348 |
| 4456 | 380765 | D830013C | 1.514972 | 3.705623 | 1.865475 | 1.986424 | 0.023493 |
| 8847 . | | C920025E | 1.079624 | -3.61348 | 1.821617 | -1.98367 | 0.023646 |
| 16720 | 13835 | Epha1 | 6.239891 | -1.50994 | 0.763121 | -1.97863 | 0.023929 |
| 15149 | 70920 | 4921511H | 7.35052 | 1.49926 | 0.757993 | 1.977933 | 0.023968 |
| 986 | 433374 | Gm20743 | 1.313827 | -3.91938 | 1.983927 | -1.97557 | 0.024102 |
| 10393 | 12642 | Ch25h | 4.011616 | -2.84517 | 1.440995 | -1.97445 | 0.024166 |
| 16320 | 56522 | Papolb | 9.313491 | 1.361921 | 0.690307 | 1.972922 | 0.024252 |
| 2615 | 574403 | Fam196b | 9.778141 | -1.43736 | 0.729609 | -1.97005 | 0.024417 |
| 20004 | 13214 | Defb1 | 6.358394 | 1.753724 | 0.891523 | 1.96711 | 0.024585 |
| 16949 | 14080 | Fabp1 | 1.564175 | -3.30453 | 1.687749 | -1.95795 | 0.025118 |
| 7202 | 29857 | Mapk12 | 29.55852 | 0.835404 | 0.426721 | 1.957731 | 0.025131 |
| 22416 | 11609 | Agtr2 | 1.105112 | -3.95396 | 2.020514 | -1.95691 | 0.025179 |
| 4707 | 20703 | Serpina1d | 4.152742 | -2.27584 | 1.164837 | -1.95378 | 0.025363 |
| 19027 | 69121 | Chrdl2 | 4.70602 | -2.01154 | 1.031698 | -1.94974 | 0.025604 |
| 15400 | 76710 | 2210406O | 36.93341 | 0.849902 | 0.435991 | 1.949354 | 0.025627 |
| 10614 | 12369 | Casp7 | 11.15112 | -1.27175 | 0.653301 | -1.94665 | 0.025788 |
| 2707 | 14257 | Flt4 | 16.44062 | -1.23769 | 0.636721 | -1.94385 | 0.025957 |
| 11976 | 1E+08 | Snord57 | 1.474935 | -3.6586 | 1.882691 | -1.94328 | 0.025991 |
| 20549 | 13884 | Ces1c | 2.307895 | -2.80696 | 1.446116 | -1.94103 | 0.026127 |

| | | | | | | | |
|---|---|---|---|---|---|---|---|
| 17708 | 320204 | Mettl20 | 5.208256 | 1.600753 | 0.861078 | 1.859011 | 0.031513 |
| 14990 | 56485 | Slc2a5 | 48.98401 | 0.597936 | 0.321743 | 1.858427 | 0.031554 |
| 20875 | 1E+08 | Snord68 | 1.386913 | -3.75162 | 2.019637 | -1.85757 | 0.031615 |
| 13676 | 1E+08 | Snord45c | 2.32292 | 3.040486 | 1.639894 | 1.854075 | 0.031864 |
| 1112 | 236312 | Pyhin1 | 1.491201 | -3.38884 | 1.828714 | -1.85313 | 0.031932 |
| 204 | 78896 | 1500015O | 14.52325 | 1.095636 | 0.591369 | 1.85271 | 0.031962 |
| 18969 | 73845 | Ankrd42 | 31.30321 | 0.721554 | 0.389598 | 1.852048 | 0.032009 |
| 5989 | 11752 | Anxa8 | 1.853061 | 3.352026 | 1.812134 | 1.849767 | 0.032174 |
| 21509 | 13075 | Cyp19a1 | 1.087325 | 3.836337 | 2.076619 | 1.847396 | 0.032345 |
| 491 | 1E+08 | Snora75 | 1.119212 | -4.17135 | 2.258814 | -1.8467 | 0.032395 |
| 4858 | 1E+08 | Gm10421 | 3.231624 | 2.392166 | 1.297678 | 1.84342 | 0.032634 |
| 21407 | 14012 | Mpzl2 | 33.86342 | 0.870925 | 0.472479 | 1.843309 | 0.032642 |
| 21526 | 108015 | Chrnb4 | 1.359306 | -3.11642 | 1.691101 | -1.84284 | 0.032676 |
| 9219 | 320159 | Fam179a | 13.29572 | 0.949223 | 0.515279 | 1.842151 | 0.032727 |
| 15561 | 231290 | Slc10a4 | 2.86448 | 3.262112 | 1.77088 | 1.842085 | 0.032731 |
| 20770 | 56773 | Chst5 | 5.237402 | -2.03545 | 1.108774 | -1.83576 | 0.033196 |
| 13531 | 20132 | Rrh | 1.219836 | 4.035759 | 2.198672 | 1.835544 | 0.033213 |
| 3376 | 20302 | Ccl3 | 1.256043 | 3.539827 | 1.928563 | 1.835474 | 0.033218 |
| 21607 | 23958 | Nr2e3 | 1.250709 | 4.268692 | 2.327185 | 1.834273 | 0.033307 |
| 8709 | 630499 | H2-K2 | 3.682278 | -1.89724 | 1.035417 | -1.83234 | 0.03345 |
| 10612 | 226243 | Habp2 | 1.20956 | 3.489992 | 1.904678 | 1.832326 | 0.033451 |
| 3299 | 16859 | Lgals9 | 34.9231 | 0.648115 | 0.353972 | 1.830978 | 0.033552 |
| 5231 | 1.01E+08 | A330076C | 2.341148 | -2.46249 | 1.348723 | -1.82579 | 0.033941 |
| 2202 | 1.01E+08 | Gm15910 | 11.72828 | 0.927066 | 0.508583 | 1.822841 | 0.034164 |
| 923 | 89867 | Sec16b | 8.584429 | -1.17144 | 0.643062 | -1.82165 | 0.034254 |
| 4214 | 236366 | 5730507C( | 3.156041 | -2.63451 | 1.450474 | -1.81631 | 0.034662 |
| 13529 | 319224 | 6330410L2 | 1.100719 | 4.186241 | 2.305071 | 1.816101 | 0.034677 |
| 9600 | 93724 | Pcdhga12 | 8.197964 | -1.43131 | 0.788654 | -1.81488 | 0.034771 |
| 8841 | 15040 | H2-T23 | 7.185141 | -1.25425 | 0.691984 | -1.81254 | 0.034951 |
| 18636 | 66065 | Hsd17b14 | 1.062889 | 3.785794 | 2.088735 | 1.812481 | 0.034956 |
| 379 | 109198 | 6030407O | 2.28915 | -3.12631 | 1.725462 | -1.81187 | 0.035003 |
| 4074 | 1E+08 | 2810410L2 | 8.651257 | 1.331235 | 0.735843 | 1.80913 | 0.035215 |
| 9014 | 71461 | Ptk7 | 23.96996 | 1.035034 | 0.572545 | 1.807776 | 0.035321 |
| 20081 | 75767 | Rab11fip1 | 13.92837 | 1.04616 | 0.579217 | 1.806162 | 0.035447 |
| 4519 | 217682 | Plekhd1 | 26.318 | 0.774863 | 0.429836 | 1.802695 | 0.035718 |
| 14155 | 790913 | Gm11413 | 2.421222 | -2.82503 | 1.572386 | -1.79665 | 0.036195 |
| 2016 | 216227 | Slc17a8 | 10.01468 | -1.22584 | 0.683689 | -1.79297 | 0.036489 |
| 20122 | 1.01E+08 | Gm16793 | 1.493702 | -3.92404 | 2.189012 | -1.79261 | 0.036518 |
| 1063 | 226654 | Tstd1 | 11.98722 | 1.013719 | 0.565741 | 1.791842 | 0.036579 |
| 6345 | 382913 | Neil2 | 28.98132 | 0.67209 | 0.375092 | 1.791799 | 0.036583 |
| 15883 | 12960 | Crybb1 | 33.22108 | 0.92492 | 0.516274 | 1.79153 | 0.036604 |
| 17375 | 269799 | Clec4a1 | 5.244727 | -1.46277 | 0.816997 | -1.79042 | 0.036693 |
| 4912 | 171211 | Edaradd | 6.401403 | 1.312263 | 0.73432 | 1.787046 | 0.036965 |
| 5742 | 109700 | Itga1 | 14.01651 | 1.063455 | 0.595107 | 1.786997 | 0.036969 |
| 14582 | 100317 | AU040320 | 34.10618 | 0.806383 | 0.451422 | 1.786316 | 0.037024 |
| 17378 | 69810 | Clec4b1 | 1.360874 | 3.622751 | 2.028853 | 1.785615 | 0.037081 |
| 17622 | 277898 | Slc15a5 | 1.608091 | 2.936035 | 1.648739 | 1.780776 | 0.037475 |
| 371 | 319695 | Ankar | 1.225113 | -3.36536 | 1.889962 | -1.78065 | 0.037485 |
| 822 | 1.01E+08 | Platr22 | 1.282967 | 3.626123 | 2.037718 | 1.779502 | 0.037579 |
| 12284 | 19650 | Rbl1 | 8.179848 | 1.214173 | 0.683167 | 1.777271 | 0.037762 |
| 7243 | 77248 | 9430014N | 4.532315 | 1.710418 | 0.963787 | 1.774686 | 0.037975 |
| 10235 | 107321 | Lpxn | 9.266785 | -1.13326 | 0.638574 | -1.77467 | 0.037976 |
| 22638 | 207854 | Fmr1nb | 1.33032 | 3.640585 | 2.052017 | 1.77415 | 0.038019 |
| 17041 | 574519 | Vax2os | 1.284241 | 4.4079 | 2.485853 | 1.773194 | 0.038098 |
| 869 | 63913 | Fam129a | 5.781779 | 1.574228 | 0.888004 | 1.772772 | 0.038133 |
| 15999 | 170756 | Slc8b1 | 11.04102 | 0.962271 | 0.543566 | 1.770292 | 0.038339 |
| 15345 | 665270 | Plb1 | 10.64981 | -1.08319 | 0.612688 | -1.76793 | 0.038537 |
| 6932 | 223650 | Eppk1 | 4.686743 | -1.51671 | 0.858819 | -1.76604 | 0.038694 |
| 16479 | 18979 | Pon1 | 1.055387 | 4.00503 | 2.268658 | 1.765374 | 0.03875 |
| 4445 | 78257 | Lrrc9 | 1.058281 | 3.769055 | 2.134992 | 1.765372 | 0.038751 |
| 16857 | 21743 | Inmt | 11.63526 | -1.05134 | 0.595601 | -1.76518 | 0.038767 |
| 17628 | 50540 | Igbp1b | 2.294452 | -2.52564 | 1.431038 | -1.7649 | 0.03879 |
| 4014 | 193217 | BC018473 | 2.24113 | 2.775672 | 1.574162 | 1.763269 | 0.038928 |
| 11625 | 329470 | Accs | 9.982718 | -1.06036 | 0.601823 | -1.76192 | 0.039042 |
| 20680 | 109660 | Ctrl | 7.478883 | -1.33425 | 0.758892 | -1.75816 | 0.03936 |
| 9561 | 93875 | Pcdhb4 | 12.8619 | -1.10938 | 0.631035 | -1.75803 | 0.039372 |
| 21441 | 78252 | Nxpe2 | 9.980952 | -1.35345 | 0.771586 | -1.75412 | 0.039705 |
| 3523 | 18053 | Ngfr | 9.868697 | 1.266135 | 0.722775 | 1.75177 | 0.039907 |
| 21759 | 12160 | Bmp5 | 1.661893 | 3.469789 | 1.980763 | 1.751744 | 0.039909 |
| 13174 | 19885 | Rorc | 2.751302 | -2.98065 | 1.704797 | -1.74839 | 0.040198 |
| 7350 | 668178 | Mettl7a3 | 1.599622 | 3.313349 | 1.896045 | 1.747506 | 0.040275 |
| 6604 | 69393 | 1700024B' | 1.720045 | 3.177514 | 1.819387 | 1.746475 | 0.040364 |
| 12014 | 71995 | Erv3 | 1.180062 | 3.093016 | 1.771459 | 1.746027 | 0.040403 |
| 20722 | 56523 | Pmfbp1 | 2.317174 | 2.72987 | 1.570953 | 1.737716 | 0.04113 |
| 5719 | 13206 | Ddx4 | 22.35815 | 0.740482 | 0.426128 | 1.737699 | 0.041132 |
| 2185 | 69181 | Dyrk2 | 8.825764 | -1.10426 | 0.636165 | -1.73581 | 0.041298 |
| 1339 | 320273 | B230208H | 3.840542 | -2.07352 | 1.194825 | -1.73542 | 0.041333 |
| 13089 | 18160 | Npr1 | 10.36952 | -1.33007 | 0.767308 | -1.73342 | 0.04151 |
| 19726 | 68713 | Ifitm1 | 21.79726 | 0.715031 | 0.412524 | 1.73331 | 0.04152 |
| 4770 | 13386 | Dlk1 | 193.0518 | 0.625991 | 0.36119 | 1.733134 | 0.041536 |
| 4868 | 238455 | Macc1 | 2.720216 | -2.38306 | 1.375562 | -1.73242 | 0.041599 |
| 7018 | 104445 | Cdc42ep1 | 25.8472 | 0.635482 | 0.367129 | 1.730952 | 0.04173 |
| 1391 | 237320 | Aldh8a1 | 1.146797 | -4.11057 | 2.375235 | -1.73059 | 0.041762 |
| 9189 | 320209 | Ddx11 | 4.168409 | 1.611604 | 0.931703 | 1.72974 | 0.041838 |
| 21907 | 272636 | Esyt3 | 41.54644 | 0.607745 | 0.3515 | 1.729004 | 0.041904 |
| 11836 | 70933 | 4931402G | 1.474971 | -3.44911 | 2.001329 | -1.72341 | 0.042407 |
| 5695 | 1E+08 | 3830408C2 | 4.054615 | -1.82333 | 1.058035 | -1.72331 | 0.042416 |
| 21623 | 330958 | A730043L( | 5.987054 | 1.372322 | 0.796758 | 1.722382 | 0.0425 |
| 14716 | 23833 | Cd52 | 20.98763 | 0.682291 | 0.396275 | 1.721761 | 0.042556 |

| | | | | | | | |
|---|---|---|---|---|---|---|---|
| 13103 | 20201 | S100a8 | 8.847814 | -1.55322 | 0.801005 | -1.93909 | 0.026245 |
| 6331 | 239134 | Gucy1b2 | 2.120863 | 3.092776 | 1.597753 | 1.935703 | 0.026452 |
| 4078 | 238021 | Fscn2 | 2.049871 | 3.418518 | 1.766251 | 1.935466 | 0.026467 |
| 20585 | 54672 | Adgrg3 | 2.611121 | -2.9134 | 1.507097 | -1.93312 | 0.026611 |
| 14341 | 108802 | Calr4 | 3.626328 | 2.099165 | 1.086477 | 1.932084 | 0.026675 |
| 5480 | 408058 | BC048507 | 4.002328 | -2.07435 | 1.073916 | -1.93158 | 0.026706 |
| 1028 | 16589 | Uhmk1 | 56.97986 | 0.604473 | 0.313049 | 1.930923 | 0.026746 |
| 15659 | 68473 | Mob1b | 20.00891 | 1.010912 | 0.523935 | 1.929461 | 0.026837 |
| 15347 | 22612 | Yes1 | 33.72024 | 0.640147 | 0.331861 | 1.928959 | 0.026868 |
| 6539 | 70898 | Slain1os | 2.125583 | -3.29799 | 1.710415 | -1.92818 | 0.026916 |
| 18708 | 108961 | E2f8 | 1.166503 | -3.9135 | 2.030088 | -1.92775 | 0.026943 |
| 4932 | 71071 | 4933412O | 38.79957 | 0.692942 | 0.359925 | 1.925242 | 0.0271 |
| 2725 | 214779 | Zfp879 | 14.82376 | 0.957469 | 0.498432 | 1.920961 | 0.027368 |
| 16607 | 619665 | Klf14 | 3.484747 | 2.283919 | 1.189619 | 1.919874 | 0.027437 |
| 16358 | 1.01E+08 | Gm15708 | 13.36873 | -1.32054 | 0.687911 | -1.91963 | 0.027452 |
| 15504 | 77382 | C030018K | 9.504023 | 1.336168 | 0.69643 | 1.918596 | 0.027518 |
| 20662 | 11604 | Agrp | 1.801838 | -3.50299 | 1.826236 | -1.91815 | 0.027546 |
| 15384 | 54426 | Hgfac | 1.475422 | 3.820215 | 1.992869 | 1.916942 | 0.027623 |
| 4706 | 20701 | Serpina1b | 4.583385 | -2.12369 | 1.10828 | -1.91621 | 0.027669 |
| 7779 | 69576 | Smco1 | 4.38624 | -1.6936 | 0.884727 | -1.91427 | 0.027793 |
| 3523 | 18053 | Ngfr | 9.868697 | 1.376438 | 0.719355 | 1.913432 | 0.027846 |
| 6246 | 57260 | Ltb4r2 | 1.312552 | -4.36807 | 2.283771 | -1.91266 | 0.027896 |
| 16242 | 751865 | Sap25 | 1.393931 | 4.131118 | 2.164101 | 1.90893 | 0.028136 |
| 8676 | 1E+08 | Olfr55 | 4.266635 | 1.881547 | 0.988742 | 1.90297 | 0.028522 |
| 14681 | 19204 | Ptafr | 2.176002 | 3.12836 | 1.644943 | 1.901804 | 0.028598 |
| 12408 | 228876 | Zfp334 | 55.85801 | 0.587893 | 0.309197 | 1.901358 | 0.028628 |
| 16864 | 1E+08 | Gm3279 | 6.141671 | 1.863075 | 0.979935 | 1.901223 | 0.028636 |
| 17590 | 381823 | Apold1 | 33.51634 | 0.702736 | 0.370347 | 1.897506 | 0.028881 |
| 6617 | 239319 | Card6 | 8.875733 | 1.152512 | 0.607666 | 1.896621 | 0.028939 |
| 20756 | 319518 | Pdpr | 12.39955 | 1.139192 | 0.600801 | 1.896122 | 0.028972 |
| 5495 | 271127 | Adamts16 | 20.4038 | 0.946392 | 0.499173 | 1.895921 | 0.028985 |
| 12764 | 20873 | Plk4 | 35.00721 | 0.714624 | 0.377178 | 1.894661 | 0.029069 |
| 1664 | 67307 | Pbld2 | 6.393714 | 1.688539 | 0.89155 | 1.893938 | 0.029117 |
| 3870 | 380732 | Milr1 | 5.669348 | 1.917305 | 1.012887 | 1.89291 | 0.029185 |
| 4708 | 20700 | Serpina1a | 2.583496 | -2.97453 | 1.575835 | -1.88759 | 0.02954 |
| 4725 | 20717 | Serpina3m | 1.800585 | -3.30722 | 1.752714 | -1.88691 | 0.029586 |
| 8015 | 13837 | Epha3 | 38.33974 | 0.672702 | 0.356733 | 1.885728 | 0.029666 |
| 3299 | 16859 | Lgals9 | 34.9231 | 0.665288 | 0.352997 | 1.884688 | 0.029736 |
| 4244 | 71693 | Colec11 | 7.058527 | -1.67963 | 0.891575 | -1.88389 | 0.02979 |
| 5020 | 171250 | Vmn1r206 | 1.277514 | 4.208174 | 2.234442 | 1.883322 | 0.029828 |
| 491 | 1E+08 | Snora75 | 1.119212 | -4.23226 | 2.258814 | -1.87366 | 0.030488 |
| 7955 | 320712 | Abi3bp | 32.56958 | 0.795745 | 0.424765 | 1.873377 | 0.030508 |
| 7243 | 77248 | 9430014N | 4.532315 | 1.795726 | 0.95922 | 1.872068 | 0.030599 |
| 2232 | 13115 | Cyp27b1 | 2.268676 | -2.91409 | 1.558477 | -1.86983 | 0.030754 |
| 18646 | 243967 | Ntn5 | 27.3413 | 0.730067 | 0.390919 | 1.867564 | 0.030911 |
| 15824 | 433931 | Pigg | 10.47836 | -1.00647 | 0.539301 | -1.86625 | 0.031003 |
| 14582 | 100317 | AU040320 | 34.10618 | 0.84065 | 0.450494 | 1.866064 | 0.031016 |
| 9593 | 93702 | Pcdhgb5 | 15.02573 | 0.841538 | 0.45124 | 1.864944 | 0.031095 |
| 21499 | 75311 | 4930550C | 8.294217 | 1.39563 | 0.748393 | 1.864837 | 0.031102 |
| 1263 | 98736 | 1700034H | 14.97063 | -1.00338 | 0.538487 | -1.86334 | 0.031207 |
| 1524 | 75973 | Ccdc162 | 4.103663 | 2.523455 | 1.355227 | 1.862017 | 0.0313 |
| 5932 | 16427 | Itih4 | 1.515802 | -3.22243 | 1.731938 | -1.86059 | 0.031401 |
| 20481 | 13423 | Dnase2a | 18.7329 | 0.896488 | 0.482131 | 1.859429 | 0.031483 |
| 14850 | 329972 | Spata21 | 2.42069 | -2.69637 | 1.450834 | -1.8585 | 0.031549 |
| 1661 | 327762 | Dna2 | 6.844068 | 1.55285 | 0.836035 | 1.8574 | 0.031627 |
| 15390 | 242939 | Cpz | 1.605618 | -3.76879 | 2.02932 | -1.85717 | 0.031644 |
| 13106 | 619677 | 4930529C( | 1.162136 | 4.26901 | 2.299636 | 1.856385 | 0.031699 |
| 19286 | 320635 | Cyb5r2 | 1.617115 | 3.165753 | 1.708057 | 1.853424 | 0.031911 |
| 14990 | 56485 | Slc2a5 | 48.98401 | 0.594476 | 0.321179 | 1.850914 | 0.032091 |
| 13490 | 29815 | Bcar3 | 42.87647 | 0.60004 | 0.325378 | 1.844129 | 0.032582 |
| 22708 | 83553 | Tktl1 | 1.384942 | -3.29744 | 1.78903 | -1.84315 | 0.032654 |
| 588 | 64095 | Gpr35 | 1.855634 | -3.69836 | 2.00809 | -1.84173 | 0.032757 |
| 678 | 68428 | Steap3 | 17.65003 | 1.017254 | 0.552493 | 1.841206 | 0.032796 |
| 2573 | 216622 | 4931440F1 | 22.33831 | 0.780517 | 0.425433 | 1.834639 | 0.03328 |
| 18645 | 56546 | Sec1 | 7.513239 | 1.346064 | 0.73373 | 1.834551 | 0.033286 |
| 14086 | 17842 | Mup3 | 4.19042 | -2.4185 | 1.318682 | -1.83402 | 0.033325 |
| 3795 | . | Gm1564 | 3.995067 | 2.768181 | 1.50974 | 1.833548 | 0.033361 |
| 7372 | 11479 | Acvr1b | 11.16645 | -1.1263 | 0.615221 | -1.83073 | 0.033571 |
| 22222 | 22646 | Zfp105 | 12.72014 | 1.024918 | 0.56104 | 1.826819 | 0.033864 |
| 23163 | 1E+08 | Yy2 | 13.52769 | 1.182442 | 0.648361 | 1.82374 | 0.034096 |
| 1115 | 15951 | Ifi204 | 1.687141 | 3.367319 | 1.848623 | 1.821528 | 0.034263 |
| 8146 | 209195 | Clic6 | 4.783747 | 1.880033 | 1.032238 | 1.821316 | 0.034279 |
| 14249 | 30924 | Angptl3 | 2.810988 | 2.423079 | 1.331635 | 1.819627 | 0.034408 |
| 6321 | 219144 | Arl11 | 3.36421 | 2.27354 | 1.250062 | 1.818742 | 0.034475 |
| 16924 | 70779 | Prdm5 | 7.871333 | -1.2868 | 0.709015 | -1.81492 | 0.034768 |
| 14946 | 28010 | Miip | 37.61957 | 0.598679 | 0.330196 | 1.813099 | 0.034908 |
| 371 | 319695 | Ankar | 1.225113 | -3.42626 | 1.889962 | -1.81287 | 0.034926 |
| 13535 | 12368 | Casp6 | 23.80567 | 0.799049 | 0.440765 | 1.812867 | 0.034926 |
| 3218 | 380713 | Scarf1 | 10.82384 | -1.22917 | 0.678047 | -1.81281 | 0.034931 |
| 1975 | 14202 | Fhl4 | 1.365139 | -3.75343 | 2.073857 | -1.80988 | 0.035157 |
| 1998 | 22172 | Tyms-ps | 2.070004 | -3.02945 | 1.674444 | -1.80923 | 0.035208 |
| 11971 | 241636 | Tgm6 | 1.300034 | -3.67253 | 2.031206 | -1.80806 | 0.035299 |
| 3258 | 19193 | Pipox | 4.293497 | -2.39634 | 1.32633 | -1.80675 | 0.035401 |
| 13707 | 70720 | 6330407A( | 24.65183 | 0.914647 | 0.506598 | 1.805469 | 0.035501 |
| 7633 | 66779 | 4933432I0 | 8.944244 | -1.18505 | 0.656547 | -1.80497 | 0.03554 |
| 12961 | 654362 | Dear1 | 18.94229 | 0.813126 | 0.450723 | 1.804046 | 0.035612 |
| 13946 | 17279 | Melk | 1.46559 | 3.183611 | 1.765894 | 1.802832 | 0.035707 |
| 17820 | . | Gm16532 | 6.123166 | -1.6024 | 0.890157 | -1.80013 | 0.03592 |
| 49 | 226866 | Sbspon | 11.66027 | -1.18728 | 0.660155 | -1.79848 | 0.03605 |

| | | | | | | | |
|---|---|---|---|---|---|---|---|
| 19472 | 210162 | Zkscan2 | 18.80262 | 0.787384 | 0.457593 | 1.720709 | 0.042652 |
| 7911 | 212998 | BC016579 | 2.924793 | -2.93467 | 1.705757 | -1.72045 | 0.042675 |
| 3973 | 140721 | Caskin2 | 27.13392 | 0.845744 | 0.49169 | 1.720077 | 0.042709 |
| 18977 | 670727 | Gm15412 | 2.556995 | 2.638191 | 1.53555 | 1.718076 | 0.042891 |
| 7010 | 16185 | Il2rb | 1.452737 | 3.474051 | 2.022169 | 1.717982 | 0.0429 |
| 133 | 214854 | Neurl3 | 3.313765 | 2.270184 | 1.321821 | 1.717467 | 0.042947 |
| 20269 | 211134 | Lzts1 | 13.31069 | 0.857459 | 0.499375 | 1.717065 | 0.042984 |
| 15788 | 626391 | Zfp951 | 9.418581 | -1.21709 | 0.709165 | -1.71623 | 0.04306 |
| 11210 | 228003 | Klhl41 | 9.379253 | 1.007212 | 0.586877 | 1.716223 | 0.043061 |
| 14332 | 230597 | Zfyve9 | 9.075558 | -1.32655 | 0.773426 | -1.71516 | 0.043158 |
| 10809 | 67859 | Cysrt1 | 1.0192 | 4.074901 | 2.376796 | 1.714452 | 0.043223 |
| 6321 | 219144 | Arl11 | 3.36421 | 2.151141 | 1.255795 | 1.712971 | 0.043359 |
| 14048 | 64817 | Svep1 | 2.785424 | -2.54004 | 1.483297 | -1.71243 | 0.043409 |
| 16010 | 114643 | Oas1c | 5.221318 | 1.544189 | 0.901989 | 1.711982 | 0.04345 |
| 16921 | 243407 | C130060K | 4.786479 | -1.93861 | 1.134429 | -1.70888 | 0.043736 |
| 7613 | 74685 | Lrrc74b | 4.645781 | 1.70095 | 0.995501 | 1.708637 | 0.043759 |
| 3849 | 192775 | Kcnh6 | 1.114157 | -3.83246 | 2.245394 | -1.70681 | 0.043929 |
| 8320 | 14293 | Fpr1 | 2.937317 | -2.70077 | 1.582826 | -1.7063 | 0.043977 |
| 2986 | 17884 | Myh4 | 3.828149 | 1.889252 | 1.108292 | 1.704652 | 0.04413 |
| 12333 | 59091 | Jph2 | 13.97074 | -1.01929 | 0.598344 | -1.70353 | 0.044235 |
| 838 | 214403 | Gm4788 | 0.977332 | -3.39336 | 1.993994 | -1.70179 | 0.044397 |
| 16606 | 116732 | Tsga13 | 1.846476 | -3.14027 | 1.847235 | -1.69998 | 0.044567 |
| 10661 | 633057 | Gm7102 | 1.640737 | -3.17365 | 1.867542 | -1.69937 | 0.044624 |
| 16296 | 231830 | Micall2 | 3.918551 | 1.813829 | 1.06835 | 1.697785 | 0.044774 |
| 18368 | 23900 | Hcst | 6.222308 | -1.24966 | 0.736251 | -1.69733 | 0.044817 |
| 6794 | 18383 | Tnfrsf11b | 3.362544 | -1.75465 | 1.034296 | -1.69647 | 0.044899 |
| 3087 | 11685 | Alox12e | 6.397703 | 1.239609 | 0.730713 | 1.696437 | 0.044902 |
| 18732 | 14407 | Gabrg3 | 2.332572 | -2.20981 | 1.303681 | -1.69506 | 0.045032 |
| 6978 | 17189 | Mb | 1.993755 | 3.43075 | 2.024242 | 1.694832 | 0.045054 |
| 20401 | 71831 | 1700007B | 1.857398 | 2.80417 | 1.657794 | 1.691507 | 0.04537 |
| 18686 | 97402 | C86187 | 5.692828 | -1.32301 | 0.782862 | -1.68997 | 0.045517 |
| 13978 | 74735 | Trim14 | 9.111489 | 1.233422 | 0.730674 | 1.68806 | 0.0457 |
| 11962 | 16176 | Il1b | 6.023361 | 1.576563 | 0.934536 | 1.687 | 0.045802 |
| 14249 | 30924 | Angptl3 | 2.810988 | 2.25934 | 1.339481 | 1.686728 | 0.045828 |
| 11896 | 74702 | 4930517E1 | 3.238575 | -2.30442 | 1.367495 | -1.68514 | 0.045981 |
| 1947 | 408062 | Zfp873 | 7.497176 | -1.28149 | 0.760501 | -1.68506 | 0.045988 |
| 15490 | 319216 | 4932441J0 | 1.218444 | -3.52615 | 2.092819 | -1.68488 | 0.046006 |
| 16314 | 14673 | Gna12 | 1.530085 | -3.6001 | 2.136908 | -1.68472 | 0.046021 |
| 18459 | 243923 | Rgs9bp | 6.452263 | 1.443485 | 0.858434 | 1.681534 | 0.04633 |
| 2385 | 11923 | Neurod4 | 4.235841 | -1.48261 | 0.882097 | -1.68078 | 0.046403 |
| 237 | 69397 | 1700019A | 1.278355 | -3.74604 | 2.231538 | -1.67868 | 0.046607 |
| 13424 | 1E+08 | Scarna2 | 3.222527 | 2.620101 | 1.561143 | 1.678323 | 0.046642 |
| 17672 | . | Ifltd1 | 2.182361 | 2.526513 | 1.505821 | 1.677831 | 0.04669 |
| 14818 | 15565 | Htr6 | 10.32026 | -1.18344 | 0.706156 | -1.67589 | 0.04688 |
| 8818 | 268949 | Dpcr1 | 1.42175 | -3.55043 | 2.120184 | -1.67458 | 0.047008 |
| 17263 | 70979 | Fancd2os | 27.41261 | 0.632916 | 0.37842 | 1.672523 | 0.047211 |
| 3640 | 320864 | Krt26 | 0.908055 | 3.765207 | 2.254207 | 1.670302 | 0.04743 |
| 12862 | 269437 | Plch1 | 21.82548 | 0.622002 | 0.372416 | 1.67018 | 0.047442 |
| 2997 | 320207 | Pik3r5 | 18.83253 | 0.784977 | 0.47088 | 1.667042 | 0.047753 |
| 22144 | 74486 | Osbpl10 | 14.96101 | 0.956489 | 0.573841 | 1.666819 | 0.047775 |
| 15384 | 54426 | Hgfac | 1.475422 | 3.357368 | 2.014403 | 1.666682 | 0.047789 |
| 9121 | 106759 | Ticam1 | 18.52726 | 0.750785 | 0.452019 | 1.660958 | 0.048361 |
| 18049 | 11812 | Apoc1 | 9.569875 | -1.14441 | 0.689033 | -1.66089 | 0.048368 |
| 8867 | 19824 | Trim10 | 1.124957 | -4.07249 | 2.452317 | -1.66067 | 0.04839 |
| 9402 | 71367 | Chst9 | 7.733568 | 1.303569 | 0.785444 | 1.659658 | 0.048492 |
| 6928 | 105732 | Fam83h | 11.6727 | 0.985449 | 0.593844 | 1.659441 | 0.048513 |
| 20117 | 330734 | B930018H | 3.652098 | 1.865656 | 1.125011 | 1.658344 | 0.048624 |
| 3158 | 193034 | Trpv1 | 3.372778 | -2.03096 | 1.225284 | -1.65754 | 0.048705 |
| 15796 | 433926 | Lrrc8b | 2.732143 | 2.463073 | 1.486466 | 1.657 | 0.04876 |
| 21940 | 1.01E+08 | 5830418P | 5.489782 | -1.42265 | 0.859497 | -1.65522 | 0.04894 |
| 18009 | 378430 | Nanos2 | 3.09974 | 2.086311 | 1.263005 | 1.651862 | 0.049281 |
| 19676 | 381933 | 6430531B | 7.104111 | 1.329171 | 0.804902 | 1.651346 | 0.049334 |
| 20657 | 270091 | Lrrc36 | 2.469556 | 2.903614 | 1.759296 | 1.650441 | 0.049426 |
| 561 | 70788 | Klhl30 | 1.226081 | 3.332596 | 2.024348 | 1.646256 | 0.049856 |
| 14558 | 230738 | Zc3h12a | 5.649714 | -1.3716 | 0.833275 | -1.64603 | 0.049879 |

| | | | | | | | |
|---|---|---|---|---|---|---|---|
| 17099 | 52372 | D6Ertd527 | 5.217 | 2.244505 | 1.249008 | 1.79703 | 0.036165 |
| 4778 | 1E+08 | AF357359 | 1.810486 | 3.183174 | 1.772429 | 1.795939 | 0.036252 |
| 4834 | 104759 | Pld4 | 30.67062 | 0.658358 | 0.367079 | 1.793502 | 0.036446 |
| 21794 | 74633 | 4930429F | 8.154282 | 1.637985 | 0.913458 | 1.793171 | 0.036473 |
| 17327 | 319734 | Cacna2d4 | 8.647893 | -1.11949 | 0.625082 | -1.79095 | 0.036651 |
| 1286 | 320400 | Gm16897 | 4.091039 | 1.641177 | 0.916784 | 1.790146 | 0.036715 |
| 2979 | 338369 | Tmem220 | 11.46316 | -1.07216 | 0.599079 | -1.78968 | 0.036753 |
| 3984 | 70450 | Unc13d | 3.641047 | -1.76026 | 0.983791 | -1.78926 | 0.036786 |
| 17103 | 68888 | Gkn3 | 14.54335 | 0.853973 | 0.47759 | 1.788088 | 0.036881 |
| 2657 | 320116 | Fndc9 | 19.64451 | -1.04396 | 0.583845 | -1.78808 | 0.036881 |
| 15513 | 83379 | Klb | 1.472557 | 2.988164 | 1.672635 | 1.7865 | 0.037009 |
| 7713 | 16644 | Kng1 | 1.169921 | -3.46289 | 1.938748 | -1.78615 | 0.037038 |
| 18686 | 97402 | C86187 | 5.692828 | -1.39999 | 0.783887 | -1.78596 | 0.037053 |
| 4694 | 170952 | Prima1 | 1.407887 | 3.302567 | 1.849218 | 1.785926 | 0.037056 |
| 16117 | 243274 | Tmem132 | 36.57024 | 0.619129 | 0.347289 | 1.782752 | 0.037313 |
| 8013 | 68159 | Stx19 | 7.696619 | -1.44467 | 0.811779 | -1.77964 | 0.037568 |
| 16640 | 319472 | 9330158H | 1.380245 | 3.359117 | 1.887665 | 1.779509 | 0.037578 |
| 17622 | 277898 | Slc15a5 | 1.608091 | 2.925329 | 1.646416 | 1.776786 | 0.037802 |
| 5194 | 109620 | Dsp | 3.292626 | -3.01896 | 1.699422 | -1.77646 | 0.037829 |
| 15242 | 231045 | 4931409K | 2.461929 | 2.517772 | 1.418791 | 1.77459 | 0.037983 |
| 4268 | 71916 | Dus4l | 31.62414 | 0.669905 | 0.377729 | 1.773508 | 0.038072 |
| 23156 | 53381 | Prdx4 | 39.85758 | 0.608726 | 0.343379 | 1.772751 | 0.038135 |
| 7549 | 74478 | Snx29 | 5.995733 | 1.8886 | 1.065647 | 1.772257 | 0.038176 |
| 6917 | 69146 | Gsdmd | 12.68337 | -1.07813 | 0.608834 | -1.77081 | 0.038296 |
| 15001 | 24111 | Uts2 | 0.984767 | -3.92005 | 2.213772 | -1.77076 | 0.038301 |
| 17038 | 1.01E+08 | Gm21284 | 4.422535 | 1.95161 | 1.103412 | 1.768704 | 0.038472 |
| 21473 | 66952 | 2310030G | 16.39533 | 0.924472 | 0.522895 | 1.767988 | 0.038531 |
| 2517 | 17268 | Meis1 | 24.94317 | 0.777129 | 0.440005 | 1.766183 | 0.038683 |
| 4059 | 170720 | Card14 | 3.18818 | 2.370392 | 1.342914 | 1.76511 | 0.038773 |
| 2390 | 625131 | Vmn2r87 | 0.990495 | 3.579142 | 2.030077 | 1.763057 | 0.038945 |
| 22058 | 12836 | Col7a1 | 11.30412 | 0.976934 | 0.554396 | 1.762159 | 0.039021 |
| 20082 | 71082 | 4933416M | 1.175854 | -3.66206 | 2.080114 | -1.76051 | 0.039161 |
| 7959 | 69457 | Tmem45a | 1.173381 | -3.76273 | 2.138281 | -1.7597 | 0.03923 |
| 17538 | 16633 | Klra2 | 1.063528 | -3.78143 | 2.150782 | -1.75816 | 0.03936 |
| 4585 | 72350 | Zc2hc1c | 24.20042 | 0.714267 | 0.406324 | 1.757877 | 0.039384 |
| 3415 | 74038 | Brip1os | 40.27978 | 0.687064 | 0.391295 | 1.755874 | 0.039555 |
| 6342 | 72609 | 2700070H | 3.296874 | 2.028034 | 1.155438 | 1.755209 | 0.039612 |
| 15305 | 12615 | Cenpa | 12.34388 | -1.01759 | 0.581462 | -1.75006 | 0.040054 |
| 21530 | . | AI118078 | 9.960622 | 1.119489 | 0.640528 | 1.747758 | 0.040253 |
| 14068 | 1E+08 | Mup15 | 2.613597 | -2.51208 | 1.437674 | -1.74732 | 0.040291 |
| 9046 | 107971 | Frs3 | 9.480063 | -1.30225 | 0.747037 | -1.74322 | 0.040647 |
| 16657 | 74253 | Klrg2 | 1.508181 | -3.25149 | 1.866883 | -1.74167 | 0.040783 |
| 21875 | 235533 | Gk5 | 2.787526 | 2.291536 | 1.31628 | 1.740919 | 0.040849 |
| 11930 | 13537 | Dusp2 | 23.43773 | 0.831221 | 0.477623 | 1.740327 | 0.040901 |
| 14106 | 18405 | Orm1 | 3.687657 | -2.02379 | 1.163525 | -1.73936 | 0.040986 |
| 4514 | 69522 | 2310002D | 9.019958 | -1.28363 | 0.738328 | -1.73856 | 0.041056 |
| 2185 | 69181 | Dyrk2 | 8.825764 | -1.10043 | 0.633331 | -1.73753 | 0.041147 |
| 7280 | 634104 | Olfr287 | 6.575555 | -1.30067 | 0.748901 | -1.73677 | 0.041214 |
| 22970 | 27359 | Sytl4 | 1.071083 | 3.39614 | 1.958423 | 1.73412 | 0.041448 |
| 17757 | 21955 | Tnnt1 | 11.56296 | 1.066801 | 0.615267 | 1.733885 | 0.041469 |
| 19468 | 233836 | Slc5a11 | 4.959301 | 1.712054 | 0.987556 | 1.733626 | 0.041492 |
| 10333 | 226049 | Dmrt2 | 2.856923 | -1.99515 | 1.153733 | -1.7293 | 0.041878 |
| 13563 | 69772 | Bdh2 | 6.432811 | 1.357469 | 0.78607 | 1.726905 | 0.042092 |
| 9818 | 269033 | 4930503L1 | 39.46327 | 0.587169 | 0.340419 | 1.724838 | 0.042278 |
| 22885 | 668415 | Gm9159 | 20.44109 | 0.684649 | 0.397663 | 1.721683 | 0.042563 |
| 9121 | 106759 | Ticam1 | 18.52726 | 0.774282 | 0.450489 | 1.718758 | 0.042829 |
| 13000 | 404710 | Iqgap3 | 6.637513 | -1.43923 | 0.838008 | -1.71744 | 0.042949 |
| 22081 | 378954 | 3000002C | 10.43736 | -1.07533 | 0.626547 | -1.71628 | 0.043055 |
| 19044 | 320100 | Relt | 40.02008 | 0.623898 | 0.363663 | 1.715596 | 0.043118 |
| 14327 | 67305 | Gpx7 | 21.21474 | 0.727914 | 0.424309 | 1.715527 | 0.043124 |
| 10478 | 56642 | Ankrd2 | 3.8469 | -1.93616 | 1.128727 | -1.71535 | 0.04314 |
| 15789 | 27405 | Abcg3 | 2.989382 | -2.07239 | 1.209157 | -1.71392 | 0.043272 |
| 7663 | 68162 | A930003A | 2.228014 | 2.699019 | 1.576033 | 1.71254 | 0.043399 |
| 6813 | 239463 | Fam83a | 1.526974 | -3.31658 | 1.937498 | -1.71179 | 0.043468 |
| 22612 | 245424 | Gpr101 | 19.02032 | 0.72294 | 0.422484 | 1.711165 | 0.043525 |
| 22835 | 668257 | Dgat2l6 | 5.104625 | -1.51019 | 0.882709 | -1.71086 | 0.043553 |
| 14146 | 22178 | Tyrp1 | 1.980999 | 3.418805 | 2.000429 | 1.709036 | 0.043722 |
| 18 | 72481 | 2610203C | 14.93834 | 0.963628 | 0.564088 | 1.708293 | 0.043791 |
| 10945 | 67350 | 1700084E1 | 4.699644 | -1.89663 | 1.111106 | -1.70697 | 0.043913 |
| 3359 | 1E+08 | AI662270 | 4.470851 | -2.05545 | 1.204439 | -1.70656 | 0.043951 |
| 1556 | 320363 | D030045P | 1.27087 | -3.54073 | 2.077601 | -1.70424 | 0.044168 |
| 4186 | 209334 | Gen1 | 4.385375 | -1.97017 | 1.156639 | -1.70336 | 0.04425 |
| 18006 | 21946 | Pglyrp1 | 25.23645 | 0.647038 | 0.379931 | 1.70304 | 0.04428 |
| 10753 | 66774 | Carlr | 5.969406 | 1.402534 | 0.823854 | 1.702406 | 0.04434 |
| 22160 | 320256 | Dlec1 | 1.66968 | 3.238714 | 1.904501 | 1.700558 | 0.044513 |
| 2389 | 625109 | Vmn2r86 | 1.457361 | 3.203478 | 1.883783 | 1.700556 | 0.044513 |
| 8968 | 73504 | 1700071M | 1.899374 | -2.8697 | 1.688327 | -1.69973 | 0.044591 |
| 21576 | 13070 | Cyp11a1 | 11.44556 | 0.996358 | 0.586319 | 1.699346 | 0.044627 |
| 21723 | 102371 | Myzap | 10.30396 | -1.09627 | 0.645617 | -1.69803 | 0.044751 |
| 21080 | 14739 | S1pr2 | 19.96086 | 0.803156 | 0.473557 | 1.696008 | 0.044942 |
| 17284 | 94346 | Tmem40 | 10.80863 | 1.124961 | 0.663434 | 1.695665 | 0.044975 |
| 2174 | 70574 | Cpm | 36.04104 | 0.707883 | 0.41748 | 1.695609 | 0.04498 |
| 7644 | 21380 | Tbx1 | 6.68067 | 1.556357 | 0.91896 | 1.693606 | 0.04517 |
| 18502 | 668620 | Zfp936 | 1.204726 | 2.996854 | 1.769745 | 1.693382 | 0.045191 |
| 17356 | 232345 | A2m | 2.826634 | 2.677846 | 1.582232 | 1.692449 | 0.04528 |
| 3472 | 18559 | Pctp | 7.216951 | -1.22279 | 0.722529 | -1.69237 | 0.045288 |
| 22728 | 434797 | Gm5640 | 0.918458 | -3.61665 | 2.137174 | -1.69226 | 0.045298 |
| 7283 | 239652 | Zfp641 | 4.70462 | 1.72396 | 1.018917 | 1.691953 | 0.045327 |
| 9561 | 93875 | Pcdhb4 | 12.8619 | -1.06208 | 0.627796 | -1.69177 | 0.045345 |

| | | | | | | | |
|---|---|---|---|---|---|---|---|
| 2970 | 214384 | Myocd | 7.647356 | -1.36348 | 0.806081 | -1.69149 | 0.045372 |
| 1238 | 22283 | Ush2a | 1.557696 | -3.42596 | 2.02639 | -1.69067 | 0.04545 |
| 15589 | 56183 | Nmu | 1.181027 | -3.52598 | 2.087803 | -1.68885 | 0.045624 |
| 17597 | 14840 | Gsg1 | 3.671633 | -2.07159 | 1.228094 | -1.68683 | 0.045818 |
| 19942 | 11601 | Angpt2 | 9.992963 | -1.10688 | 0.657105 | -1.68448 | 0.046045 |
| 5455 | 218311 | Zfp455 | 20.52502 | 0.840053 | 0.499154 | 1.682953 | 0.046192 |
| 5594 | 64918 | Bhmt2 | 1.246328 | 3.483985 | 2.071035 | 1.682243 | 0.046261 |
| 9898 | 67105 | Timm21 | 24.50804 | 0.686139 | 0.407875 | 1.682228 | 0.046262 |
| 13354 | 22414 | Wnt2b | 7.002867 | -1.36423 | 0.8114 | -1.68133 | 0.04635 |
| 16895 | 171201 | Vmn1r21 | 0.927327 | 3.631321 | 2.160275 | 1.680953 | 0.046386 |
| 537 | 14659 | Glrp1 | 1.884937 | 2.671511 | 1.589497 | 1.680727 | 0.046408 |
| 2067 | 16545 | Kera | 1.014215 | 3.802537 | 2.262854 | 1.680416 | 0.046438 |
| 7648 | 12544 | Cdc45 | 7.206577 | -1.38514 | 0.824596 | -1.67978 | 0.0465 |
| 2515 | 1.03E+08 | Etaa1os | 8.12952 | -1.41583 | 0.843807 | -1.67791 | 0.046683 |
| 18048 | 11425 | Apoc4 | 0.96832 | -3.6008 | 2.146689 | -1.67738 | 0.046735 |
| 17402 | 317677 | C1s2 | 1.758581 | 3.117818 | 1.859102 | 1.677056 | 0.046766 |
| 12429 | 19223 | Ptgis | 9.392748 | -1.4488 | 0.864043 | -1.67677 | 0.046793 |
| 15712 | 75610 | 2010109A | 1.360782 | -3.6757 | 2.194779 | -1.67475 | 0.046992 |
| 19350 | 1E+08 | Snora23 | 1.724265 | 3.489393 | 2.08365 | 1.674654 | 0.047001 |
| 10417 | 77059 | 4931408D | 29.89811 | 0.645323 | 0.385358 | 1.674605 | 0.047006 |
| 15234 | 171209 | Asic3 | 2.41543 | 2.383731 | 1.423733 | 1.674282 | 0.047038 |
| 9546 | 12941 | Pcdha5 | 2.373399 | 2.554706 | 1.526695 | 1.673357 | 0.047129 |
| 5716 | 77318 | Ankrd55 | 2.407517 | 2.203437 | 1.317503 | 1.672434 | 0.047219 |
| 4781 | 1.01E+08 | DQ267102 | 1.124895 | -4.0172 | 2.402574 | -1.67204 | 0.047258 |
| 6073 | 218977 | Dlgap5 | 1.295095 | -2.88602 | 1.73072 | -1.66752 | 0.047705 |
| 14080 | 1E+08 | Mup16 | 1.158994 | -3.01609 | 1.81296 | -1.66363 | 0.048093 |
| 19475 | 71208 | 4933440M | 1.108029 | 3.706755 | 2.228867 | 1.663067 | 0.04815 |
| 27 | 1E+08 | Tcf24 | 1.908824 | -2.74335 | 1.650504 | -1.66213 | 0.048244 |
| 1339 | 320273 | B230208H | 3.840542 | -1.96084 | 1.181117 | -1.66015 | 0.048442 |
| 133 | 214854 | Neurl3 | 3.313765 | 2.194591 | 1.322318 | 1.659655 | 0.048492 |
| 13756 | 208890 | Slc26a7 | 10.62599 | 0.918697 | 0.553766 | 1.658999 | 0.048558 |
| 1898 | 432478 | Tmprss9 | 5.218968 | 1.479919 | 0.893601 | 1.656129 | 0.048848 |
| 12418 | 319684 | 5031425F1 | 1.397917 | -3.18864 | 1.926268 | -1.65535 | 0.048927 |
| 8594 | 433091 | Pnpla1 | 5.580194 | 1.603798 | 0.970691 | 1.652222 | 0.049245 |
| 8112 | 77037 | Mrap | 2.567276 | 2.638238 | 1.597138 | 1.651854 | 0.049282 |
| 17378 | 69810 | Clec4b1 | 1.360874 | 3.365001 | 2.03745 | 1.651575 | 0.049311 |
| 14390 | 100465 | Mob3c | 24.04854 | 0.717344 | 0.434557 | 1.650748 | 0.049395 |
| 5977 | 67580 | Lrrc18 | 6.330971 | 1.552482 | 0.940623 | 1.650482 | 0.049422 |
| 3027 | 327951 | Cyb5d1 | 1.222657 | -3.24642 | 1.969417 | -1.64842 | 0.049634 |
| 6521 | 77744 | Bora | 4.964099 | 1.600732 | 0.971509 | 1.647676 | 0.04971 |
| 15866 | 231603 | A630023P | 20.75552 | 0.744937 | 0.452355 | 1.646796 | 0.0498 |
| 5469 | 77857 | 9430065F1 | 2.940512 | -2.6329 | 1.599658 | -1.64591 | 0.049891 |
| 21842 | 12045 | Bcl2a1b | 8.504801 | 1.126803 | 0.684742 | 1.645589 | 0.049924 |

**Supplementary Table 3. The list of common DEGs in the mice treated with WT-syn and V40G-syn relative to those of PBS-treated mice.**

log2 [fold change] ≥ 0.5, $p < 0.05$.

**Normalized Log2 fc common genes**

| Overlapped gene | WT-syn log2FC | V40G log2FC |
|---|---|---|
| 2310030G06Rik | 1.07312746 | 0.924471862 |
| 2310034O05Rik | 3.102396596 | 3.705054709 |
| 4930550C14Rik | 1.409728406 | 1.395629914 |
| 4931440F15Rik | 0.845516002 | 0.780516674 |
| 4933412E12Rik | -1.05505078 | -1.024661903 |
| 4933413J09Rik | 4.305057367 | 3.724878206 |
| 4933432I09Rik | -1.373067538 | -1.185045534 |
| 4933433G19Rik | 1.515587181 | 1.29930337 |
| 9430014N10Rik | 1.710418192 | 1.795725565 |
| A2m | 4.404468353 | 2.677845853 |
| A930003A15Rik | 3.916488937 | 2.699019479 |
| Abi3bp | 0.811246732 | 0.79574469 |
| Accs | -1.06036089 | -1.305130832 |
| AI118078 | 1.64902888 | 1.119488938 |
| AI662270 | -3.785923319 | -2.055454071 |
| Ak7 | -1.818465028 | -2.506653249 |
| Angpt2 | -1.276554402 | -1.106879322 |
| Angptl3 | 2.25934004 | 2.423079335 |
| Ankar | -3.365355249 | -3.42626158 |
| Apoc1 | -1.144406808 | -1.522587141 |
| Arhgef5 | 1.004841812 | 1.242625125 |
| Arl11 | 2.151140918 | 2.273540332 |
| Atp10b | 2.578140233 | 2.54561727 |
| AU040320 | 0.806382975 | 0.840650163 |
| AV051173 | 2.496717924 | 2.447363827 |
| B230208H11Rik | -2.073522739 | -1.960835293 |
| BC018473 | 2.775671587 | 3.888746552 |
| Bcar3 | 0.612842038 | 0.600039871 |
| Bmp5 | 3.469789442 | 4.046776405 |
| Bora | 1.815461537 | 1.600731685 |
| Btla | 2.007875348 | 2.159219491 |
| C1qtnf6 | 2.029511338 | 1.952550158 |
| C630031E19Rik | 0.913247573 | 1.078050238 |
| C86187 | -1.323014383 | -1.399991194 |
| Cacna2d4 | -1.184499317 | -1.119487752 |
| Casp6 | 1.152770078 | 0.799049066 |
| Cbx2 | 1.705278279 | 1.131120236 |
| Ccdc150 | 3.057285313 | 4.207710336 |
| Ccdc162 | 3.293574959 | 2.523455494 |
| Ccdc89 | 4.312228765 | 3.721622499 |

| | | |
|---|---|---|
| Clec4b1 | 3.622751281 | 3.365000801 |
| Clic6 | 2.371193486 | 1.880032814 |
| Cog8 | 0.659218035 | 0.814529591 |
| Cxcl5 | 4.039697596 | 4.462393483 |
| D830013O20Rik | 3.605195071 | 3.705622623 |
| Dbh | -2.032451636 | -2.036770927 |
| Dmrt2 | -2.566260341 | -1.995150746 |
| Dnmt3b | -1.394379337 | -1.745518944 |
| Dyrk2 | -1.104264295 | -1.100430961 |
| E330011O21Rik | 2.31820512 | 3.298685255 |
| Fam83h | 0.985448643 | 1.800142728 |
| Fscn2 | 4.54091827 | 3.418517534 |
| Galnt4 | 0.927721426 | 0.997805048 |
| Gm15412 | 2.638190828 | 3.07066444 |
| Gm1564 | 5.888903658 | 2.768181042 |
| Gm3279 | 2.246982758 | 1.86307521 |
| Gm5136 | 1.334665839 | 1.803781827 |
| Gm5177 | 2.137629172 | 2.178536165 |
| Gm9159 | 1.259980007 | 0.684649251 |
| Gpr101 | 0.847672233 | 0.722939834 |
| Grin2a | 0.765081327 | 0.84546289 |
| H2-Aa | 1.496735606 | 1.280957047 |
| H2-Ab1 | 1.469208892 | 1.352051389 |
| H2-Eb1 | 0.859951803 | 0.953246932 |
| Hcst | -1.249662939 | -1.655799601 |
| Hgfac | 3.35736814 | 3.820214776 |
| Hpgd | 1.492976877 | 1.457656748 |
| Idi2 | 3.6271408 | 4.502545257 |
| Igfbp1 | -2.439496696 | -3.821888906 |
| Iqgap3 | -2.912529699 | -1.439227462 |
| Irf7 | -1.82522457 | -2.901907042 |
| Irx1 | 1.634803724 | 1.779444218 |
| Itga1 | 1.063454602 | 1.369269431 |
| Lgals9 | 0.648114796 | 0.665288312 |
| Lrrc29 | 2.000167974 | 2.484770549 |
| Mc4r | 0.681160175 | 0.76418193 |
| Nes | 0.744421606 | 1.045816641 |
| Neurl3 | 2.270184194 | 2.194591118 |
| Neurod4 | -1.482612851 | -1.865482542 |
| Ngfr | 1.266134644 | 1.376437669 |
| Oas1c | 1.544188571 | 2.368022813 |
| Pbld2 | 2.458606537 | 1.688539454 |
| Pcdhb4 | -1.10937505 | -1.062084162 |

| level | % area | |
|---|---|---|
| - | ~ 1.5 | No pathology |
| + | 1.5 ~ 3.0 | Mild pathology |
| ++ | 3.0 ~ 4.5 | Moderate pathology |
| +++ | 4.5 ~ 6 | Dense pathology |
| ++++ | 6 ~ | Severe pathology |

| | | 2 weeks | | | | | | 4 weeks | | | | | | 10 weeks | | | | | |
|---|---|---|---|---|---|---|---|---|---|---|---|---|---|---|---|---|---|---|---|
| Bregma | | PBS | | WT-syn | | V40G | | PBS | | WT-syn | | V40G | | PBS | | WT-syn | | V40G | |
| (mm) | | cont | ipsi | cont | ipsi | cont | ipsi | cont | ipsi | cont | ipsi | cont | ipsi | cont | ipsi | cont | ipsi | cont | ipsi |
| 0.86 | cingulate cortex | + | ++ | + | +++ | + | +++ | - | + | + | ++ | + | ++++ | - | - | - | - | - | - |
| | motor cortex | - | + | - | ++ | - | ++ | - | - | - | +++ | - | +++ | - | - | - | + | - | - |
| | somatosensory cortex | - | ++ | - | + | - | + | - | - | - | +++ | - | + | - | - | - | - | - | + |
| | insular cortex | - | ++ | - | ++ | - | ++ | - | - | - | ++++ | - | +++ | - | - | - | - | - | +++ |
| | piriform cortex | + | ++ | - | ++ | - | +++ | - | - | - | +++ | - | ++++ | - | - | - | - | + | ++++ |
| | claustrum | - | ++ | - | ++ | + | +++ | - | - | - | ++++ | - | ++++ | - | - | - | - | + | ++++ |
| | striatum | + | ++ | + | +++ | - | +++ | - | - | - | +++ | - | ++++ | - | - | - | - | - | ++ |
| | nucleus accumbens | ++ | +++ | - | ++++ | + | ++++ | - | - | - | ++ | - | +++ | - | - | - | + | - | +++ |
| -1.82 | retrosplenial cortex | + | + | + | + | ++++ | ++++ | - | + | - | - | - | - | - | - | - | - | - | - |
| | parietal cortex | - | + | + | ++ | ++++ | ++++ | - | - | - | - | - | - | - | - | - | - | - | - |
| | somatosensory cortex | - | ++ | - | + | ++ | ++++ | - | - | - | - | - | - | - | - | - | + | - | - |
| | rhinal cortex | - | ++++ | + | ++++ | ++ | ++++ | - | - | - | ++ | + | ++ | - | + | - | + | + | ++ |
| | piriform cortex | + | ++++ | + | ++++ | ++ | ++++ | - | - | ++ | ++++ | + | ++ | - | + | - | + | + | +++ |
| | endopiriform nucleus | + | ++++ | + | ++++ | ++ | ++++ | - | - | + | ++++ | + | +++ | - | + | - | + | ++ | ++++ |
| | hippocampus | + | ++ | +++ | ++++ | +++ | ++++ | - | - | - | - | - | + | - | - | - | - | - | + |
| | habenular nucleus | - | + | + | + | + | +++ | - | - | + | + | + | ++ | - | - | - | - | + | + |
| | hypothalamus | + | + | ++ | ++ | + | + | - | - | - | + | - | + | - | - | - | - | + | + |
| | basolateral amygdala | + | +++ | + | ++++ | ++ | ++++ | - | - | + | ++++ | + | ++ | - | + | - | - | ++ | ++++ |
| | central amygdala | - | +++ | + | ++++ | + | ++++ | - | + | + | ++++ | + | +++ | - | + | - | - | ++ | ++++ |
| -2.8 | retrosplenial cortex | - | - | ++ | ++++ | + | + | - | + | + | + | + | + | - | - | - | - | - | + |
| | visual cortex | - | + | - | ++ | - | + | - | - | - | + | - | + | - | - | - | + | - | - |
| | auditory cortex | - | + | - | ++ | - | ++ | - | - | - | + | - | ++++ | - | - | - | ++ | - | ++ |
| | rhinal cortex | - | ++ | + | ++++ | ++ | ++++ | + | + | + | ++++ | + | ++++ | - | - | - | + | + | ++++ |
| | piriform cortex | - | ++ | - | + | ++ | ++++ | - | + | + | ++ | + | ++++ | - | - | - | - | ++ | ++++ |
| | hippocampus | - | - | + | ++ | + | ++ | - | - | + | + | - | ++ | - | - | - | - | - | + |
| | antpretectal nucleus | - | - | + | ++ | - | - | - | - | - | + | - | - | - | - | - | - | - | - |
| | substantia nigra | - | - | - | + | - | - | - | - | - | - | - | - | - | - | - | - | - | - |
| | retomammillary nucleus | - | - | - | - | - | - | - | - | - | - | - | - | - | - | - | - | - | - |

**Supplementary Table 4. Representative profiles of IL-1β pathology in mouse brain regions.** Semiquantitative grading of IL-1β-positive cells in mice injected with PBS, WT-syn fibrils and V40G multimers. IL-1β expression levels in each region were evaluated in brain sections from six animals on a scale of 1 to 5, where level 1 is the lowest in the area with less than 1.5% and level 5 is the highest in the area with at least 6%, and the average values for each region were rounded up or down.

**Supplementary Table 5. Primers used in this study**

| Gene | | Sequence of Primer | Application |
|---|---|---|---|
| Human α-synuclein V40G variant | Forward | GGTGTTCTCTATGGCGGCTCCAAAACCAAG | Site-directed mutagenesis |
| | Reverse | CTTGGTTTTGGAGCCGCCATAGAGAACACC | |
| Mouse TNF-α | Forward | CCTCTTCTCATTCCTGCTTGTTGG | qRT-PCR |
| | Reverse | GGTGGTTTGTGAGTGTGAGGG | |
| Mouse IL-1β | Forward | ATCCCAAGCAATACCCAAAGAAGAA | qRT-PCR |
| | Reverse | GTGAAGTCAATTATGTCCTGACCAC | |
| Mouse GAPDH | Forward | AGAAGGTGGTGAAGCAGGCATC | qRT-PCR |
| | Reverse | CGAAGGTGGAAGAGTGGGAGTTG | |

**Supplementary Table 6. Antibodies used in this study**

| Name of Antibody | Manufacturer | Catalog # | Dilution used |
|---|---|---|---|
| Mouse monoclonal anti-α-synuclein (Syn-1) | BD Biosciences | Cat#610787 | 1:1500 (WB)<br>1:500 (IHC) |
| Mouse monoclonal anti-human α-synuclein (LB509) | Abcam | Cat# ab27766 | 1:1000 (WB) |
| Rabbit monoclonal anti-NLRP3 (EPR23094-1) | Abcam | Cat# ab263899 | 1:1000 (WB) |
| Rabbit polyclonal anti-phospho-α-synuclein | Abcam | Cat# ab59264 | 1:500 (IHC) |
| Rabbit polyclonal anti-phospho-α-synuclein | Biolegend | Cat#825701 | 1:500 (IHC) |
| Rabbit monoclonal anti-phospho-α-synuclein (EP1536Y) | Abcam | Cat# ab51253 | 1:500 (IF)<br>1:50 (Immuno-EM) |
| Rabbit polyclonal anti-Iba-1 | Wako | Cat#019-19741 | 1:200 (IHC) |
| Goat polyclonal anti-Iba-1 | Novus Biologicals | Cat# NB100-1028 | 1:200 (IF) |
| Rabbit polyclonal anti-GFAP | Abcam | Cat# ab7260 | 1:500 (IHC) |
| Mouse monoclonal anti-GFAP (GF5) | Abcam | Cat# ab10062 | 1:500 (IF) |
| Rabbit polyclonal anti-tyrosine hydroxylase | Abcam | Cat# ab112 | 1:2000 (IHC) |
| Rabbit polyclonal anti-tyrosine hydroxylase | Millipore | Cat# AB152 | 1:1000 (IHC) |
| Rabbit polyclonal anti-IL-1β | Abcam | Cat# ab9722 | 1:200 (IHC, IF) |
| Rabbit polyclonal anti-TNF-α | Abcam | Cat# ab6671 | 1:200 (IHC) |

| | | | |
|---|---|---|---|
| Rat monoclonal anti-CD4 | BD Biosciences | Cat#553727 | 1:200 (IHC) |
| Rat monoclonal anti-CD8a | BD Biosciences | Cat#553027 | 1:200 (IHC) |
| Mouse monoclonal anti-NeuN | Millipore | Cat# MAB377 | 1:500 (IF) |
| Rabbit monoclonal anti-ASC (D2W8U) | Cell Signaling Tech | Cat# 67824 | 1:100 (IF) |
| Alexa fluor 488-anti-mouse secondary antibody | Jackson Immunoresearch Laboratories | Cat#115-545-062 | 1:200 (IF) |
| Alexa fluor 488-anti-goat secondary antibody | Jackson Immunoresearch Laboratories | Cat#705-545-147 | 1:200 (IF) |
| Rhodamine red-X-anti-rabbit secondary antibody | Jackson Immunoresearch Laboratories | Cat#111-295-144 | 1:200 (IF) |
| HRP-anti-mouse secondary antibody | Bio-Rad | Cat#170-6516 | 1:500 (IF) |
| HRP-anti-rabbit secondary antibody | Bio-Rad | Cat#170-6515 | 1:500 (IF) |
| HRP-anti-rat secondary antibody | Bio-Rad | Cat#5204-2504 | 1:500 (IF) |
